# Understanding the association between adverse childhood experiences and subsequent attention deficit hyperactivity disorder: A systematic review and meta‐analysis of observational studies

**DOI:** 10.1002/brb3.2748

**Published:** 2022-09-06

**Authors:** Ning Zhang, Man Gao, Jinglong Yu, Qiang Zhang, Weiguang Wang, Congxiao Zhou, Lingjia Liu, Ting Sun, Xing Liao, Junhong Wang

**Affiliations:** ^1^ Department of Pediatrics, Dongzhimen Hospital Beijing University of Chinese Medicine Beijing China; ^2^ Institute of Information on Traditional Chinese Medicine China Academy of Chinese Medical Sciences Beijing China; ^3^ School of Traditional Chinese Medicine Beijing University of Chinese Medicine Beijing China; ^4^ Center of Evidence Based Traditional Chinese Medicine, Institute of Basic Research in Clinical Medicine China Academy of Chinese Medical Sciences Beijing China

**Keywords:** adverse childhood experiences, attention deficit hyperactivity disorder, meta‐analysis, systematic review

## Abstract

**Objectives:**

Attention deficit hyperactivity disorder (ADHD) is a common neurodevelopmental disorder in childhood, which may be related to adverse childhood experiences (ACEs). Our study aims to explore the association between ACEs and subsequent ADHD, and analyze the potential moderators.

**Methods:**

Literature search was conducted by a combined computer‐assisted and manual method. Studies were included if they had reported the association between ACEs and subsequent ADHD. Overall estimates of odds ratios (ORs) were obtained using random‐effects meta‐analyses, meta‐regressions and further stratified analyses were conducted to examine potential moderator variables.

**Results:**

Totals of 70 studies involving nearly 4 million participants from among 6,452 unique articles were included. In the primary analyses, ACEs were found to be associated with subsequent ADHD (OR = 1.68, 95% CI: 1.54–1.83), and the negative effects of different forms of ACEs for ADHD were nonequivalent. Such as lived in the stepfamily, been adopted or fostered, and experienced sexual abuse were more deleterious than others. It was found that individuals who had experienced multiple ACEs or who are female were more vulnerable to ADHD.

**Conclusions:**

The findings provide critical evidence for understanding the association between ACEs and ADHD. ACEs could increase the susceptibility of ADHD, especially for individuals who ever experienced multiple ACEs and females.

## 1 INTRODUCTION

Attention deficit hyperactivity disorder (ADHD) is a common neurodevelopmental disorder typically characterized by symptoms in the inattentive, hyperactive, or (and) impulsive domains (Posner et al., 2020). This disease mainly diagnosed in childhood, which is estimated to affect approximately 5.5% of children and adolescents and 2.1%–3.1% of adults (Erskine et al., 2017; Simon et al., 2018). Individuals with ADHD frequently suffer from multiple psychiatric comorbidities, including externalizing disorders (e.g., Oppositional Defiant Disorder [ODD], Conduct Disorder [CD]) and internalizing disorders (e.g., Anxiety Disorder [AD], Depression Disorder [DD]) (Humphreys et al., 2012; Mitchison & Njardvik, 2015). This embarrassing situation not only increases the risk of educational and occupational failure (Brook et al., 2013; Jangmo et al., 2019), accidents (Vaa, 2014), and criminality (Retz et al., 2021), but also imposes a substantial burden on social economy and stability (Erskine et al., 2014). Given the multiple negative impacts of ADHD on the major global public health problem, there is a pressing need to identify factors that contribute to risk for ADHD.

Although the etiology and pathology have not been clearly identified yet, ADHD is recognized as a highly intergenerational and multifactorial psychobehavioral disorder, which arises from several genetic and environmental risk factors that act in conjunction (Faraone et al., 2015). Adverse childhood experiences (ACEs) have been found to be probably significant predictors of many health conditions (Hughes et al., 2017). ACEs are defined as experiences that threaten the child's bodily, familial, or social safety or security (Jones et al., 2020), range from broad categories of experiencing or witnessing violence, maltreatment, bullying, crime or discrimination, to growing up in a dysfunctional household with parental separation, mental health problems, and economic disadvantage (Suglia et al., 2018). These negative experiences have wide‐ranging effects on the neuroendocrine, immune, and metabolic systems, and subsequently trigger the onset and development of ADHD (Berens et al., 2017).

Specifically, ACEs are able to induce toxic stress, which is capable of inducing significant changes in development of the central nervous system (CNS) and mainly concentrated in essential stress and emotion regulation related brain regions, such as prefrontal cortex, amygdala, and hippocampus (Calem et al., 2017; McLaughlin et al., 2019). The abnormal “top‐down” effects of these brain regions for attentional and behavioral control may cause ADHD symptoms (Katsuki & Constantinidis, 2013; Liu et al., 2020). Furthermore, ACEs may sensitize the mesostriatal dopamine system to later challenges, and this neurotransmitter system is one of the most important targets of methylphenidate (Dahoun et al., 2019; del Campo et al., 2011). ACEs can also make the hypothalamic‐pituitary‐adrenal (HPA) axis hyper‐ or hypo‐ reactivity and generate downstream responses (Koss & Gunnar, 2018), including neurotoxicity, inflammation, and certain bioactive factors metabolism dysregulation (Chang et al., 2020; Rose et al., 2010). More importantly, there might be a pathway for the perpetuation of adversity, exposure to ACEs increases ADHD susceptibility, and ADHD patients are more likely to suffer from more ACEs similarly (Lugo‐Candelas et al., 2021).

Over the past three decades, numerous observational studies have been carried out worldwide with the aim of clarifying the association between ACEs and health conditions, with the association between ACEs and ADHD being an important component. The results among these studies may be highly variable, and different ACEs may have varieties of negative psychological effects (Vachon et al., 2015). Furthermore, the association between ACEs and ADHD may also be strengthened or weakened by genetic background and developmental stage. Because there are significant differences in the etiology, pathology, and clinical presentation of ADHD in childhood or adolescence and ADHD in adulthood (Schoeler et al., 2019; Zwicker et al., 2020). Most existing evidence‐based studies, however, were based on ADHD‐related behavioral problems assessed by questionnaire, such as the Child Behavior Checklist, and did not indicate clinically meaningful differences in diagnosis (Juffer & van Ijzendoorn, 2005; Maguire et al., 2015; Malvaso et al., 2021; Qu et al., 2018; Wu et al., 2019). The association between ACEs and ADHD that some evidence supports to a certain extent, has a sound theoretical basis. Therefore, to qualitatively and quantitatively assess the association between ACEs and ADHD using systematic review and meta‐analysis is critical to understand the nature of the influence of ACEs on the onset of ADHD.

## 2 METHODS

### 2.1 Registration of protocol

This systematic review and meta‐analysis was conducted according to the Meta‐analysis of Observational Studies in Epidemiology (MOOSE) guidelines and preferred reporting items for systematic reviews and meta‐analyses (the PRISMA statement) (Moher et al., 2009; Stroup et al., 2000). The protocol of this study was registered in advance on the International Prospective Register of Systematic Reviews (PROSPERO) (ID: CRD42020202041).

### 2.2 Search strategy

Potentially relevant studies were identified by computer‐assisted literature searches in the Embase, Medline (Ovid), PsycINFO (Ovid), CINAHL (EBSCOhost), and Web of Science in August 2020. Moreover, the reference lists of potentially eligible articles and systematic review papers were hand‐searched. Medical subject headings and free text terms of ACEs, ADHD, and study design of interest were used. Searches were updated in June 2021, and example of search strategy in Ovid Medline was available in Appendix 1.

### 2.3 Inclusion and exclusion criteria

Original studies were included if they were cohort studies, case‐control studies, or cross‐sectional studies that were published in English and reported: (1) Dichotomous or continuous measurement of any form of ACEs. (2) Diagnosis of subsequent ADHD or ADD according to any standardized criteria, such as DSM‐III, DSM‐III‐R, DSM‐IV, DSM‐IV‐TR, DSM‐5, or ICD‐10; or diagnostic information for ADHD could be obtained from reliable records, like vaccine‐related records, clinic registers, hospital records, and the like; or self‐report and informed guardian‐reported diagnosis for ADHD made by a professional. (3) At least the effect estimates with 95% confidence intervals (95% CIs) or standard error (SE) were reported, including odds ratio (OR), relative risk (RR), or hazard ratio (HR). (4) A non‐ADHD control group or a nonexposed control group was set up in the original study.

The following types of studies were excluded: (1) reported ADHD‐related symptoms, rather than any ADHD diagnosis; (2) studies in which did not specifically report any risk estimates for the outcome of our interest; (3) duplicate reporting on findings from the same population; (4) studies that did not provide detailed information, and the author did not give us feedback or could not provide detailed information to us. It is important to note that we also excluded these studies in which the diagnosis was only based on medication records. Since these studies may contribute to underestimating the diagnosis of ADHD or ADD as only about 60% of ADHD patients are treated with pharmacotherapy (Danielson et al., 2018) and there may be another known and unknown interconnections between ACEs and ADHD medication (Forster et al., 2017; Tang et al., 2021).

### 2.4 Data extraction and quality assessment

Duplicate records were removed after the search results were imported into Endnote X7. Titles and abstracts, and full texts of the potentially relevant records were independently screened by two researchers (Jinglong Yu, Qiang Zhang). A third researcher (Man Gao) checked the included literature.

A validated Excel table in prestudy was used to extract relevant data from eligible studies by two trained researchers (Lingjia Liu, Ting Sun). For all studies, we extracted information on the name of the first author, year of publication, effect sizes, and moderator codes, all of which were checked by Weiguang Wang. Disagreements were solved by the reviewers’ consensus (with Xing Liao and Junhong Wang).

As recommended by Cochrane Handbook of Systematic Reviews, the Newcastle‐Ottawa Quality Assessment Scale (NOS) was used for quality assessment of eligible cohort studies and case‐control studies, and an adapted version for cross‐sectional studies (Beran & Lupart, 2009). It is important to note that we rated the quality of cross‐sectional study as 2 points and 1 point, respectively, if ADHD was diagnosed by blinded or nonblinded diagnostic interviews and validated scales or questionnaires based on accepted diagnostic criteria, because ADHD is usually not diagnosed using laboratory methods as a mental disorder.

### 2.5 Statistical analysis

The effect estimates that generated from binary variables were converted to ORs, which were used as the common measurement of the association between ACEs and subsequent ADHD. For any eligible study, the average OR was calculated if the relationships between various forms of ACEs and ADHD were reported. Since RRs and ORs provide similar estimates of risk when the outcome was rare (<10%), and HRs were similar to RRs (McNutt et al., 2003; VanderWeele, 2020); thus, we approximated the adjusted RRs and HRs provided by the included studies to be equivalent to the adjusted ORs. For continuous variables, the total effect size of the standardized mean difference was calculated. The effect sizes were pooled using a random‐effects model, in which heterogeneity was taken into account (Borenstein et al., 2010). Standard Cochran's *Q* test and *I* ^2^ statistic were calculated to assess statistical heterogeneity that reflects real differences in effect sizes, with an *I* ^2^ values of 25% or less, near 50%, and more than 75% that could be considered low, moderate, and high, respectively. A meta‐regression was conducted to explore whether demographic factors (including gender composition, ADHD classification by period, and setting of location) and methodological factors (including sample size, study type, study design, study quality, effect estimation, and measures of ADHD or ACEs) across eligible studies moderated the association between ACEs and subsequent ADHD. When high heterogeneous was observed and the number of included studies was not less than 10, targeted stratified analysis was performed if a moderator mediated the association. The funnel plot was used to evaluate the possible publication bias, and further with the Begg's tests as recommended (Begg & Mazumdar, 1995). Leave‐one‐out method was used for sensitivity analysis, and the trim and fill method was conducted if the funnel plot revealed significant asymmetry and Begg's test identified the evidence of publication bias. All statistical analyses were conducted using Stata version 15.0, and *p* values were two‐tailed, with a significance level of .05.

## 3 RESULTS

### 3.1 Study inclusion

**FIGURE 1:**
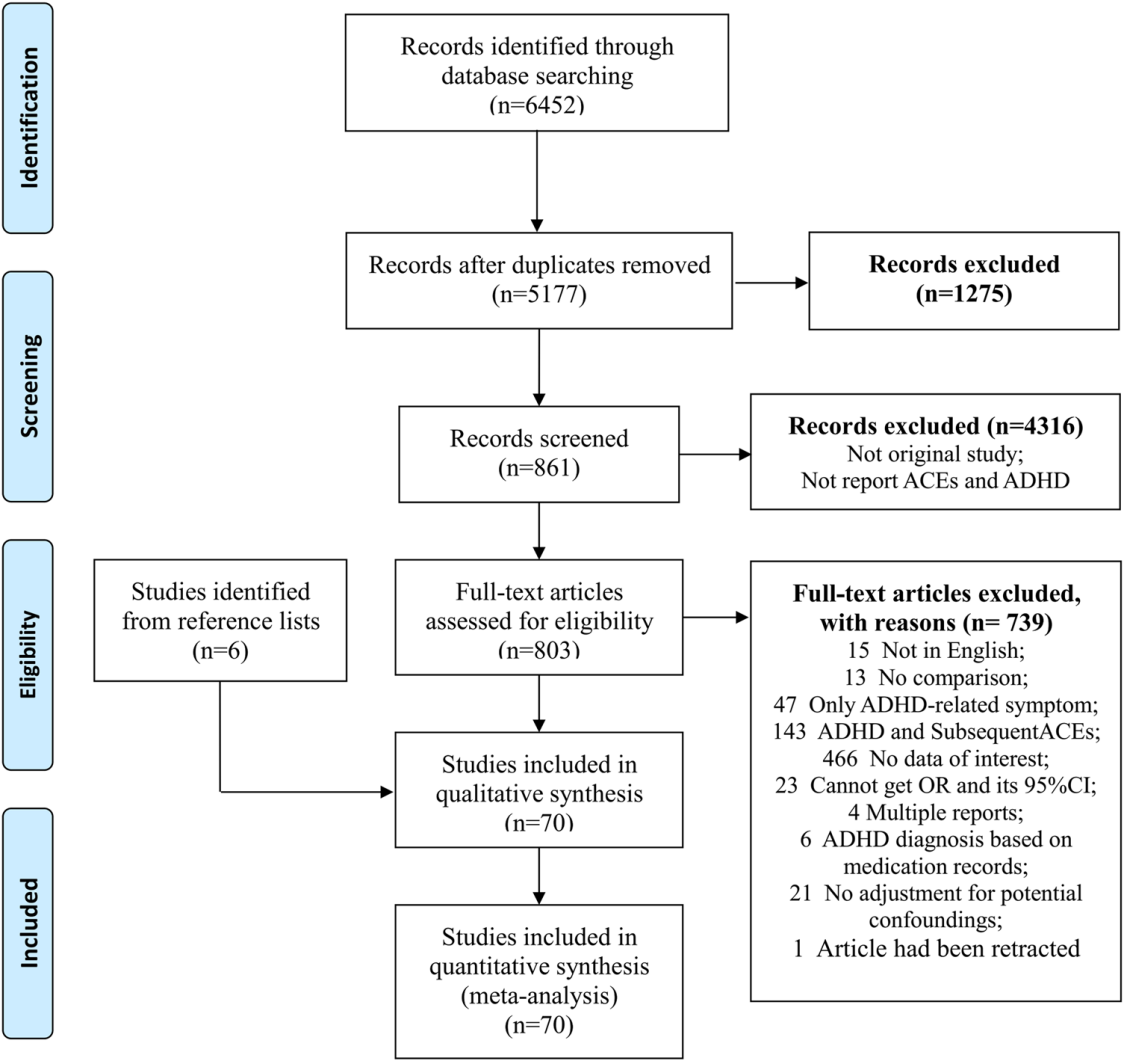
PRISMA flow chart

The literature searches identified 6452 unique records, which contained 1275 duplicates records, and 861 potential relevant articles were selected for full‐text screening by reading titles and abstracts. Among these articles, 803 were excluded and the reasons were presented in Figure 1. Moreover, six articles were identified through screening the reference lists in included studies. A total of 70 independent studies were eligible for inclusion and finished the quality assessment.

### 3.2 Study characteristics

Eligible studies, with data on no less than 3,901,712 individuals, were mainly conducted in Europe, North America, Asia, and Oceania, besides three studies in Africa, one study in South America, and two multicenter studies conducted in Europe, North America, and Asia.

As shown in Appendices 2–4, all the included studies were cohort studies, case‐control studies, or cross‐section studies. Of all included studies, 30, 30, and 10 studies reported associations of ACEs with childhood ADHD, adult ADHD, and childhood and adult ADHD, respectively. Measures for the diagnosis of ADHD and ACEs in these studies also differed, included diagnostic interview/questionnaire/scale based on ICD or DSM diagnostic criteria, record‐based information, or self‐/caregiver‐report of the diagnosis by a health professional, clinician or psychiatrist. However, the sample size and study quality varied widely across the included studies. Specifically, all studies had sample sizes ranging from 58 to 870,017, with 14 studies containing fewer than 500 participants. The methodological quality of all studies scored between 3 and 8, containing 7 unsatisfactory studies, 27 satisfactory studies, and 36 good studies. The references of all included studies were presented in Appendix 5.

## 4 OUTCOMES

### 4.1 All 17 forms of common ACEs

**FIGURE 2:**
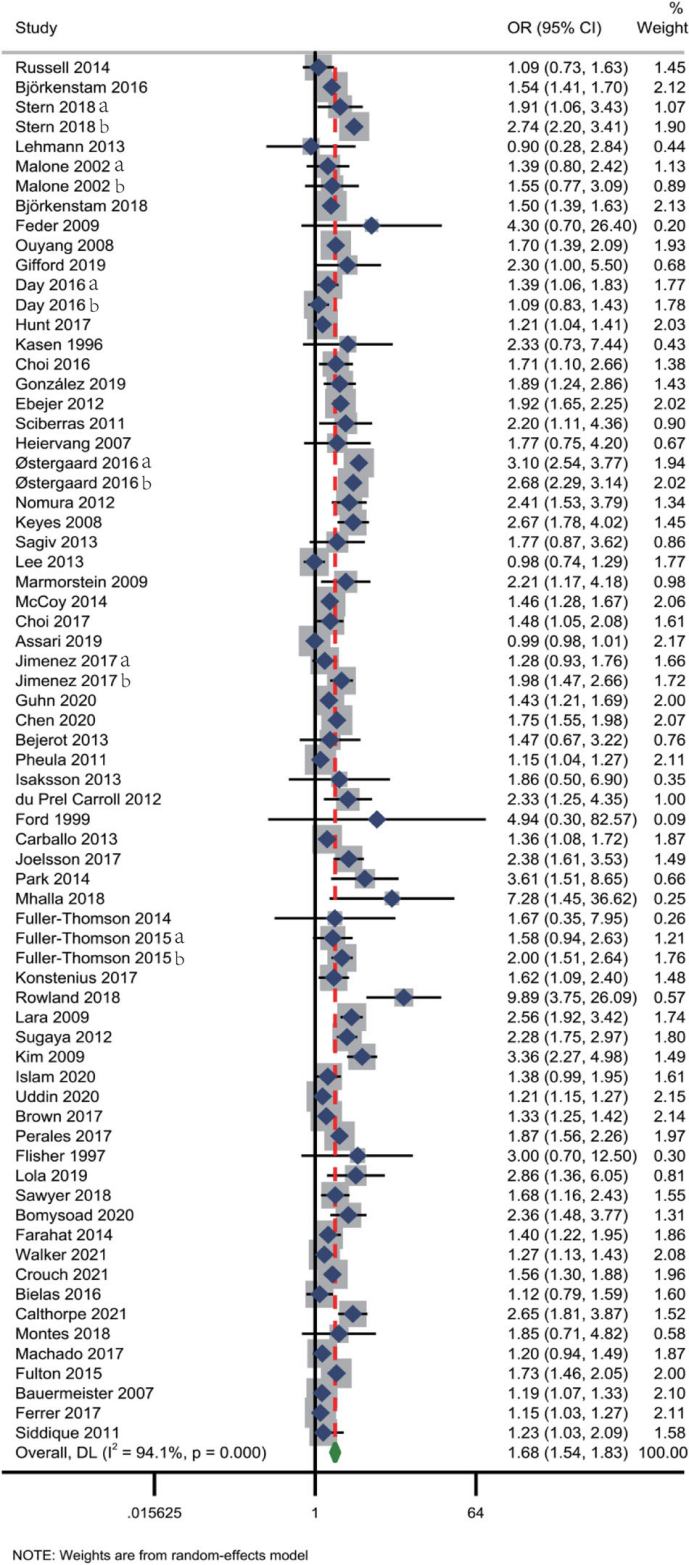
Meta‐analysis for the association between any forms of ACEs and ADHD

Figure 2 presents the results of random‐effects meta‐analysis for the association between ACEs and ADHD, and 70 studies were included in the analysis. The result indicated that individuals who had experienced any forms of ACEs were more susceptible to ADHD than individuals unexposed to ACEs (OR = 1.68, 95% CI: 1.54–1.83, *z* = 11.894, *p* < .05), and high heterogeneity was observed (*Q* (69) = 1173.34, *I* ^2^ = 94.1%, *p* < .05).

### 4.2 Each form of common ACEs

As shown in Appendices 6 and 7, which presents the results of meta‐analyses using random‐effects model for the associations between each form of ACEs and ADHD, individuals who experienced any form of ACEs were significantly more likely to develop ADHD than individuals without a history of ACEs.

Specifically speaking, medium‐size association (1.50 ≤ OR < 3.00) were detected between exposure to physical abuse, sexual abuse, emotional abuse, childhood neglect, economic adversity, stepfamily/adoption/foster placement, familial criminality/incarceration, household substance abuse, household mental illness, family discord, bullying, or other violence and ADHD. Low‐size association (1.00 < OR < 1.50) between other forms of ACEs and ADHD, such as single parent/parental separation/divorce, familial death, domestic violence, discrimination, and accident/injury/illness trauma.

### 4.3 Number of ACEs and ADHD diagnosis

**FIGURE 3:**
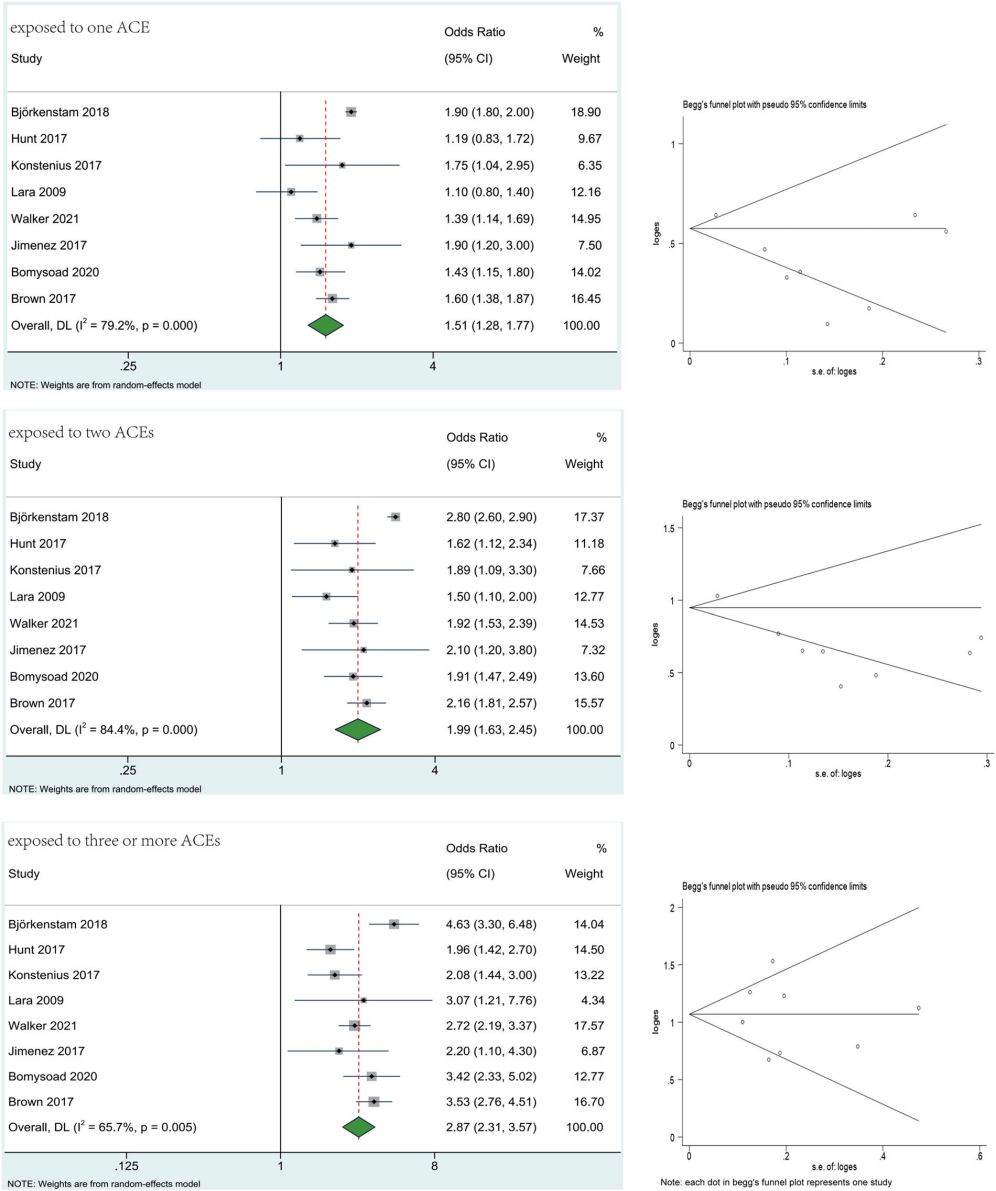
Results of meta‐analysis for the association between the number of ACEs and ADHD

We pooled the results of studies that investigated the association between the number of ACEs exposed and ADHD, and the results were presented in Figure 3 and Appendix 8. Meta‐analysis revealed that individuals who had experienced ACEs (one ACE, two ACEs, and three or more ACEs) were more susceptible to ADHD compared with those unexposed to ACEs, with the pooled ORs were 1.51 (95% CI: 1.28–1.77, *z* = 5.011, *p* < .05), 1.99 (95% CI: 1.63–2.45, *z* = 6.630, *p* < .05), and 2.87(95% CI: 2.31–3.57, *z* = 9.532, *p* < .05), correspondingly.

### 4.4 Effect of ACEs on the core symptoms and the subtype of ADHD

The influences of ACEs on the core symptoms and the type of ADHD was also analyzed to further reveal the association between ACEs and ADHD. The results of meta‐analysis showed that ACEs are associated with higher scores of ADHD symptom (the pooled effect sizes ranged from 0.20 to 5.96) and increased vulnerability to different subtypes of ADHD (the pooled ORs ranged from 1.44 to 2.12). The detailed results of meta‐analysis could be found in Appendices 9 and 10.

### 4.5 Moderator analysis

Significant heterogeneity was observed in meta‐analyses, except for the meta‐analyses for the association between sexual abuse, familial death, discrimination, or accident/injury/illness trauma, and ADHD. The Knapp–Hartung random‐effect meta‐regressions for moderators that mentioned above were conducted to explain variation in effect sizes, and the results were presented in Appendices 11 and 12.

### 4.6 Publication bias

**FIGURE 4:**
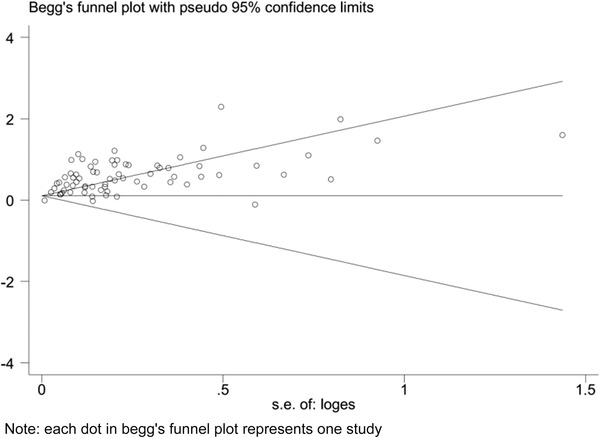
Begg's funnel plot of meta‐analysis for the association between any forms of ACEs and ADHD

Substantial asymmetry was observed in the visual inspection of the funnel plots, and the evidence of substantial publication bias was identified by Begg's test in meta‐analysis for the association between all forms of common ACEs and ADHD. As shown in Figure 4, visible asymmetry and potential publication bias were detected in funnel plot and Begg's test (*p* < .05).

### 4.7 Sensitivity analysis

Leave‐one‐out method sensitivity analyses revealed that no single study could independently significantly influence the pooled OR value of the association between all 17 forms of ACEs and the diagnosis of ADHD. However, González et al. (2019), Ouyang et al. (2008), and Lara et al. (2009) may statistically significantly influence the results of meta‐analyses for emotional abuse, childhood neglect, and accident/injury/illness trauma, correspondingly. In addition, we found a significant decrease in the pooled ORs of the meta‐analyses of the associations between discrimination or other violence and ADHD.

**FIGURE 5:**
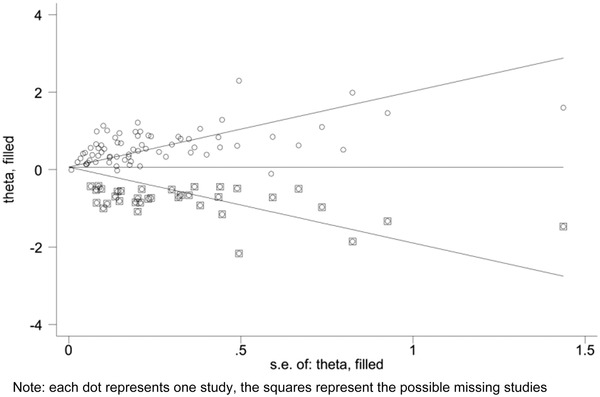
Trim and fill method sensitive analysis of meta‐analysis for the association between any forms of ACEs and ADHD

Trim and fill method sensitivity analysis showed that no statistically significant changes in the results of meta‐analysis for all 17 forms of ACEs in relation to any ADHD diagnosis after filled the 35 possible missing studies, and the details of trim and fill method sensitivity analysis were presented in Figure 5 and Appendix 13.

## 5 DISCUSSION

To our knowledge, this is the first systematic review and meta‐analysis that comprehensively examined existing observational studies to assess the association between ACEs and ADHD. Following literature searches, we finally identified 64 published journal articles, including 70 independent studies involving nearly 4 million participants. It was found that individuals who experienced any form of ACEs had a 1.68‐fold increased vulnerability of ADHD, compared to those without a history of ACEs.

Although we included observational studies that adjusted for potential confounders, significant heterogeneity was observed, which may pose risk of residual confounding. ADHD was revealed to have a high degree of genetic predisposition with gender difference and prevalence declines with age in the general population. We used the gender composition of the study population, the period for ADHD diagnosis, and the continent in which the studies were conducted as demographic moderators consequently. Despite thoroughly examining the potential mediation to influence the association between ACEs and ADHD, no evidence supported that the location of studies moderated the size of the obtained effects. In contrast, we found that individuals who had suffered ACEs in studies with a proportion of males below inferior quartile (IQ) were more likely to be diagnosed with ADHD, compared to those above IQ which calculated based on detailed data provided by included studies. This phenomenon may be correlated with sex differences in neurodevelopment and sensitivity to ACEs (Bath, 2020) and could be explained further by the fact that females may receive greater levels of ACEs and adopt low positive coping styles (Keller et al., 2019; Wan et al., 2020). The period of ADHD diagnosis might moderate the association between ACEs and ADHD, the pooled effect size of studies that reported only childhood or adulthood ADHD were 1.65 and 1.57, which were lower than studies that reported the association between ACEs and ADHD in both childhood and adulthood.

Additionally, we attempted to trace and analyze the heterogeneity through independent meta‐regressions for methodological characteristics, including sample size, indicator of effect estimate, study design and type, measures of ACEs and ADHD, and study quality. Since the eligible studies varied widely in these respects, which may mediate the association between ACEs and ADHD. The findings of meta‐regressions did not support the hypothesis that study quality affects the association between ACEs and ADHD and confirm the mediating roles of these other methodological moderators mentioned above. Sample size may have a positive correlation effect on the association, as poor association between ACEs and ADHD was observed in the subgroup of studies with sample sizes below IQ. This finding reflects the limitations of small sample studies that the bias caused by sampling error and sensitivity of logOR to sample size restricts sharp inferences from small samples to large populations (Lin, 2018; Tipton et al., 2016). The random‐effects model increases the weight of the small sample study and the result are more conservative than the fixed‐effects model (Nikolakopoulou et al., 2014), and no statistical difference was observed between the results of fixed‐effects and random‐effects or in stratified analysis for sample size. Only low‐size effects were identified in the subgroup of self‐/guardian‐reported ACEs or ADHD, while medium‐size effects in other subgroups. The data collected based on self‐/guardian‐reported may have been subject to recall biases, because the recall lengths varied from a few months to a dozen years and information collected by this method may be limited (Colman et al., 2015; Mannuzza et al., 2002). Stratified analyses for effect estimate, study design, and type did not provide sufficient evidence for mediated effects, although the mediated effects are theoretically and methodologically feasible.

We had examined the effects of 17 different forms of ACEs, since ACEs have a deleterious effect on health outcomes that is nonequivalent and specific, which means each form of childhood adversities has a specific effect on ADHD and some forms of ACEs are more harmful than others (Vachon et al., 2015). Single parent/parental separation/divorce, familial death, domestic violence, discrimination, or accident/injury/illness trauma were associated with lower‐level effects (1.00 < OR < 1.50) than other forms of ACEs (OR ≥ 1.50). While leave‐one‐out method sensitivity analyses uncovered significant variation in the results of the association between child sexual abuse, neglect, discrimination, other violence, or accident/injury/illness trauma and ADHD. Due to fewer studies included and higher heterogeneity, it is not enough to draw convincing conclusions for these forms of ACEs based on the existing evidence.

Dose–response meta‐analysis requires a sufficient amount of complete data (Orsini et al., 2012), the necessary data in many original studies has not yet been provided, and the results calculated by some estimation methods often vary from the original data. And almost all eligible studies have not comprehensively analyzed each form of ACEs, and certain form of ACEs (e.g., sex abuse) is a rare event almost always cooccurrence with other forms of ACEs. Hence, the real number of ACEs ever exposed may be greater than reported, despite the fact that some studies have analyzed the association between multiple ACEs and ADHD. This rarity and lopsided cooccurrence feature is likely to result in overestimation of the effect size to reduce the convincingness of the subsequent results in linear and nonlinear meta‐regression. After prudent consideration, we chose to stratified analyses based on the number of ACEs, rather than a dose–response meta‐analysis according to the protocol. The findings indicated that exposure to one, two, and three or more ACEs increased the vulnerability of ADHD by 1.51, 1.99, and 2.87 times, suggesting that individuals exposed to multiple ACEs are more likely to develop ADHD. Analysis of the effects of ACEs on specific core symptoms and subtypes of ADHD will contribute to further understanding of the association between ACEs and ADHD.

This study contained a large population and wide geographical variation that make the results representative, and targeted meta‐regressions and subsequent stratification and sensitivity analyses enabled the findings to be more accurate and reliable. Potential limitations should be noted: First, considerable numbers of cross‐sectional and case‐control studies were included, and the information on exposure to ACEs was collected when or after assessment of ADHD. Strong causal claims cannot yet be made regarding the role of ACEs in the morbidity of ADHD. Second, fewer included studies in the meta‐analyses for the association between some forms of ACEs and ADHD may not enable reliable and convincing conclusions to be drawn in these aspects. Third, the search strategies might have not covered all possible forms of ACEs and no grey literature database was retrieved specially, all of which may lead to bias. Fourth, the publication bias reduces the persuasiveness of this study, in spite of the fact that we confirmed the stability of the results by the leave‐one‐out method and the trim and fill method sensitivity analyses.

In addition to all of these, we believe that several critical issues need to be explored in depth in future research. Most importantly, the underlying protective factors that allow some individuals exposed to ACEs to achieve better than expected adaptation outcomes need to be clarified. Next, comorbidities in ADHD occur frequently, affecting the prognosis of ADHD, and so little knowledge is already acquired about whether and how ACEs might affect comorbidities in ADHD. Finally, there is a paucity of research on the association between special forms of ACEs and severity and clinical manifestation of subsequent ADHD. Research in this field has positive implications for the prevention and treatment of ADHD given the specificity of the toxic effects of ACEs.

## 6 CONCLUSIONS

This study indicates a negative effect of ACEs on ADHD, with the effect size varying depending on the form of ACEs. Our findings support the hypothesis that ACEs are associated with ADHD to a certain extent, especially for individuals who ever experienced multiple ACEs and females.

### PEER REVIEW

The peer review history for this article is available at https://publons.com/publon/10.1002/brb3.2748.

## FUNDING

This study is supported by National Natural Science Foundation of China (General Project), No. 81774366.

## IMPLICATIONS AND CONTRIBUTION

This study found that individuals exposed to any ACEs were more susceptible to ADHD than nonexposed individuals, and the negative effects of different forms of ACEs were nonequivalent, gender‐specific, and accumulative, which could provide critical evidence for understanding the association between ACEs and ADHD.

## CONFLICT OF INTEREST

There are no conflicts of interest arising from this research.

## Supporting information

APPENDIX 1: SEARCH STRATEGIES IN OVID MEDLINE(R)
APPENDIX 2: CHARACTERISTICS OF THE INCLUDED COHORT STUDIES
APPENDIX 3: CHARACTERISTICS OF THE INCLUDED CASE‐CONTROL STUDIES
APPENDIX 4: CHARACTERISTICS OF THE INCLUDED CROSS‐SECTION STUDIES
APPENDIX 5: REFERENCES OF INCLUDED STUDIES
APPENDIX 6: RESULT OF META‐ANALYSIS FOR THE ASSOCIATION BETWEEN ACES AND ADHD
APPENDIX 7: FOREST PLOTS AND FUNNEL PLOTS OF META‐ANALYSIS FOR THE ASSOCIATION BETWEEN 17 FORMS OF COMMON ACES AND ADHD
APPENDIX 8: RESULT OF META‐ANALYSIS FOR THE ASSOCIATION BETWEEN NUMBER OF ACES AND ADHD
APPENDIX 9: META‐ANALYSIS FOR THE ASSOCIATION BETWEEN ACES AND ADHD SYMPTOMS
APPENDIX 10: META‐ANALYSIS FOR THE ASSOCIATION BETWEEN ACES AND TYPE OF ADHD
APPENDIX 11: META‐REGRESSION FOR MODERATOR ANALYSIS
APPENDIX 12: STRATIFICATION ANALYSIS FOR POTENTIAL MODERATORS
APPENDIX 13: RESULTS OF TRIM AND FILL METHOD SENSITIVITY ANALYSIS

## Data Availability

The data of this study are available from the corresponding author, upon reasonable request.

## References

[brb32748-bib-0001] Bath, K. G. (2020). Synthesizing views to understand sex differences in response to early life adversity. Trends in Neurosciences, 43(5), 300–310. 10.1016/j.tins.2020.02.004 32353334 PMC7195459

[brb32748-bib-0002] Begg, C. B. , & Mazumdar, M. (1995). Operating characteristics of a rank correlation test for publication bias. Biometrics, 50, 1088–1101. 10.2307/2533446 7786990

[brb32748-bib-0003] Beran, T. N. , & Lupart, J. (2009). The relationship between school achievement and peer harassment in canadian adolescents the importance of mediating factors. School Psychology International, 30, 75–91. 10.1177/0143034308101851

[brb32748-bib-0004] Berens, A. E. , Jensen, S. K. G. , & Nelson, C. A. (2017). Biological embedding of childhood adversity: From physiological mechanisms to clinical implications. BMC Medicine, 15(1), 135–135. 10.1186/s12916-017-0895-4 28724431 PMC5518144

[brb32748-bib-0005] Borenstein, M. , Hedges, L. V. , Higgins, J. P. T. , & Rothstein, H. R. (2010). A basic introduction to fixed‐effect and random‐effects models for meta‐analysis. Research Synthesis Methods, 1(2), 97–111. 10.1002/jrsm.12 26061376

[brb32748-bib-0006] Brook, J. S. , Brook, D. W. , Zhang, C. , Seltzer, N. , & Finch, S. J. (2013). Adolescent ADHD and adult physical and mental health, work performance, and financial stress. Pediatrics, 131(1), 5–13. 10.1542/peds.2012-1725 23230074 PMC3529955

[brb32748-bib-0007] Calem, M. , Bromis, K. , Mcguire, P. , Morgan, C. , & Kempton, M. J. (2017). Meta‐analysis of associations between childhood adversity and hippocampus and amygdala volume in non‐clinical and general population samples. NeuroImage. Clinical, 14, 471–479. 10.1016/j.nicl.2017.02.016 28275547 PMC5331153

[brb32748-bib-0008] Chang, J. P.‐C. , Mondelli, V. , Satyanarayanan, S. K. , Chiang, Y.‐J. , Chen, H.‐T. , Su, K.‐P. , & Pariante, C. M. (2020). Cortisol, inflammatory biomarkers and neurotrophins in children and adolescents with attention deficit hyperactivity disorder (ADHD) in Taiwan. Brain, Behavior, and Immunity, 88, 105–113. 10.1016/j.bbi.2020.05.017 32418647

[brb32748-bib-0009] Colman, I. , Kingsbury, M. , Garad, Y. , Zeng, Y. , Naicker, K. , Patten, S. , Jones, P. B. , Wild, T. C. , & Thompson, A. H. (2015). Consistency in adult reporting of adverse childhood experiences. Psychological Medicine, 46(3), 543–549. 10.1017/S0033291715002032 26511669

[brb32748-bib-0010] Dahoun, T. , Nour, M. M. , Mccutcheon, R. A. , Adams, R. A. , Bloomfield, M. A. P. , & Howes, O. D. (2019). The relationship between childhood trauma, dopamine release and dexamphetamine‐induced positive psychotic symptoms: A [(11)C]‐(+)‐PHNO PET study. Translational Psychiatry, 9(1), 287–299. 10.1038/s41398-019-0627-y 31712556 PMC6848217

[brb32748-bib-0011] Danielson, M. L. , Bitsko, R. H. , Ghandour, R. M. , Holbrook, J. R. , Kogan, M. D. , & Blumberg, S. J. (2018). Prevalence of parent‐reported ADHD diagnosis and associated treatment among U.S. children and adolescents, 2016. Journal of Clinical Child and Adolescent Psychology: The Official Journal for the Society of Clinical Child and Adolescent Psychology, American Psychological Association, Division, 47(2), 199–212. 10.1080/15374416.2017.1417860 PMC5834391 29363986

[brb32748-bib-0012] Del Campo, N. , Chamberlain, S. R. , Sahakian, B. J. , & Robbins, T. W. (2011). The roles of dopamine and noradrenaline in the pathophysiology and treatment of attention‐deficit/hyperactivity disorder. Biological Psychiatry, 69(12), e145–e157. 10.1016/j.biopsych.2011.02.036 21550021

[brb32748-bib-0013] Erskine, H. E. , Baxter, A. J. , Patton, G. , Moffitt, T. E. , Patel, V. , Whiteford, H. A. , & Scott, J. G. (2017). The global coverage of prevalence data for mental disorders in children and adolescents. Epidemiology and Psychiatric Sciences, 26(4), 395–402. 10.1017/S2045796015001158 26786507 PMC6998634

[brb32748-bib-0014] Erskine, H. E. , Ferrari, A. J. , Polanczyk, G. V. , Moffitt, T. E. , Murray, C. J. L. , Vos, T. , Whiteford, H. A. , & Scott, J. G. (2014). The global burden of conduct disorder and attention‐deficit/hyperactivity disorder in 2010. Journal of Child Psychology and Psychiatry, 55(4), 328–336. 10.1111/jcpp.12186 24447211

[brb32748-bib-0015] Faraone, S. V. , Asherson, P. , Banaschewski, T. , Biederman, J. , Buitelaar, J. K. , Ramos‐Quiroga, J. A. , Rohde, L. A. , Sonuga‐Barke, E. J. S. , Tannock, R. , & Franke, B. (2015). Attention‐deficit/hyperactivity disorder. Nature Reviews Disease Primers, 1(1), 15020–15042. 10.1038/nrdp.2015.20 27189265

[brb32748-bib-0016] Forster, M. , Gower, A. L. , Borowsky, I. W. , & Mcmorris, B. J. (2017). Associations between adverse childhood experiences, student‐teacher relationships, and non‐medical use of prescription medications among adolescents. Addictive Behaviors, 68, 30–34. 10.1016/j.addbeh.2017.01.004 28088740

[brb32748-bib-0017] González, R. A. , Vélez‐Pastrana, M. C. , McCrory, E. , Kallis, C. , Aguila, J. , Canino, G. , & Bird, H. (2019). Evidence of concurrent and prospective associations between early maltreatment and ADHD through childhood and adolescence. Social Psychiatry & Psychiatric Epidemiology, 54(6), 671–682. 10.1007/s00127-019-01659-0 30903235

[brb32748-bib-0018] Hughes, K. , Bellis, M. A. , Hardcastle, K. A. , Sethi, D. , Butchart, A. , Mikton, C. , Jones, L. , & Dunne, M. P. (2017). The effect of multiple adverse childhood experiences on health: A systematic review and meta‐analysis. The Lancet Public Health, 2(8), e356–e366. 10.1016/S2468-2667(17)30118-4 29253477

[brb32748-bib-0019] Humphreys, K. L. , Aguirre, V. P. , & Lee, S. S. (2012). Association of anxiety and ODD/CD in children with and without ADHD. Journal of Clinical Child and Adolescent Psychology: The Official Journal for the Society of Clinical Child and Adolescent Psychology, American Psychological Association, Division 53, 41(3), 370–377. 10.1080/15374416.2012.656557 PMC6613574 22420771

[brb32748-bib-0020] Jangmo, A. , Stalhandske, A. , Chang, Z. , Chen, Q. , Almqvist, C. , Feldman, I. , Bulik, C. M. , Lichtenstein, P. , D'Onofrio, B. , Kuja‐Halkola, R. , & Larsson, H. (2019). Attention‐deficit/hyperactivity disorder, school performance, and effect of medication. Journal of the American Academy of Child and Adolescent Psychiatry, 58(4), 423–432. 10.1016/j.jaac.2018.11.014 30768391 PMC6541488

[brb32748-bib-0021] Jones, C. M. , Merrick, M. T. , & Houry, D. E. (2020). Identifying and preventing adverse childhood experiences: Implications for clinical practice. JAMA, 323(1), 25–26. 10.1001/jama.2019.18499 31689343 PMC9173206

[brb32748-bib-0022] Juffer, F. , & Van Ijzendoorn, M. H. (2005). Behavior problems and mental health referrals of international adoptees: A meta‐analysis. JAMA, 293(20), 2501–2515. 10.1001/jama.293.20.2501 15914751

[brb32748-bib-0023] Katsuki, F. , & Constantinidis, C. (2013). Bottom‐up and top‐down attention: Different processes and overlapping neural systems. The Neuroscientist, 20(5), 509–521. 10.1177/1073858413514136 24362813

[brb32748-bib-0024] Keller, S. M. , Nowak, A. , & Roth, T. L. (2019). Female pups receive more maltreatment from stressed dams. Developmental Psychobiology, 61(6), 824–831. 10.1002/dev.21834 30810229 PMC6711830

[brb32748-bib-0025] Koss, K. J. , & Gunnar, M. R. (2018). Annual Research Review: Early adversity, the hypothalamic‐pituitary‐adrenocortical axis, and child psychopathology. Journal of Child Psychology and Psychiatry, and Allied Disciplines, 59(4), 327–346. 10.1111/jcpp.12784 28714126 PMC5771995

[brb32748-bib-0026] Lara, C. , Fayyad, J. , De Graaf, R. , Kessler, R. C. , Aguilar‐Gaxiola, S. , Angermeyer, M. , Demytteneare, K. , De Girolamo, G. , Haro, J. M. , Jin, R. , Karam, E. G. , Lapine, J.‐P. , Mora, M. E. M. , Ormel, J. , Posada‐Villa, J. , & Sampson, N. (2009). Childhood predictors of adult attention‐deficit/hyperactivity disorder: Results from the World Health Organization World Mental Health Survey Initiative. Biological Psychiatry, 65(1), 46–54. 10.1016/j.biopsych.2008.10.005 19006789 PMC2629074

[brb32748-bib-0027] Lin, L. (2018). Bias caused by sampling error in meta‐analysis with small sample sizes. Plos One, 13(9), e0204056–e0204056. 10.1371/journal.pone.0204056 30212588 PMC6136825

[brb32748-bib-0028] Liu, L. u. , Zhao, Q. , Yu, X. , Xu, D. , Li, H. , Ji, N. , Wu, Z. , Cheng, J. , Su, Y. , Cao, Q. , Sun, L. , Qian, Q. , & Wang, Y. (2020). Monoaminergic genetic variants, prefrontal cortex–amygdala circuit, and emotional symptoms in children with ADHD: Exploration based on the gene–brain–behavior relationship. Journal of Attention Disorders, 25(9), 1272–1283. 10.1177/1087054719897838 31910717

[brb32748-bib-0029] Lugo‐Candelas, C. , Corbeil, T. , Wall, M. , Posner, J. , Bird, H. , Canino, G. , Fisher, P. W. , Suglia, S. F. , & Duarte, C. S. (2021). ADHD and risk for subsequent adverse childhood experiences: Understanding the cycle of adversity. Journal of Child Psychology and Psychiatry, and Allied Disciplines, 62(8), 971–978. 10.1111/jcpp.13352 33289088 PMC8169708

[brb32748-bib-0030] Maguire, S. A. , Williams, B. , Naughton, A. M. , Cowley, L. E. , Tempest, V. , Mann, M. K. , Teague, M. , & Kemp, A. M. (2015). A systematic review of the emotional, behavioural and cognitive features exhibited by school‐aged children experiencing neglect or emotional abuse. Child: Care, Health and Development, 41(5), 641–653. 10.1111/cch.12227 25733080

[brb32748-bib-0031] Malvaso, C. G. , Cale, J. , Whitten, T. , Day, A. , Singh, S. , Hackett, L. , Delfabbro, P. H. , & Ross, S. (2021). Associations between adverse childhood experiences and trauma among young people who offend: A systematic literature review. Trauma, Violence, & Abuse, 15248380211013132. Online ahead of print. 10.1177/15248380211013132 33960233

[brb32748-bib-0032] Mannuzza, S. , Klein, R. G. , Klein, D. F. , Bessler, A. , & Shrout, P. (2002). Accuracy of adult recall of childhood attention deficit hyperactivity disorder. American Journal of Psychiatry, 159(11), 1882–1888. 10.1176/appi.ajp.159.11.1882 12411223

[brb32748-bib-0033] Mclaughlin, K. A. , Weissman, D. , & Bitran, D. (2019). Childhood adversity and neural development: A systematic review. Annual Review of Developmental Psychology, 1, 277–312. 10.1146/annurev-devpsych-121318-084950 PMC7243625 32455344

[brb32748-bib-0034] Mcnutt, L.‐A. (2003). Estimating the relative risk in cohort studies and clinical trials of common outcomes. American Journal of Epidemiology, 157(10), 940–943. 10.1093/aje/kwg074 12746247

[brb32748-bib-0035] Mitchison, G. M. , & Njardvik, U. (2015). Prevalence and gender differences of ODD, anxiety, and depression in a sample of children with ADHD. Journal of Attention Disorders, 23(11), 1339–1345. 10.1177/1087054715608442 26443719

[brb32748-bib-0036] Moher, D. , Liberati, A. , Tetzlaff, J. , & Altman, D. G. (2009). Preferred reporting items for systematic reviews and meta‐analyses: The PRISMA statement. BMJ (Clinical Research Ed.), 339, b2535–b2535. 10.1136/bmj.b2535 PMC2714657 19622551

[brb32748-bib-0037] Nikolakopoulou, A. , Mavridis, D. , & Salanti, G. (2014). Demystifying fixed and random effects meta‐analysis. Evidence Based Mental Health, 17(2), 53–57. 10.1136/eb-2014-101795 24692250 PMC13404554

[brb32748-bib-0038] Orsini, N. , Li, R. , Wolk, A. , Khudyakov, P. , & Spiegelman, D. (2012). Meta‐analysis for linear and nonlinear dose‐response relations: Examples, an evaluation of approximations, and software. American Journal of Epidemiology, 175(1), 66–73. 10.1093/aje/kwr265 22135359 PMC3244608

[brb32748-bib-0039] Ouyang, L. , Fang, X. , Mercy, J. , Perou, R. , & Grosse, S. D. (2008). Attention‐deficit/hyperactivity disorder symptoms and child maltreatment: A population‐based study. The Journal of Pediatrics, 153(6), 851–856. 10.1016/j.jpeds.2008.06.002 18619612

[brb32748-bib-0040] Posner, J. , Polanczyk, G. V. , & Sonuga‐Barke, E. (2020). Attention‐deficit hyperactivity disorder. Lancet, 395(10222), 450–462. 10.1016/S0140-6736(19)33004-1 31982036 PMC7880081

[brb32748-bib-0041] Qu, G.‐B. , Wu, W. , Wang, L.‐L. , Tang, X. , Sun, Y.‐H. , Li, J. , & Wang, J. (2018). Systematic review and meta‐analysis found higher levels of behavioural problems in male left‐behind children aged 6–11 years. Acta Paediatrica, 107(8), 1327–1334. 10.1111/apa.14199 29280182

[brb32748-bib-0042] Retz, W. , Ginsberg, Y. , Turner, D. , Barra, S. , Retz‐Junginger, P. , Larsson, H. , & Asherson, P. (2021). Attention‐deficit/hyperactivity disorder (ADHD), antisociality and delinquent behavior over the lifespan. Neuroscience & Biobehavioral Reviews, 120, 236–248. 10.1016/j.neubiorev.2020.11.025 33271164

[brb32748-bib-0043] Rose, A. K. , Shaw, S. G. , Prendergast, M. A. , & Little, H. J. (2010). The importance of glucocorticoids in alcohol dependence and neurotoxicity. Alcoholism, Clinical and Experimental Research, 34(12), 2011–2018. 10.1111/j.1530-0277.2010.01298.x 21087289 PMC3058879

[brb32748-bib-0044] Schoeler, T. , Choi, S. W. , Dudbridge, F. , Baldwin, J. , Duncan, L. , Cecil, C. M. , Walton, E. , Viding, E. , Mccrory, E. , & Pingault, J.‐B. (2019). Multi‐polygenic score approach to identifying individual vulnerabilities associated with the risk of exposure to bullying. JAMA Psychiatry, 76(7), 730–738. 10.1001/jamapsychiatry.2019.0310 30942833 PMC6583782

[brb32748-bib-0045] Simon, V. , Czobor, P. , Balint, S. , Maszaros, A. , & Bitter, I. (2018). Prevalence and correlates of adult attention‐deficit hyperactivity disorder: Meta‐analysis. British Journal of Psychiatry, 194(3), 204–211. 10.1192/bjp.bp.107.048827 19252145

[brb32748-bib-0046] Stroup, D. F. , Stroup, D. F. , Berlin, J. A. , Morton, S. C. , Olkin, I. , Williamson, G. D. , Rennie, D. , Moher, D. , Becker, B. J. , Sipe, T. A. , & Thacker, S. B. (2000). Meta‐analysis of observational studies in epidemiologyA proposal for reporting. JAMA, 283(15), 2008–2012. 10.1001/jama.283.15.2008 10789670

[brb32748-bib-0047] Suglia, S. F. , Koenen, K. C. , Boynton‐Jarrett, R. E. , Chan, P. S. , Clark, C. J. , Danese, A. , Faith, M. S. , Goldstein, B. I. , Hayman, L. L. , Isasi, C. R. , Pratt, C. A. , Slopen, N. , Sumner, J. A. , Turer, A. , Turer, C. B. , & Zachariah, J. P. (2018). Childhood and adolescent adversity and cardiometabolic outcomes: A scientific statement from the american heart association. Circulation, 137(5), e15–e28. 10.1161/CIR.0000000000000536 29254928 PMC7792566

[brb32748-bib-0048] Tipton, E. , Hallberg, K. , Hedges, L. V. , & Chan, W. (2016). Implications of small samples for generalization: Adjustments and rules of thumb. Evaluation Review, 41(5), 472–505. 10.1177/0193841x16655665 27402612

[brb32748-bib-0049] Tang, S. , Jones, C. M. , Wisdom, A. , Lin, H.‐C. , Bacon, S. , & Houry, D. (2021). Adverse childhood experiences and stimulant use disorders among adults in the United States. Psychiatry Research, 299, 113870–113870. 10.1016/j.psychres.2021.113870 33780857 PMC8211100

[brb32748-bib-0050] Vaa, T. (2014). ADHD and relative risk of accidents in road traffic: A meta‐analysis. Accident Analysis & Prevention, 62, 415–425. 10.1016/j.aap.2013.10.003 24238842

[brb32748-bib-0051] Vachon, D. D. , Krueger, R. F. , Rogosch, F. A. , & Cicchetti, D. (2015). Assessment of the harmful psychiatric and behavioral effects of different forms of child maltreatment. JAMA Psychiatry, 72(11), 1135–1142. 10.1001/jamapsychiatry.2015.1792 26465073 PMC4699442

[brb32748-bib-0052] Vanderweele, T. J. (2020). Optimal approximate conversions of odds ratios and hazard ratios to risk ratios. Biometrics, 76(3), 746–752. 10.1111/biom.13197 31808145

[brb32748-bib-0053] Wan, Y. , Chen, R. , Wang, S. , Clifford, A. , Zhang, S. , Orton, S. , & Fangbiao, T. (2020). Associations of coping styles with nonsuicidal self‐injury in adolescents: Do they vary with gender and adverse childhood experiences? Child Abuse & Neglect, 104, 104470. 10.1016/j.chiabu.2020.104470 32234639

[brb32748-bib-0054] Wu, W. , Qu, G. , Wang, L. , Tang, X. , & Sun, Y. e‐H. (2019). Meta‐analysis of the mental health status of left‐behind children in China. Journal of Paediatrics and Child Health, 55(3), 260–270. 10.1111/jpc.14349 30604503

[brb32748-bib-0055] Zwicker, A. , Mackenzie, L. E. , Drobinin, V. , Bagher, A. M. , Howes Vallis, E. , Propper, L. , Bagnell, A. , Abidi, S. , Pavlova, B. , Alda, M. , Denovan‐Wright, E. M. , & Uher, R. (2020). Neurodevelopmental and genetic determinants of exposure to adversity among youth at risk for mental illness. Journal of Child Psychology and Psychiatry, 61(5), 536–544. 10.1111/jcpp.13159 31749149

