# Supplementary material for: Understanding the association between adverse childhood experiences and subsequent attention deficit hyperactivity disorder: A systematic review and meta‐analysis of observational studies
Source: Brain Behav. 2022 Sep 6;12(10):e32748. doi: 10.1002/brb3.2748 (PMC9575611; doi:10.1002/brb3.2748)
Supplement: Supplementary file 1 — APPENDIX 1: SEARCH STRATEGIES IN OVID MEDLINE(R) APPENDIX 2: CHARACTERISTICS OF THE INCLUDED COHORT STUDIES APPENDIX 3: CHARACTERISTICS OF THE INCLUDED CASE‐CONTROL STUDIES APPENDIX 4: CHARACTERISTICS OF THE INCLUDED CROSS‐SECTION STUDIES APPENDIX 5: REFERENCES OF INCLUDED STUDIES APPENDIX 6: RESULT OF META‐ANALYSIS FOR THE ASSOCIATION BETWEEN ACES AND ADHD APPENDIX 7: FOREST PLOTS AND FUNNEL PLOTS OF META‐ANALYSIS FOR THE ASSOCIATION BETWEEN 17 FORMS OF COMMON ACES AND ADHD APPENDIX 8: RESULT OF META‐ANALYSIS FOR THE ASSOCIATION BETWEEN NUMBER OF ACES AND ADHD APPENDIX 9: META‐ANALYSIS FOR THE ASSOCIATION BETWEEN ACES AND ADHD SYMPTOMS APPENDIX 10: META‐ANALYSIS FOR THE ASSOCIATION BETWEEN ACES AND TYPE OF ADHD APPENDIX 11: META‐REGRESSION FOR MODERATOR ANALYSIS APPENDIX 12: STRATIFICATION ANALYSIS FOR POTENTIAL MODERATORS APPENDIX 13: RESULTS OF TRIM AND FILL METHOD SENSITIVITY ANALYSIS [file BRB3-12-e32748-s001.docx]

**Understanding the Association Between Adverse Childhood Experiences and Attention Deficit Hyperactivity Disorder: A Systematic Review and Meta-Analysis of Observational Studies**

Ning Zhang MD^a^, Man Gao MM^b^, Jinglong Yu MD^a^, Qiang Zhang MM^a^, Weiguang Wang MD^c^, Congxiao Zhou MD^a^, Lingjia Liu MM^a^, Ting Sun MD^a^, Xing Liao PhD^d*^, Junhong Wang MD^a*^

^a^ Department of Pediatrics, Dongzhimen Hospital, Beijing University of Chinese Medicine, Beijing, China

^b^ Institute of Information on Traditional Chinese Medicine, China Academy of Chinese Medical Sciences, Beijing, China

^c^ School of Traditional Chinese Medicine, Beijing University of Chinese Medicine, Beijing, China

^d^ Center of Evidence Based Traditional Chinese Medicine, Institute of Basic Research in Clinical Medicine, China Academy of Chinese Medical Sciences, Beijing, China

**Appendices**

| **Appendix 1** | **Search Strategies in Ovid MEDLINE(R)** |  | **P2-3** |
| --- | --- | --- | --- |
| **Appendix 2** | **Characteristics of the Included Cohort Studies** |  | **P4-9** |
| **Appendix 3** | **Characteristics of the Included Case-Control Studies** |  | **P10-11** |
| **Appendix 4** | **Characteristics of the Included Cross-Section Studies** |  | **P12-20** |
| **Appendix 5** | **References of Included Studies** |  | **P21-25** |
| **Appendix 6** | **Result of Meta-Analysis for the Association Between ACEs and ADHD** |  | **P26** |
| **Appendix 7** | **Forest Plots and Funnel Plots of Meta-Analysis for the Association Between 17 Forms of Common ACEs and ADHD** |  | **P27-43** |
| **Appendix 8** | **Result of Meta-Analysis for the Association Between Number of ACEs and ADHD** |  | **P44-45** |
| **Appendix 9** | **Meta-Analysis for the Association Between ACEs and ADHD symptoms** |  | **P46-48** |
| **Appendix 10** | **Meta-Analysis for the Association Between ACEs and type of ADHD** |  | **P49-51** |
| **Appendix 11** | **Meta-Regression for Moderator Analysis** |  | **P52-54** |
| **Appendix 12** | **Stratification Analysis for Potential Moderators** |  | **P55-58** |
| **Appendix 13** | **Results of Trim and Fill Method Sensitivity Analysis** |  | **P59** |

**Appendix 1: Search Strategies in Ovid MEDLINE(R)**

1. Adverse Childhood Experiences/
2. ACEs.mp.
3. (adverse adj2 child* adj2 (experience* or event*)).ti.ab.kw.
4. 1 or 2 or 3
5. child abuse, sexual/ or human trafficking/ or rape/ or physical abuse/ or exp bullying/ or cyberbullying/ or exp bereavement/ or exp Sex Offenses/ or divorce/ or family conflict/ or maternal deprivation/ or paternal deprivation/ or single-parent family/ or family separation/ or adoption/ or mass casualty incidents/ or exp natural disasters/ or mass casualty incidents/ or exp natural disasters/ or exp poverty/
6. (rape or bullying or cyberbullying or bereavement or neglect or "natural disaster*" or "paternal deprivation" or single-parent or abuse* or traffick* or adopt* or poverty).ti.ab.kw.
7. ((household or parent* or maternal or mother or paternal or father or familial) adj3 (mental disorder* or mental illness or psychos?s or psychiatric disorder*)).ti.ab.kw.
8. 5 or 6 or 7
9. adolescent/ or child/ or child, preschool/ or infant/ or infant, newborn/
10. Early Life.mp.
11. (infant* or child* or juvenile* or teen* or adolescen* or young* or girl? or boy? or school* or pre-school* or preschool* or nurser* or playschool* or kindergarten* or prekindergarten* or pupil* or youth* or "young adult*" or "early adult*" or minor*).ti.ab.kw.
12. 9 or 10 or 11
13. 8 and 12
14. 4 or 13
15. exp "attention deficit and disruptive behavior disorders"/ or attention deficit disorder with hyperactivity/ or conduct disorder/
16. (ADHD or ADDH or ADHS).ti.ab.kw.
17. ((attention* or behav*) adj3 (defic* or dysfunc* or disorder*)).ti.ab.kw.
18. ((disrupt* or defian*) adj3 (disorder* or behav*)).ti.ab.kw.
19. (impulsiv* or inattentiv* or inattention*).ti.ab.kw.
20. Hyperkinesis/
21. (hyperkin* or hyperactiv* or hyper-activ* or HKD or TDAH).ti.ab.kw.
22. 15 or 16 or 17 or 18 or 19 or 20 or 21
23. Epidemiologic Research Design/ or Epidemiologic Methods/ or Epidemiologic Studies/ or epidemiologic study characteristics/
24. observational studies as topic/ or Observational Study/
25. Cohort Studies/ or case-control studies/ or cross-sectional studies/
26. longitudinal studies/ or prospective studies/ or retrospective studies/
27. (Incidence or Prevalence or epidemiolog* or case-control or Case-Base or Case-Referrent or Case-Comparison or cohort or cross-sectional or Prospective or Retrospective or Longitudinal).ti.ab.kw.
28. 23 or 24 or 25 or 26 or 27
29. 13 and 22 and 28
30. Animals/
31. Humans/
32. 30 and 31
33. 30 not 32
34. 29 not 33

Updated in July 2021

| **Appendix 2: Characteristics of the Included Cohort Studies** | | | | | | | | | | |
| --- | --- | --- | --- | --- | --- | --- | --- | --- | --- | --- |
| Study | Setting | Study Design | Sample Size | Exposure | Measures of ACEs | Diagnosis | Variables adjusted for | Quality Assessment | | |
|  |  |  |  |  |  |  |  | Selection | Comparability | Outcome |
| Russell 2014(1) | UK | prospective | 13,305 | poverty,  single parent | interview-based | diagnostic interview-based | SDQ hyperactivity & impact subscales | **🟑🟑🟑🟑** | **🟑** | **🟑🟑🟑** |
| Björkenstam 2016(2) | Sweden | prospective | 107,704 | familial death, parental substance misuse, parental psychiatric disorder, severe parental criminality, parental separation, living on public assistance, residential instability | record-based | record-based | sex, birth year, mother's country of birth, parental income and education, own psychopathology before age 15 | **🟑🟑🟑🟑** | **🟑🟑** | **🟑🟑** |
| Stern 2018(3) | UK | prospective | 1,116 | abuse/neglect, bullying by peers, domestic violence | questionnaire-based | **Childhood ADHD:**  mother / teacher- report Young adult ADHD: diagnostic interview-based | sex, IQ, SES, mother’s depression | **🟑🟑🟑** | **🟑** | **🟑🟑** |
| Malone 2002(4) | USA | prospective | **11-year old:** 1035 17-year old: 1076 | paternal alcohol dependence | interview-based | diagnostic interview-based | paternal maximum consumption | **🟑🟑🟑** | **🟑** | **🟑🟑** |
| Björkenstam 2018(5) | Sweden | prospective | 543,650 | familial death, parental substance misuse, parental psychiatric disorder, severe parental criminality, parental separation, household living on public assistance, residential instability | record-based | record-based | birth year, sex, mother’s country of birth, highest parental educational attainment, place of residence, all childhood adversities simultaneously | **🟑🟑🟑🟑** | **🟑🟑** | **🟑🟑** |
| Feder 2009(6) | USA | retrospective | 58 | maternal depression | interview-based | diagnostic interview-based | child age, gender, any possible correlation between multiple children from one family | **🟑🟑🟑** | **🟑🟑** | **🟑🟑** |
| Ouyang 2008(7) | USA | prospective | 14,322 | supervision neglect, physical neglect, physical abuse, sexual abuse | interview-based | questionnaire-based | race, sex, age cohort, family structure, whether the mother was a teenager when the child was born, whether the biological father was ever jailed, parent education level, family size adjusted poverty status | **🟑🟑🟑** | **🟑🟑** | **🟑🟑** |
| Gifford 2019(8) | USA | prospective | 1,420 | parental incarceration | interview-based | diagnostic interview-based | sex, race/ethnicity | **🟑🟑🟑** | **🟑** | **🟑🟑🟑** |
| Day 2016(9) | Canada | prospective | 275 | peer victimization | questionnaire-based | diagnostic interview-based | participant gender, familial socioeconomic status, family functioning, childhood emotional and behavioral problems | **🟑🟑🟑** | **🟑** | **🟑🟑🟑** |
| Hunt 2017(10) | USA | prospective | 3,108 | physical neglect, physical abuse, emotional abuse, emotional neglect, parental Substance Abuse, parental Incarceration, parental Anxiety and/or depression, parental domestic violence | interview-based | self-report | birth, sociodemographic characteristics | **🟑🟑🟑🟑** | **🟑🟑** | **🟑🟑** |
| Kasen 1996(11) | USA | prospective | 648 | single custodial mother, stepfamily | interview-based | diagnostic interview-based | age, socioeconomic status, immaturity, anxiety, and affective problems in 1975, pooled sample, sex | **🟑🟑🟑** | **🟑🟑** | **🟑🟑🟑** |
| Choi 2016(12) | Korea | prospective | 2,159 | single-parent family, low income | questionnaire-based | questionnaire-based | age, gender, residential area, monthly household income, parental marital status, family history of psychiatric disorders, preterm birth, birth weight | **🟑🟑🟑** | **🟑🟑** | **🟑🟑🟑** |
| González 2019(13) | USA | prospective | 2,176 | neglect,  physical abuse, emotional abuse, sexual abuse, foster placement | scale / question-based | diagnostic interview-based | study site, wave, age, gender, household education and income band, any parental psychopathology, having taken ADHD medication | **🟑🟑** | **🟑🟑** | **🟑🟑** |
| Ebejer 2012(14) | Australia | retrospective | 3,795 | sexual assault, conflict with parents, parental arguing, | interview-based | diagnostic interview-based | gender, education | **🟑🟑🟑** | **🟑** | **🟑🟑🟑** |
| Sciberras 2011(15) | Australia | prospective | 3,474 | maternal post-natal depression | questionnaire-based | diagnostic interview-based | gender, primary caregiver education, biological father in the home, maternal smoking during pregnancy | **🟑🟑** | **🟑🟑** | **🟑** |
| Heiervang 2007(16) | Norway | retrospective | 996 | nontraditional family type, poverty | interview-based | diagnostic interview-based | gender, learning difficulties, paternal education, comorbidity, family type, household income | **🟑🟑🟑** | **🟑** | **🟑** |
| Østergaard 2016(17) | Denmark | prospective | 994,407 | low social class, severe marital discord, paternal criminality, maternal mental disorder, placement in out-of-home care | record-based | record-based | calendar year | **🟑🟑🟑🟑** | **🟑🟑** | **🟑🟑🟑** |
| Nomura 2012  (18) | USA | prospective | 212 | low socioeconomic status | interview-based | diagnostic interview-based | ages of the mother and child, sex, race/ethnicity, and LBW, self-reports of parental ADHD symptoms, derived from the Conners’ Adult ADHD Rating Scale, maternal alcohol use, and smoking during pregnancy, the risk-group status | **🟑🟑🟑** | **🟑🟑** | **🟑🟑🟑** |
| Keyes 2008(19) | USA | retrospective | 1,232 | adopted in infancy | record-based | diagnostic interview-based | age at assessment, gender, parental SES | **🟑🟑🟑** | **🟑🟑** | **🟑🟑🟑** |
| Sagiv 2013(20) | USA | prospective | 461 | household income, maternal marital status | questionnaire / inventory-based | record-based | parental characteristics, maternal education at child's school age, paternal education at child's school age, annual household income at child's school age, maternal smoking during pregnancy, maternal alcohol consumption during pregnancy, maternal illicit drug use in year prior to birth, maternal IQ, maternal depression symptoms, mhild's characteristics, race/ethnicity, type of school, number of siblings living in house | **🟑🟑🟑** | **🟑🟑** | **🟑🟑** |
| Lee 2013(21) | USA | prospective | 12,136 - 14,523 | parental incarceration | interview-based | diagnostic interview-based | race, grade, gender, family structure, foreign born, father and mother education, father and mother alcoholism, maltreatment , family receipt of public assistance | **🟑🟑🟑** | **🟑🟑** | **🟑🟑** |
| Marmorstein 2009(22) | USA | retrospective | 1,252 | parental alcohol or illicit drug dependence | interview-based | diagnostic interview-based | parental drug dependence, parental alcohol dependence | **🟑🟑🟑** | **🟑** | **🟑🟑** |
| McCoy 2014(23) | Sweden | prospective | 870,017 | parental severe mental illness | record-based | record-based | offspring sex, parity, SMI in the other parent, coded parental country of origin as Sweden or not Sweden, gestational age, birth weight, small for gestational age. | **🟑🟑🟑** | **🟑🟑** | **🟑🟑** |
| Choi 2017(24)) | Korea | prospective | 18,029 | low socioeconomic status | record-based | record-based | sex, region, co-morbidity of ADHD | **🟑🟑🟑🟑** | **🟑** | **🟑🟑🟑** |
| Assari 2019(25) | USA | prospective | 2,006 | low socioeconomic status | mother-report | parent / guardian-report | child gender, family structure, maternal age | **🟑** | **🟑** | **🟑🟑** |
| Jimenez 2017(26) | USA | prospective | 1,572 | child maltreatment, maternal depressive symptoms, parental Substance use, caregiver treated violently, parental incarceration | record-based | mother-report | child’s gender, the mother’s race, ethnicity, the mother’s education level, the relationship status of the parents at the time of child’s birth, household income at the time of the 9-year follow-up interview | **🟑🟑🟑** | **🟑** | **🟑🟑🟑** |
| Guhn 2020(27) | Canada | prospective | 89,404 | divorced, separated, widowed, low socioeconomic status | record-based | record-based | sex, age, SES, maternal, birthweight, parental marital status, mother’s birthplace | **🟑🟑🟑** | **🟑🟑** | **🟑🟑** |
| Chen 2020(28) | China | prospective | 708,515 | parental depression | questionare-based | diagnostic interview-based | income level, residence, parental ages, sex of children | **🟑🟑🟑🟑** | **🟑** | **🟑🟑🟑** |

Abbreviations: SDQ, the Strengths and Difficulties Questionnaire; IQ, intelligence quotient; SES, low socioeconomic status; LBW, low birth weight; SMI, severe mental illness.

*Note*: The allocation of stars represents the quality of including studies, each star stands for one point within the selection, comparability and outcome categories.

| **Appendix 3:** **Characteristics of the Included Case-Control Studies** | | | | | | | | | | |
| --- | --- | --- | --- | --- | --- | --- | --- | --- | --- | --- |
| Study | Setting | Study Design | Sample Size | Exposure | Measures of ACEs | Diagnosis | Variables adjusted for | Quality Assessment | | |
|  |  |  |  |  |  |  |  | Selection | Comparability | Outcome |
| Bejerot 2013(29) | Sweden | retrospective | 210 | peer victimization | interview-based | scale-based | sex, diagnosis, previous child psychiatric treatment, educational level | **🟑🟑** | **🟑** | **🟑** |
| Pheula 2011(30) | Brazil | retrospective | 200 | marital discord,  lower levels of FRI | scale / related index-based | diagnostic interview-based | age, gender, ethnicity, schooling, maternal ADHD, IQ, maternal smoking during pregnancy, comorbidities | **🟑🟑🟑** | **🟑🟑** | **🟑🟑** |
| Isaksson 2013(31) | Sweden | retrospective | 418 | childhood adversity | questionare / inventory-based | scale-based | age, cortisol level at awakening, sex | **🟑🟑🟑** | **🟑🟑** | **🟑** |
| du Prel Carroll 2012(32) | China | retrospective | 322 | single/step parent, family conflicts, emotional abuse, accidental injury | scale-based, parent-report | diagnostic interview-based | sex, age, maternal age at childbirth, maternal stress during pregnancy, pregnancy induced hypertension, maternal education, paternal education, single child, family structure, family conflicts, emotional abuse, domestic tobacco smoke, domestic alcohol consumption, physical activity, TV viewing, internet usage, accidental injury, dietary supplement intake | **🟑🟑🟑** | **🟑🟑** | **🟑** |
| Ford 1999(33) | USA | retrospective | 165 | accident/injury/illness trauma, victimization trauma | questionare / inventory-based | questionare-based | age, gender,bseverity of internalizing behavior problems, social competence, family psychopathology, parentchild relationship quality | **🟑🟑** | **🟑🟑** | **🟑🟑** |
| Carballo 2013(34) | Spain | retrospective | 16,823 | single parent, other family/institution, large sibship size | record-based | record-based | sibship size, maternal and paternal age at birth, maternal and paternal educational attainment, family structure, patient’s sex. | **🟑🟑🟑** | **🟑🟑** | **🟑🟑** |
| Joelsson 2017(35) | Finland | retrospective | 48,765 | parental psychopathology | record-based | record-based | maternal age, maternal socioeconomic status, paternal age, maternal smoking during pregnancy | **🟑🟑🟑🟑** | **🟑** | **🟑🟑** |
| Park 2014(36) | Korea | retrospective | 900 | postpartum depression | self-report, scale-based | diagnostic interview-based | primary caretaker other than mother, change in primary care taker | **🟑🟑🟑🟑** | **🟑** | **🟑🟑** |

*Note*: The allocation of stars represents the quality of including studies, each star stands for one point within the selection, comparability and outcome categories.

| **Appendix 4:** **Characteristics of the Included Cross-Section Studies** | | | | | | | | | | |
| --- | --- | --- | --- | --- | --- | --- | --- | --- | --- | --- |
| Study | Setting | Study Design | Sample Size | Exposure | Measures of ACEs | Diagnosis | Variables adjusted for | Quality Assessment | | |
|  |  |  |  |  |  |  |  | Selection | Comparability | Outcome |
| Mhalla 2018(37) | Tunisia | retrospective | 447 | family psychiatric disorders history , family somatic disorders history, family history of addiction, bad relationships with parents, maltreatment history | questionnaire-based | scale-based | parental situation, relation with parents, relation with peers, psychiatric family history, personal somatic history, personal psychiatric history, smoking, alcohol use, cannabis use, excessive Internet use, excessive television use, excessive video game use, maltreatment history, school results, sleep disorders | **🟑🟑🟑** | **🟑** | **🟑🟑🟑** |
| Fuller-Thomson 2014(38) | Canada | retrospective | 13,054 | parental divorce, parental addiction, physically abused as a child | interview-based | self-report | age, race, gender, 3 other types of adverse childhood experiences | **🟑🟑** | **🟑🟑** | **🟑** |
| Fuller-Thomson 2015(39) | Canada | retrospective | 23,373 | parental domestic violence, childhood physical abuse, sexual abuse | interview-based | self-report | age, race, gender. | **🟑🟑** | **🟑🟑** | **🟑** |
| Konstenius 2017(40) | Australia, Belgium, France, Hungary, Netherlands, Norway, Spain, Sweden, Switzerl and,USA | retrospective | 1,148 | sexual abuse, physical abuse, emotional abuse, violence in the family, neglect | questionnaire-based | scale-based | age, sex | **🟑🟑** | **🟑🟑** | **🟑🟑🟑** |
| Rowland 2018(41) | USA | retrospective | 953 | low socioeconomic status | self-report | diagnostic interview-based | childhood symptom profile and severity as well as for country, age , gender in the sub-sample of respondents with a history of childhood ADHD to predict current adult ADHD | **🟑** | **🟑🟑** | **🟑🟑🟑** |
| Lara 2009(42) | Colombia, Mexico, USA, Lebanon, Belgium, France, Germany, Italy, Netherlands, Spain | retrospective | 11,422 | neglect, physical abuse, sexual abuse, parental death, parental divorce , paternal psychopathology, family violence, economic adversity | interview-based | diagnostic interview-based | age, sex, race-ethnicity, childhood ADHD symptom profiles and severity | **🟑🟑** | **🟑🟑** | **🟑🟑🟑** |
| Sugaya 2012(43) | USA | retrospective | 34,384 | child physical abuse | interview-based | diagnostic interview-based | sociodemographic characteristics and psychiatric comorbidities | **🟑🟑🟑** | **🟑🟑** | **🟑🟑🟑** |
| Kim 2009(44) | Korea | retrospective | 2,673 | maternal depression during pregnancy, parental marital discord, parental separation or divorce, primary caregiver who is not a biological parent, changes in caregiver | questionnaire-based | diagnostic interview-based | age, gender, socioeconomic status | **🟑** | **🟑🟑** | **🟑🟑🟑** |
| Islam 2020(45) | Australia | retrospective | 2,166 | traditional bullying, cyberbullying, both bullying | self-report | diagnostic interview-based | age, gender, remoteness, grade, household income, family type, parents' education, substance use by the child, the time spent on using internet and playing electronic games. | **🟑** | **🟑🟑** | **🟑🟑🟑** |
| Uddin 2020(46) | USA | retrospective | 44,684 | divorce/separation, parent's incarceration, domestic violence, mentally ill or suicidal in caregiver, alcohol or drug problem in caregiver, socioeconomic hardship, death of a parent, victim/witnessed neighborhood violence, experienced discrimination or unfair treatment | parent / guardian-report | self-report | child's age, sex, race, nativity, parental education, household income, FRCI, parental stress | **🟑** | **🟑🟑** | **🟑🟑🟑** |
| Brown 2017(47) | USA | retrospective | 76,227 | socioeconomic hardship, parental Divorce, familial substance abuse, familial mental illness, neighborhood violence, incarceration, domestic violence, discrimination | parent-report | parent-report | sex, age, race, insurance status | **🟑** | **🟑🟑** | **🟑** |
| Perales 2017(48) | Australia | retrospective | 6,310 | blended family, separation | interview-based | diagnostic interview-based | sex, age of child, household income | **🟑** | **🟑🟑** | **🟑🟑🟑** |
| Flisher 1997(49) | USA | retrospective | 665 | physical abuse | interview-based | diagnostic interview-based | gender, age. ethniciry, income. site, family environment, family psychiatric history, perinatal problems, current physical health, history of sexual abuse | **🟑** | **🟑🟑** | **🟑🟑🟑** |
| Lola 2019(50) | Ethiopia | retrospective | 1,238 | divorced/widowed, living with single parent, poverty | no information provided | scale-based | sex, family marital status, living circumstances, child birth order/rank, family size, wealth index | **🟑** | **🟑** | **🟑🟑🟑** |
| Sawyer 2018(51) | Australia | retrospective | 8,921 | single-parent, low family income | questionnaires-based | diagnostic interview-based | survey year, gender, age, family structure, family income | **🟑** | **🟑🟑** | **🟑🟑🟑** |
| Bomysoad 2020(52) | USA | retrospective | 29,617 | household substance abuse problems, parental mental illness, domestic violence, neighborhood violence, parental divorce, parental death, parental incarceration, race/ethnicity-based discrimination, economic hardship | no information provided | self-report | no information provided | **🟑** | **🟑** | **🟑** |
| Farahat 2014(53) | Egypt | retrospective | 1,362 | family history of psychiatric illness, family size, intact family, disrupted family structure | parent / teacher-report | questionnaire-based | neonatal problems, family history of psychiatric illness, sex, family history of medical illness, consanguinity, antenatal illness and drug use, family size > 4, Low maternal education, paternal smoking, unemployed father, low paternal education, caesarian section, disrupted families | **🟑🟑** | **🟑** | **🟑🟑🟑** |
| Walker 2021(54) | USA | retrospective | 40,075 | parental divorce, parent or guardian death, parent or guardian incarcerated, domestic violence, community violence, lived with mental illness, lived with substance abuse, racial discrimination, hard to get by on income | question-based | self-report | age in years, gender, birth weight, race-ethnicity, maternal age at birth, parental aggravation, household education, and poverty status | **🟑** | **🟑🟑** | **🟑🟑🟑** |
| Crouch 2021(55) | USA | retrospective | 42,068 | parental separation/divorce, parental death, household incarceration, witness household violence, witness neighborhood violence, household mental illness, household substance abuse, racial/ethnic mistreatment | question-based | self-report | sex of child, age of child, race/ethnicity of child, caregiver’s relation to the child, primary language spoken in the home, caregiver education, family structure, poverty/income level, health insurance type | **🟑🟑** | **🟑🟑** | **🟑🟑🟑** |
| Bielas 2016(56) | Switzerland | retrospective | 130 | emotional abuse, physical abuse, sexual abuse, emotional neglect, physical neglect, battered mother, parental separation or divorce, mental illness in household, household substance abuse, incarcerated household member | inventory-based | diagnostic interview-based | demographics, ACE total score, and irritability score, interaction term ACE total score * irritability score | **🟑🟑** | **🟑🟑** | **🟑🟑🟑** |
| Calthorpe 2021(57) | USA | retrospective | 23,668 | economic hardship, parental Divorce, death of parent/guardian, incarceration of parent/guardian, domestic violence, neighborhood violence, household Mental Illness, household Alcohol/Drug Problem, racial Discrimination | parent / guardian-report | parent / guardian-report | sex, race/ ethnicity, age, household income, parent education, special health care needs, primary language in the home, survey respondent relation to child, unemployment | **🟑🟑** | **🟑🟑** | **🟑** |
| Montes 2018(58) | USA | retrospective | 1,624 | food and housing insecurity, live with a parent who is separated or divorced, death of a parent, parental incarceration, witness of violence, household mental illness, household alcohol/drug problem, racial discrimination | no information provided | parent / guardian-report | sex, race/ ethnicity, age, household income, parent education, special health care needs, primary language in the home, survey respondent relation to child, unemployment | **🟑** | **🟑** | **🟑** |
| Machado 2020(59) | Portugal | retrospective | 101 | physical abuse,  sexual abuse, neglect,  death of parents or caregivers,  parental divorce, parental absence, residence in orphanages or foster families,  parental mental illness, exposure to violence,  economic difficulties, severe organic disease | interview-based | scale-based | social determinants of mental health, parent-reported ASD severity | **🟑** | **🟑** | **🟑🟑🟑** |
| Fulton 2015(60) | USA | retrospective | 58,943 | step family, single mother, poverty | no information provided | parent / guardian-report | diagnosis before the first NSCH wave, children diagnosed before age 5, the age for public school entry | **🟑🟑** | **🟑** | **🟑** |
| Bauermeister 2007(61) | USA | retrospective | 2,660 | poverty, caretaker depression, caretaker expulsion/arrested, maltreatment | interview-based, scale based | diagnostic interview-based | caretaker’s education, marital status, perception of poverty, and number of comorbid diagnoses, demographic, child, school, family variables | **🟑🟑** | **🟑🟑** | **🟑🟑🟑** |
| Ferrer 2017(62) | Spain | retrospective | 204 | physical abuse, emotional abuse, physical neglect, physical neglect | questionnaire-based | diagnostic interview-based | gender | **🟑** | **🟑** | **🟑🟑🟑** |
| Siddique 2011(63) | Canada | retrospective | 1,819 | low socioeconomic status | scale-based | diagnostic interview-based | gender, age, PM10, BMI | **🟑🟑** | **🟑🟑** | **🟑🟑🟑** |
| Lehmann 2013(64) | Norway | retrospective | 219 | violence exposure, serious neglect | interview-based | diagnostic interview-based | age, age at first placement, number of placements, serious neglect or violence exposure | **🟑🟑🟑** | **🟑** | **🟑🟑** |

Abbreviations: FRCI, family resilience and connection index; ASD, Autistic Spectrum Disorder; NSCH, National Survey of Children's Health; PM10, particulate matter with a diameter of less than 10 μm; BMI, Body Mass Index.

*Note*: The allocation of stars represents the quality of including studies, each star stands for one point within the selection, comparability and outcome categories.

**Appendix 5: References of Included Studies**

1. Russell G, Ford T, Rosenberg R, Kelly S. The association of attention deficit hyperactivity disorder with socioeconomic disadvantage: alternative explanations and evidence. J Child Psychol Psychiatry. 2014;55(5):436-445.

2. Björkenstam E, Burstrom B, Vinnerljung B, Kosidou K. Childhood adversity and psychiatric disorder in young adulthood: An analysis of 107,704 Swedes. J Psychiatr Res. 2016;77:67-75.

3. Stern A, Agnew-Blais J, Danese A, Fisher HL, Jaffee SR, Matthews T, et al. Associations between abuse/neglect and ADHD from childhood to young adulthood: A prospective nationally-representative twin study. Child Abuse & Neglect. 2018; 81: 274-285.

4. Malone SM, Iacono WG, McGue M. Drinks of the father: father's maximum number of drinks consumed predicts externalizing disorders, substance use, and substance use disorders in preadolescent and adolescent offspring. Alcoholism: Clinical & Experimental Research. 2002;26(12):1823-1832.

5. Björkenstam E, Björkenstam C, Jablonska B, Kosidou K. Cumulative exposure to childhood adversity, and treated attention deficit/hyperactivity disorder: a cohort study of 543 650 adolescents and young adults in Sweden. Psychological Medicine. 2018;48(3):498-507.

6. Feder A, Alonso A, Tang M, Liriano W, Warner V, Pilowsky D, et al. Children of low-income depressed mothers: psychiatric disorders and social adjustment. Depression & Anxiety (1091-4269). 2009;26(6):513-520.

7. Ouyang L, Fang X, Mercy J, Perou R, Grosse SD. Attention-deficit/hyperactivity disorder symptoms and child maltreatment: A population-based study. The Journal of Pediatrics. 2008; 153(6): 851-856.

8. Gifford EJ, Eldred Kozecke L, Golonka M, Hill SN, Costello EJ, Shanahan L, et al. Association of Parental Incarceration With Psychiatric and Functional Outcomes of Young Adults. JAMA Network Open. 2019;2(8):e1910005-e.

9. Day KL, Schmidt LA, Vaillancourt T, Saigal S, Boyle MH, Van Lieshout RJ. Long-term Psychiatric Impact of Peer Victimization in Adults Born at Extremely Low Birth Weight. Pediatrics. 2016;137(3):e20153383.

10. Hunt TKA, Slack KS, Berger LM. Adverse childhood experiences and behavioral problems in middle childhood. Child Abuse & Neglect. 2017;67:391-402..

11. Kasen S, Cohen P, Brook JS, Hartmark C. A multiple-risk interaction model: effects of temperament and divorce on psychiatric disorders in children. Journal of Abnormal Child Psychology. 1996;24(2):121-150.

12. Choi W-J, Kwon H-J, Lim MH, Lim J-A, Ha M. Blood lead, parental marital status and the risk of attention-deficit/hyperactivity disorder in elementary school children: A longitudinal study. Psychiatry Research. 2016; 236:42-46..

13. González RA, Vélez-Pastrana MC, McCrory E, Kallis C, Aguila J, Canino G, et al. Evidence of concurrent and prospective associations between early maltreatment and ADHD through childhood and adolescence. Social Psychiatry & Psychiatric Epidemiology. 2019;54(6):671-682.

14. Ebejer JL, Medland SE, van der Werf J, Gondro C, Henders AK, Lynskey M, et al. Attention deficit hyperactivity disorder in Australian adults: prevalence, persistence, conduct problems and disadvantage. PLoS ONE. 2012;7(10):e47404.

15. Sciberras E, Ukoumunne OC, Efron D. Predictors of Parent-Reported Attention-Deficit/Hyperactivity Disorder in Children Aged 6–7 years: A National Longitudinal Study. Journal of Abnormal Child Psychology. 2011;39(7):1025-1034.

16. Heiervang E, Stormark KM, Lundervold AJ, Heimann M, Goodman R, Posserud M-B, et al. Psychiatric Disorders in Norwegian 8- to 10-Year-Olds: An Epidemiological Survey of Prevalence, Risk Factors, and Service Use. Journal of the American Academy of Child & Adolescent Psychiatry. 2007;46(4):438-447.

17. Østergaard SD, Larsen JT, Dalsgaard S, Wilens TE, Mortensen PB, Agerbo E, et al. Predicting ADHD by Assessment of Rutter's Indicators of Adversity in Infancy. PLoS ONE. 2016;11(6):e0157352-e.

18. Nomura Y, Marks DJ, Grossman B, Yoon M, Loudon H, Stone J, et al. Exposure to gestational diabetes mellitus and low socioeconomic status: effects on neurocognitive development and risk of attention-deficit/hyperactivity disorder in offspring. Arch Pediatr Adolesc Med. 2012;166(4):337-343.

19. Keyes MA, Sharma A, Elkins IJ, Iacono WG, McGue M. The mental health of US adolescents adopted in infancy. Arch Pediatr Adolesc Med. 2008;162(5):419-425.

20. Sagiv SK, Epstein JN, Bellinger DC, Korrick SA. Pre- and postnatal risk factors for ADHD in a nonclinical pediatric population. Journal of attention disorders. 2013;17(1):47-57.

21. Lee RD, Fang X, Luo F. The impact of parental incarceration on the physical and mental health of young adults. Pediatrics. 2013;131(4):e1188-e95.

22. Marmorstein NR, Iacono WG, McGue M. Alcohol and illicit drug dependence among parents: associations with offspring externalizing disorders. Psychological medicine. 2009;39(1):149-155.

23. McCoy BM, Rickert ME, Class QA, Larsson H, Lichtenstein P, D'Onofrio BM. Mediators of the association between parental severe mental illness and offspring neurodevelopmental problems. Annals of epidemiology. 2014;24(9):629-34.e1.

24. Choi Y, Shin J, Cho KH, Park E-C. Change in household income and risk for attention deficit hyperactivity disorder during childhood: A nationwide population-based cohort study. J Epidemiol. 2017;27(2):56-62.

25. Assari S, Caldwell CH. Family Income at Birth and Risk of Attention Deficit Hyperactivity Disorder at Age 15: Racial Differences. Children (Basel). 2019;6(1):10-21.

26. Jimenez ME, Wade R, Jr., Schwartz-Soicher O, Lin Y, Reichman NE. Adverse Childhood Experiences and ADHD Diagnosis at Age 9 Years in a National Urban Sample. Academic pediatrics. 2017;17(4):356-361.

27. Guhn M, Emerson SD, Mahdaviani D, Gadermann AM. Associations of Birth Factors and Socio-Economic Status with Indicators of Early Emotional Development and Mental Health in Childhood: A Population-Based Linkage Study. Child Psychiatry & Human Development. 2020;51(1):80-93.

28. Chen L-C, Chen M-H, Hsu J-W, Huang K-L, Bai Y-M, Chen T-J, et al. Association of parental depression with offspring attention deficit hyperactivity disorder and autism spectrum disorder: A nationwide birth cohort study. Journal of Affective Disorders. 2020;277:109-114.

29. Bejerot S, Humble MB. Childhood clumsiness and peer victimization: a case-control study of psychiatric patients. BMC Psychiatry. 2013;13:68-79.

30. Pheula GF, Rohde LA, Schmitz M. Are family variables associated with ADHD, inattentive type? A case-control study in schools. European Child and Adolescent Psychiatry. 2011;20(3):137-145.

31. Isaksson J, Nilsson KW, Lindblad F. Early psychosocial adversity and cortisol levels in children with attention-deficit/hyperactivity disorder. European Child and Adolescent Psychiatry. 2013;22(7):425-432.

32. du Prel Carroll X, Yi H, Liang Y, Pang K, Leeper-Woodford S, Riccardi P, et al. Family-environmental factors associated with attention deficit hyperactivity disorder in Chinese children: a case-control study. PLoS ONE. 2012;7(11):e50543.

33. Ford JD, Racusin R, Daviss WB, Ellis CG, Thomas J, Rogers K, et al. Trauma exposure among children with oppositional defiant disorder and attention deficit–hyperactivity disorder. Journal of Consulting and Clinical Psychology. 1999;67(5):786-9.

34. Carballo JJ, García-Nieto R, Álvarez-García R, Caro-Cañizares I, López-Castromán J, Muñoz-Lorenzo L, et al. Sibship size, birth order, family structure and childhood mental disorders. Social Psychiatry and Psychiatric Epidemiology. 2013;48(8):1327-1333.

35. Joelsson P, Chudal R, Uotila J, Suominen A, Sucksdorff D, Gyllenberg D, et al. Parental psychopathology and offspring attention-deficit/hyperactivity disorder in a nationwide sample. J Psychiatr Res. 2017;94:124-130.

36. Park S, Cho S-C, Kim J-W, Shin M-S, Yoo H-J, Min Oh S, et al. Differential perinatal risk factors in children with attention-deficit/hyperactivity disorder by subtype. Psychiatry Res. 2014;219(3):609-616.

37. Mhalla A, Guedria A, Brahem T, Amamou B, Sboui W, Gaddour N, et al. ADHD in Tunisian Adolescents: Prevalence and Associated Factors. Journal of Attention Disorders. 2018;22(2):154-162.

38. Fuller-Thomson E, Mehta R, Valeo A. Establishing a link between attention deficit disorder/attention deficit hyperactivity disorder and childhood physical abuse. Journal of Aggression, Maltreatment and Trauma. 2014;23(2):188-198.

39. Fuller-Thomson E, Lewis DA. The relationship between early adversities and attention-deficit/hyperactivity disorder. Child Abuse Negl. 2015;47:94-101.

40. Konstenius M, Leifman A, van Emmerik-van Oortmerssen K, van de Glind G, Franck J, Moggi F, et al. Childhood trauma exposure in substance use disorder patients with and without ADHD. Addictive Behaviors. 2017; 65: 118-124..

41. Rowland AS, Skipper BJ, Rabiner DL, Qeadan F, Campbell RA, Naftel AJ, et al. Attention-Deficit/Hyperactivity Disorder (ADHD): Interaction between socioeconomic status and parental history of ADHD determines prevalence. Journal of child psychology and psychiatry, and allied disciplines. 2018;59(3):213-222.

42. Lara C, Fayyad J, de Graaf R, Kessler RC, Aguilar-Gaxiola S, Angermeyer M, et al. Childhood predictors of adult attention-deficit/hyperactivity disorder: results from the World Health Organization World Mental Health Survey Initiative. Biol Psychiatry. 2009;65(1):46-54.

43. Sugaya L, Hasin DS, Olfson M, Lin K-H, Grant BF, Blanco C. Child physical abuse and adult mental health: A national study. Journal of Traumatic Stress. 2012; 25(4): 384-392..

44. Kim HW, Cho SC, Kim BN, Kim JW, Shin MS, Kim Y. Perinatal and familial risk factors are associated with full syndrome and subthreshold attention-deficit hyperactivity disorder in a korean community sample. Psychiatry Investig. 2009;6(4):278-285.

45. Islam MI, Khanam R, Kabir E. Bullying victimization, mental disorders, suicidality and self-harm among Australian high schoolchildren: Evidence from nationwide data. Psychiatry Res. 2020;292:113364.

46. Uddin J, Alharbi N, Uddin H, Hossain MB, Hatipoglu SS, Long DL, et al. Parenting stress and family resilience affect the association of adverse childhood experiences with children's mental health and attention-deficit/hyperactivity disorder. Journal of Affective Disorders. 2020; 272: 104-109.

47. Brown NM, Briggs RD, Germán M, Belamarich PF, Oyeku SO, Brown SN. Associations Between Adverse Childhood Experiences and ADHD Diagnosis and Severity. Academic Pediatrics. 2017;17(4):349-355.

48. Perales F, Johnson SE, Baxter J, Lawrence D, Zubrick SR. Family structure and childhood mental disorders: new findings from Australia. Social Psychiatry and Psychiatric Epidemiology. 2017;52(4):423-433.

49. Flisher AJ, Kramer RA, Hoven CW, Greenwald S, Alegria M, Bird HR, et al. Psychosocial characteristics of physically abused children and adolescents. Journal of the American Academy of Child & Adolescent Psychiatry. 1997;36(1):123-131.

50. Lola HM, Belete H, Gebeyehu A, Zerihun A, Yimer S, Leta K. Attention Deficit Hyperactivity Disorder (ADHD) among Children Aged 6 to 17 Years Old Living in Girja District, Rural Ethiopia. Behav Neurol. 2019;2019:1753580.

51. Sawyer MG, Reece CE, Sawyer ACP, Johnson SE, Lawrence D. Has the prevalence of child and adolescent mental disorders in Australia changed between 1998 and 2013 to 2014? Journal of the American Academy of Child & Adolescent Psychiatry. 2018; 57(5):343-350.

52. Bomysoad RN, Francis LA. Adverse Childhood Experiences and Mental Health Conditions Among Adolescents. Journal of Adolescent Health. 2020;67(6):868-870.

53. Farahat T, Alkot M, Rajab A, Anbar R. Attention-Deficit Hyperactive Disorder among Primary School Children in Menoufia Governorate, Egypt. Int J Family Med. 2014;2014:257369-.

54. Walker CS, Walker BH, Brown DC, Buttross S, Sarver DE. Defining the role of exposure to ACEs in ADHD: Examination in a national sample of US children. Child Abuse Negl. 2021;112:104884.

55. Crouch E, Radcliff E, Bennett KJ, Brown MJ, Hung P. Examining the Relationship Between Adverse Childhood Experiences and ADHD Diagnosis and Severity. Academic Pediatrics. 2021;21(8):1388-1394.

56. Bielas H, Barra S, Skrivanek C, Aebi M, Steinhausen H-C, Bessler C, et al. The associations of cumulative adverse childhood experiences and irritability with mental disorders in detained male adolescent offenders. Child and adolescent psychiatry and mental health. 2016;10:34-44.

57. Calthorpe LM, Pantell MS. Differences in the prevalence of childhood adversity by geography in the 2017-18 National Survey of Children’s Health. Child Abuse Negl. 2021;111:104804.

58. Montes G. Having Older Siblings Is Associated with Lower Rates of Depression, ADD/ADHD, Anxiety and Behavior Problems Among Children with ASD. Maternal and Child Health Journal. 2018;22(5):642-647.

59. Machado A, Rafaela D, Silva T, Veigas T, Cerejeira J. ADHD Among Offenders: Prevalence and Relationship With Psychopathic Traits. Journal of Attention Disorders. 2020;24(14):2021-2029..

60. Fulton BD, Scheffler RM, Hinshaw SP. State Variation in Increased ADHD Prevalence: Links to NCLB School Accountability and State Medication Laws. Psychiatric Services. 2015;66(10):1074-1082.

61. Bauermeister JJ, Shrout PE, Ramírez R, Bravo M, Alegría M, Martínez-Taboas A, et al. ADHD correlates, comorbidity, and impairment in community and treated samples of children and adolescents. Journal of abnormal child psychology. 2007;35(6):883-898.

62. Ferrer M, Andión Ó, Calvo N, Ramos-Quiroga JA, Prat M, Corrales M, et al. Differences in the association between childhood trauma history and borderline personality disorder or attention deficit/hyperactivity disorder diagnoses in adulthood. European Archives of Psychiatry and Clinical Neuroscience. 2017;267(6):541-549.

63. Siddique S, Banerjee M, Ray MR, Lahiri T. Attention-deficit hyperactivity disorder in children chronically exposed to high level of vehicular pollution. European Journal of Pediatrics. 2011;170(7):923-929.

64. Lehmann S, Havik OE, Havik T, Heiervang ER. Mental disorders in foster children: A study of prevalence, comorbidity and risk factors. Child and Adolescent Psychiatry and Mental Health. 2013; 7(1):39-51.

| **Appendix 6: Result of Meta-Analysis for the Association Between ACEs and ADHD** | | | | | | | | |
| --- | --- | --- | --- | --- | --- | --- | --- | --- |
| Meta-Analysis | No. of Study | Pooled Effect Size | 95%CI | *I*2 | *p* for heterogeneity | Begg's Test z | Pr > \|z\| | Pooled Effect Size after leave-one-out |
| all forms of ACEs & ADHD | 70 | 1.68 | 1.54-1.83 | 94.10% | 0.000 | 2.91 | 0.004 | 1.52-1.86 |
| physical abuse & ADHD | 10 | 2.04 | 1.44-2.90 | 91.50% | 0.000 | 0.72 | 0.474 | 1.28-3.21 |
| sex abuse & ADHD | 7 | 2.51 | 2.18-2.89 | 0.00% | 0.674 | 1.50 | 0.133 | 2.05-2.98 |
| emotional abuse & ADHD | 5 | 1.5 | 1.06-2.11 | 78.60% | 0.001 | 1.22 | 0.221 | 0.94-3.28 |
| neglect & ADHD | 7 | 1.52 | 1.07-2.17 | 91.50% | 0.000 | 0.00 | 1.000 | 0.96-2.52 |
| economic adversity & ADHD | 23 | 1.71 | 1.32-2.21 | 99.30% | 0.000 | 1.00 | 0.316 | 1.24-2.33 |
| single parent/parental separation/divoce & ADHD | 19 | 1.46 | 1.35-1.58 | 68.10% | 0.000 | 0.70 | 0.484 | 1.31-1.61 |
| familial death & ADHD | 7 | 1.21 | 1.13-1.28 | 0.00% | 0.904 | 0.60 | 0.548 | 1.09-1.37 |
| Stepfamily /adoption /foster placement & ADHD | 11 | 2.81 | 1.78-4.41 | 95.60% | 0.000 | 0.78 | 0.436 | 1.58-4.85 |
| familial criminality /incarceration & ADHD | 11 | 1.7 | 1.45-1.99 | 95.10% | 0.000 | 0.00 | 1.000 | 1.34-2.09 |
| household substance abuse & ADHD | 11 | 1.53 | 1.31-1.79 | 79.40% | 0.000 | 0.16 | 0.876 | 1.23-1.88 |
| household mental illness & ADHD | 18 | 1.88 | 1.61-2.20 | 93.20% | 0.000 | 0.45 | 0.649 | 1.55-2.28 |
| family conflict or discord & ADHD | 7 | 2.31 | 1.79-2.97 | 96.70% | 0.000 | 0.00 | 1.000 | 1.55-4.15 |
| domestic violence & ADHD | 10 | 1.47 | 1.12-1.95 | 89.50% | 0.000 | 0.54 | 0.592 | 1.05-2.08 |
| bullying & ADHD | 6 | 1.73 | 1.21-2.48 | 80.90% | 0.000 | 1.88 | 0.060 | 1.10-2.96 |
| discrimination & ADHD | 3 | 1.35 | 1.09-1.67 | 34.80% | 0.216 | 0.00 | 1.000 | 0.99-1.99 |
| other violence & ADHD | 3 | 1.52 | 1.12-2.07 | 81.10% | 0.005 | 0.00 | 1.000 | 0.85-2.79 |
| accident/injury/illness trauma & ADHD | 3 | 1.45 | 1.02-2.06 | 19.20% | 0.290 | 1.04 | 0.296 | 0.77-2.61 |
| exposed to one ACE & ADHD | 8 | 1.51 | 1.28-1.77 | 79.20% | 0.000 | -0.25 | 0.805 | 1.22-1.83 |
| exposed to two ACEs & ADHD | 8 | 1.99 | 1.63-2.45 | 84.40% | 0.000 | 0.00 | 1.000 | 1.52-2.55 |
| exposed to three or more ACEs & ADHD | 8 | 2.87 | 2.31-3.75 | 65.70% | 0.005 | 0.00 | 1.000 | 2.15-3.80 |
| ACEs & Inattentive Symptom | 4 | 5.96 | 1.23-10.70 | 99.90% | 0.000 | 2.04 | 0.089 | 0.25-2.86 |
| ACEs & Hyperactive Symptom | 2 | 0.2 | 0.10-0.31 | 0.00% | 0.598 | 1.00 | 1.000 | 0.00-0.54 |
| ACEs & Hyperactive/impulsive Symptom | 2 | 3.39 | -12.26 | 99.90% | 0.000 | -1.00 | 1.000 | -0.00-6,75 |
| ACEs & ADHD-IN | 7 | 1.83 | 1.37-2.43 | 84.40% | 0.000 | 1.05 | 0.368 | 1.28-2.71 |
| ACEs & ADHD-HI | 5 | 1.44 | 1.26-1.64 | 0.00% | 0.417 | 0.98 | 0.462 | 1.21-1.77 |
| ACEs & ADHD-C | 4 | 2.12 | 1.28-3.51 | 67.10% | 0.028 | 0.68 | 0.734 | 1.06-4.83 |

**Appendix 7: Forest Plots and Funnel Plots of Meta-Analysis for the Association Between 17 Forms of Common ACEs and ADHD**


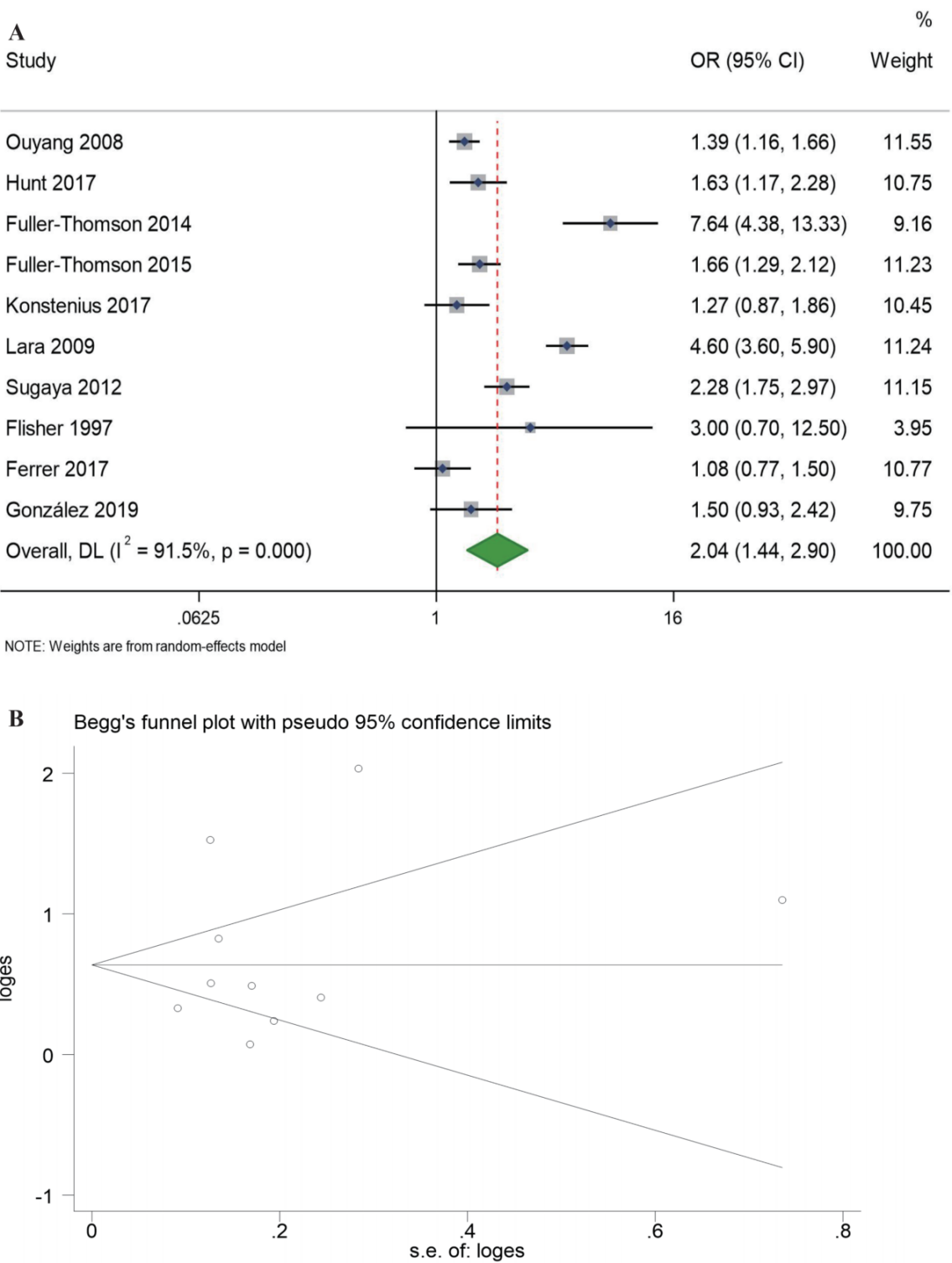


Figure S1. Result of meta-analysis for the association between physical abuse and ADHD

1. Primary analysis for the association between physical abuse and ADHD
2. Begg’s funnel plot of meta-analysis for the association between physical abuse and ADHD

*Note*: each dot represents one study.


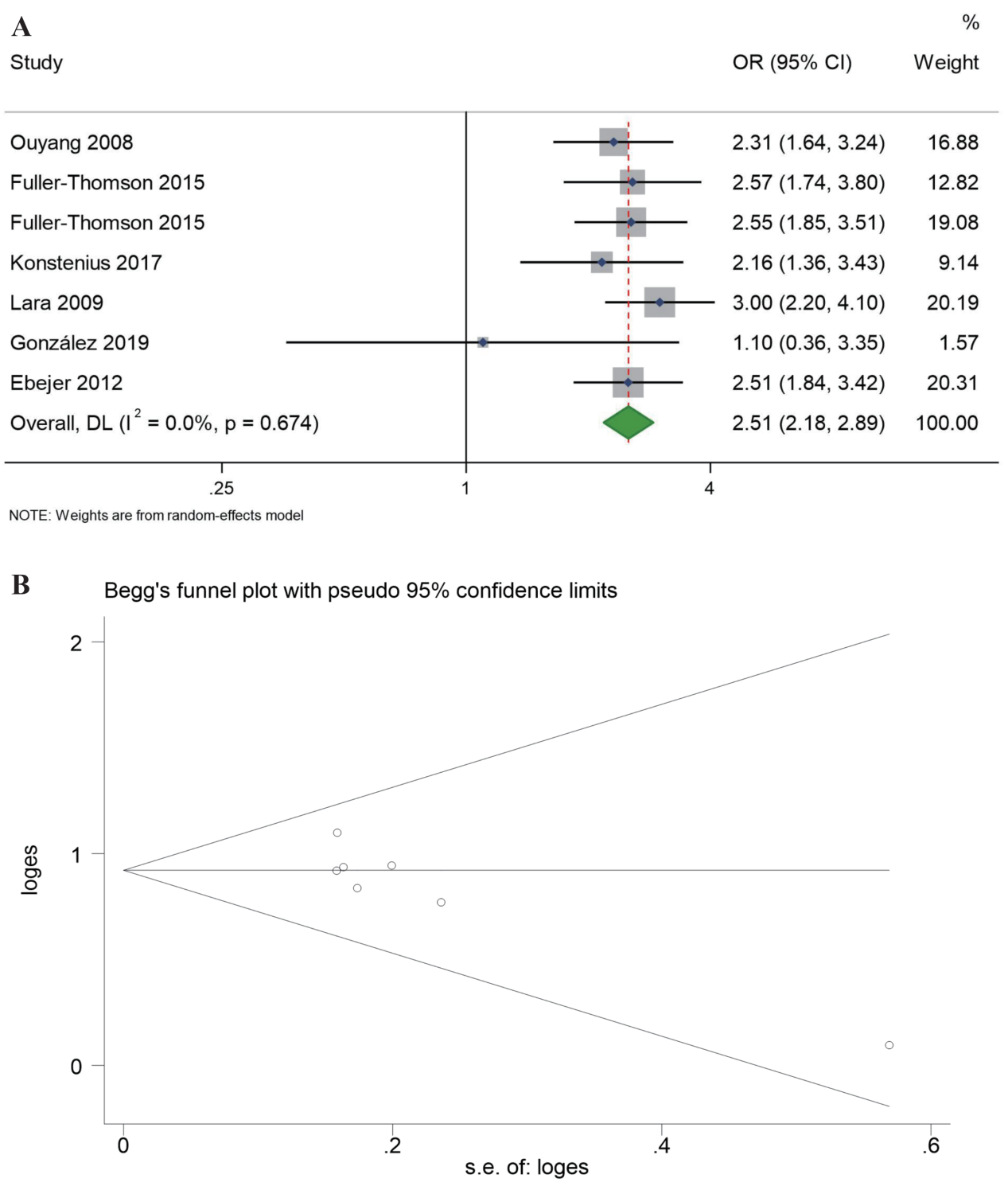


Figure S2. Result of meta-analysis for the association between sex abuse and ADHD

1. Primary analysis for the association between sex abuse and ADHD
2. Begg’s funnel plot of meta-analysis for the association between sex abuse and ADHD

*Note*: each dot represents one study.


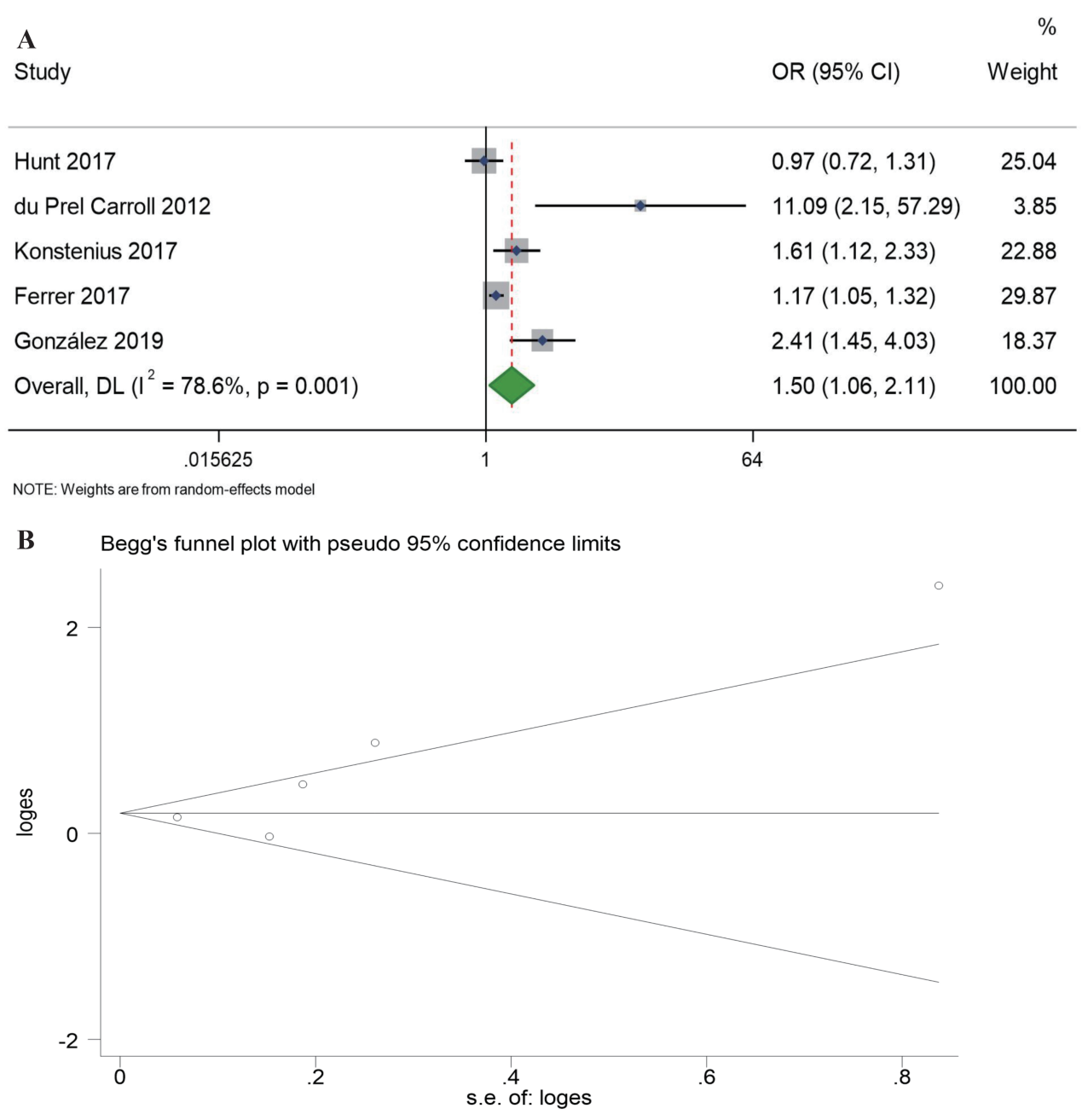


Figure S3. Result of meta-analysis for the association between emotional abuse and ADHD

1. Primary analysis for the association between emotional abuse and ADHD
2. Begg’s funnel plot of meta-analysis for the association between emotional abuse and ADHD

*Note*: each dot represents one study.


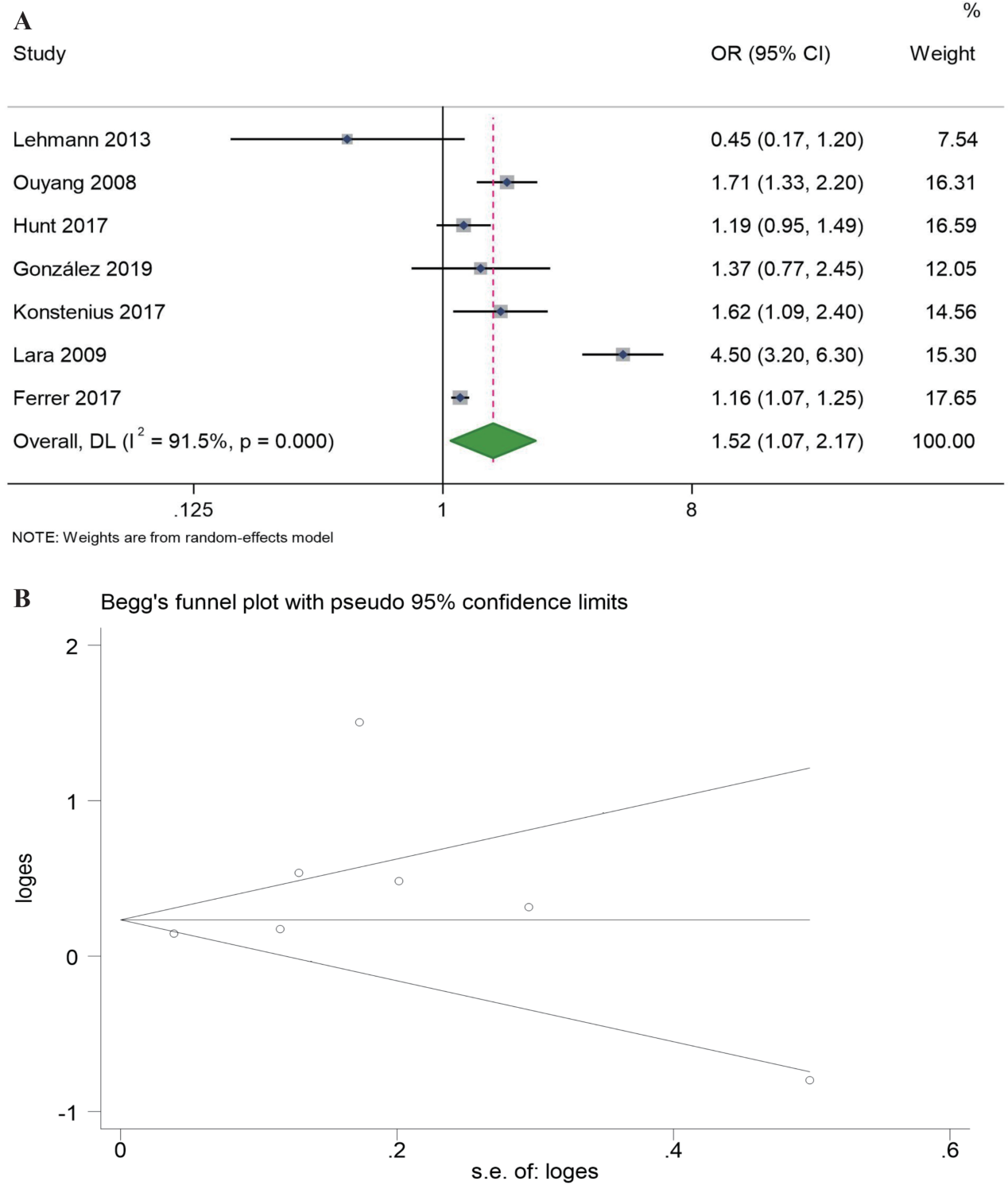


Figure S4. Result of meta-analysis for the association between neglect and ADHD

1. Primary analysis for the association between neglect and ADHD
2. Begg’s funnel plot of meta-analysis for the association between neglect and ADHD

*Note*: each dot represents one study.


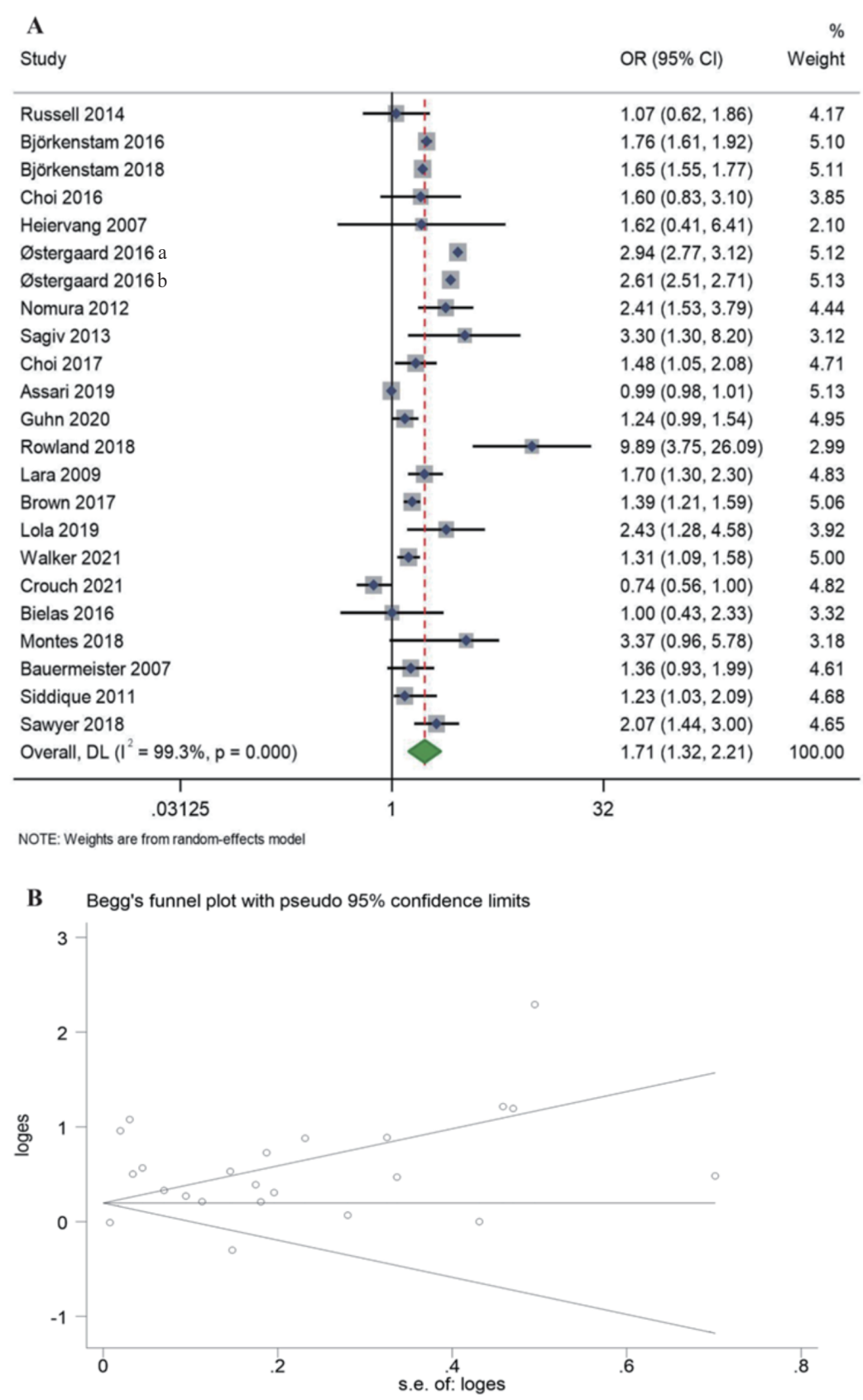


Figure S5. Result of meta-analysis for the association between economic adversity and ADHD

1. Primary analysis for the association between economic adversityt and ADHD
2. Begg’s funnel plot of meta-analysis for the association between economic adversityt and ADHD

*Note*: each dot represents one study.


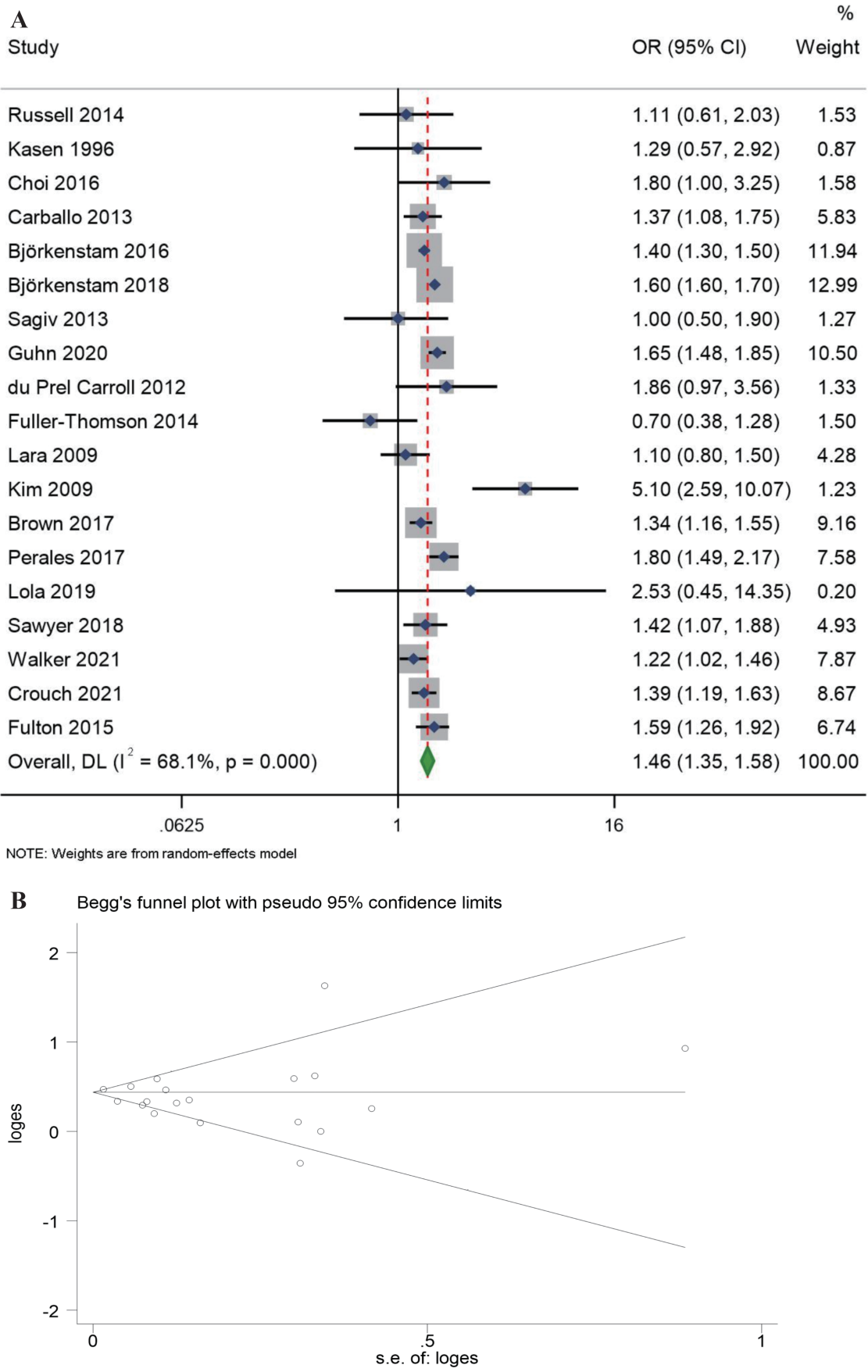


Figure S6. Result of meta-analysis for the association between single parent / parental separation / divoce and ADHD

1. Primary analysis for the association between single parent / parental separation / divoce and ADHD
2. Begg’s funnel plot of meta-analysis for the association between single parent / parental separation / divoce and ADHD

*Note*: each dot represents one study.


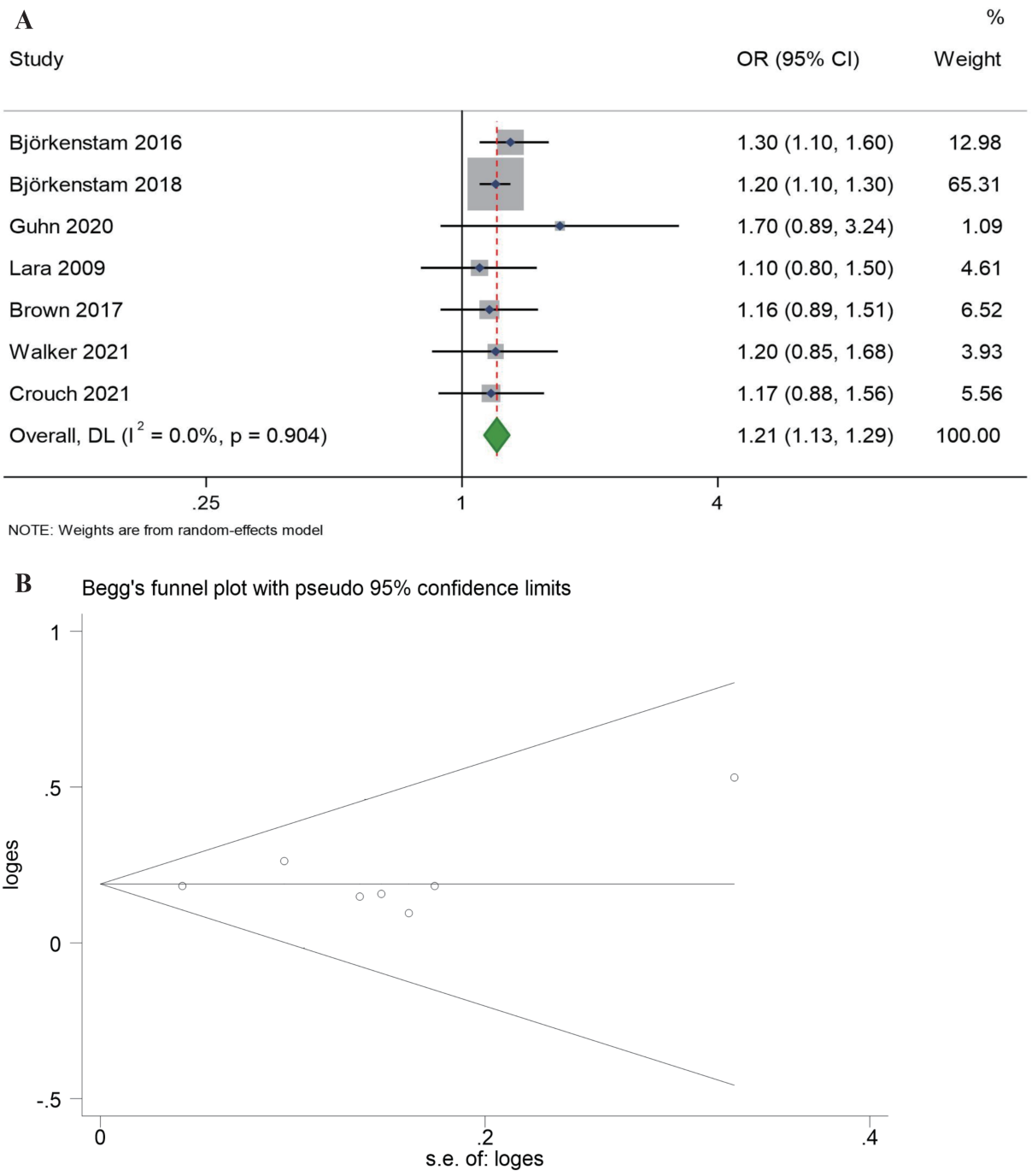


Figure S7. Result of meta-analysis for the association between familial death and ADHD

1. Primary analysis for the association between familial death and ADHD
2. Begg’s funnel plot of meta-analysis for the association between familial death and ADHD

*Note*: each dot represents one study.


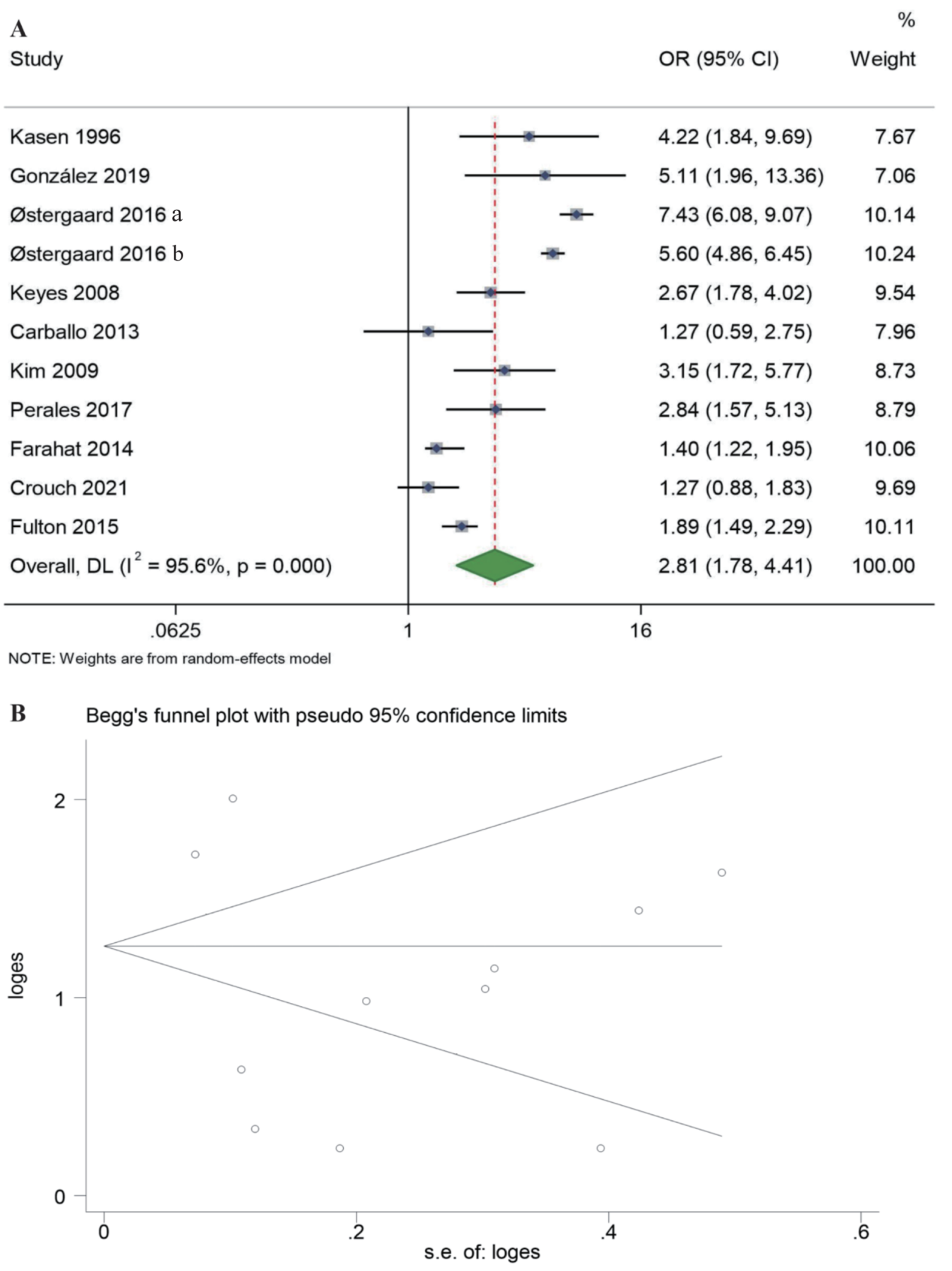


Figure S8. Result of meta-analysis for the association between stepfamily / adoption / foster placement and ADHD

1. Primary analysis for the association between stepfamily / adoption / foster placement and ADHD
2. Begg’s funnel plot of meta-analysis for the association between stepfamily / adoption / foster placement and ADHD

*Note*: each dot represents one study.


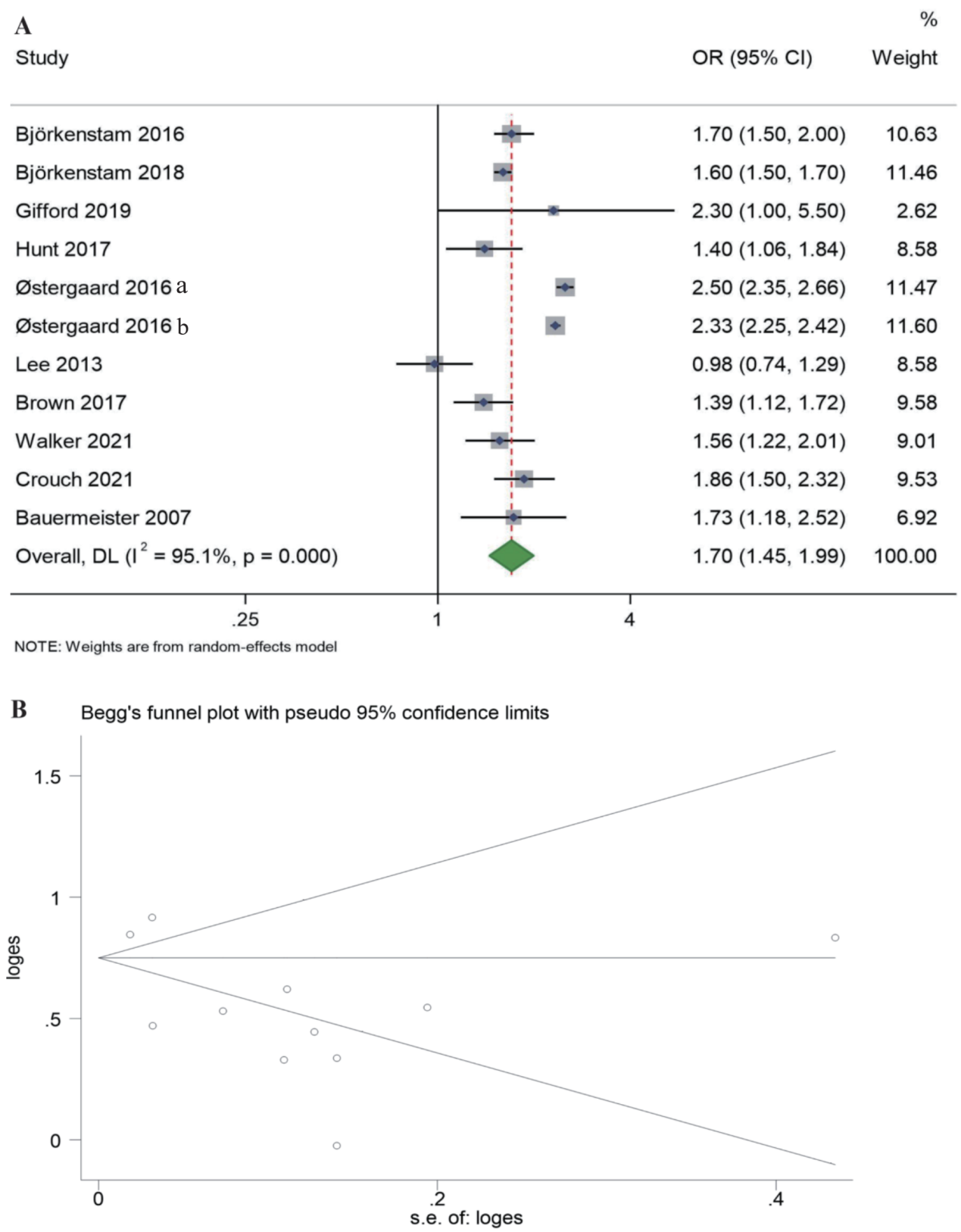


Figure S9. Result of meta-analysis for the association between familial criminality / incarceration and ADHD

1. Primary analysis for the association between familial criminality / incarceration and ADHD
2. Begg’s funnel plot of meta-analysis for the association between familial criminality / incarceration and ADHD

*Note*: each dot represents one study.


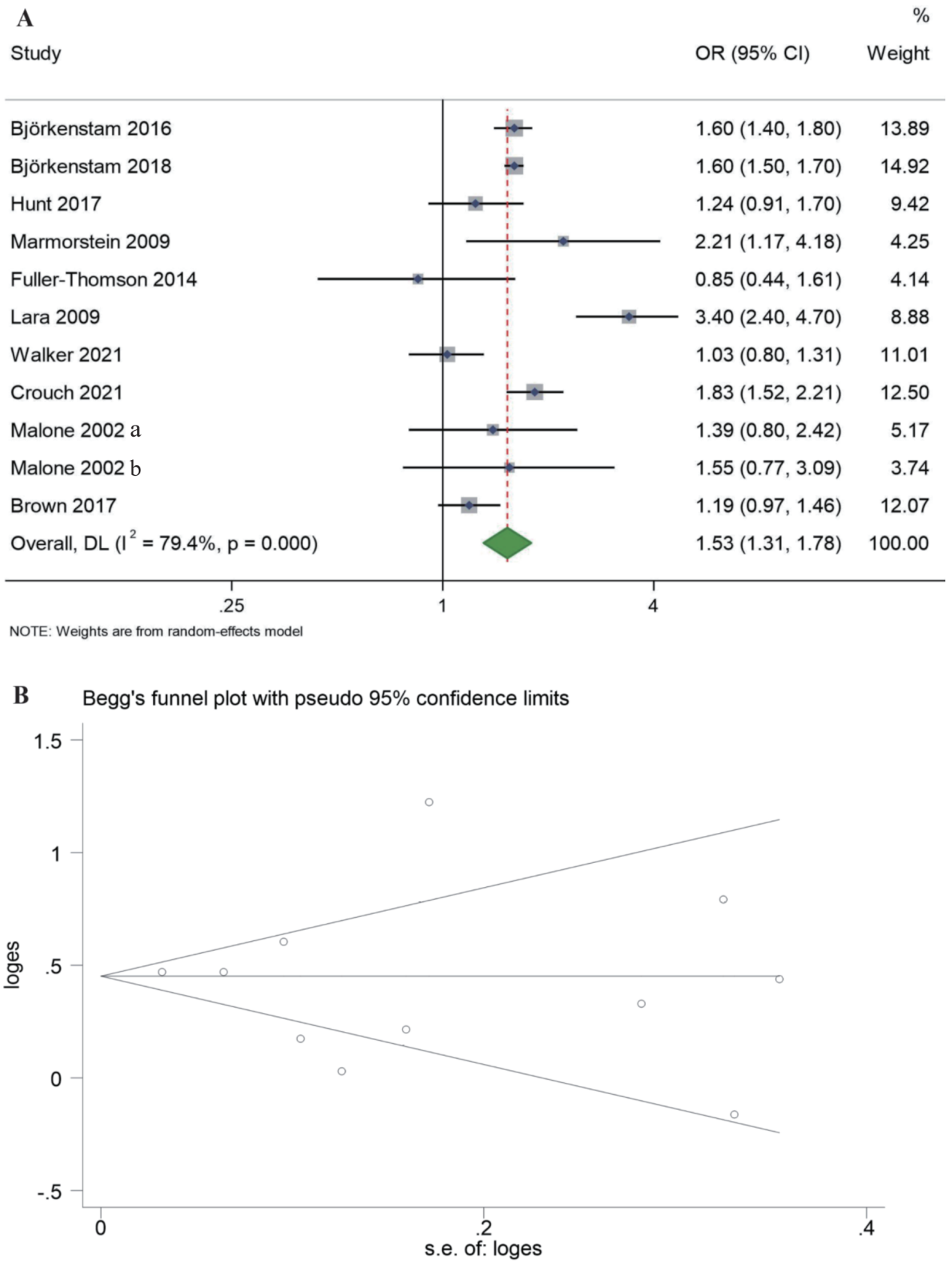


Figure S10. Result of meta-analysis for the association between household substance abuse and ADHD

1. Primary analysis for the association between household substance abuse and ADHD
2. Begg’s funnel plot of meta-analysis for the association between household substance abuse and ADHD

*Note*: each dot represents one study.


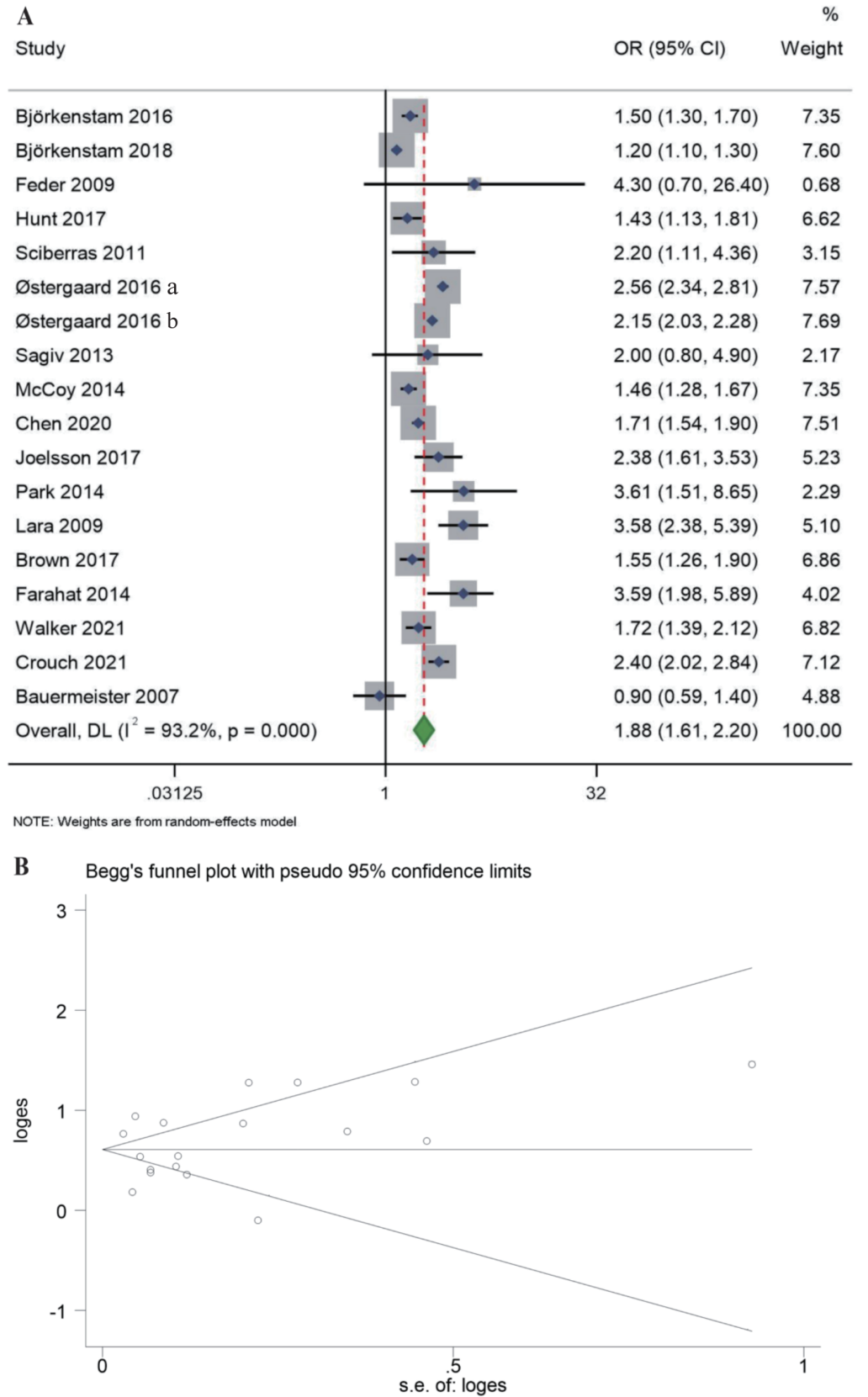


Figure S11. Result of meta-analysis for the association between household mental illness and ADHD

1. Primary analysis for the association between household mental illness and ADHD
2. Begg’s funnel plot of meta-analysis for the association between household mental illness and ADHD

*Note*: each dot represents one study.


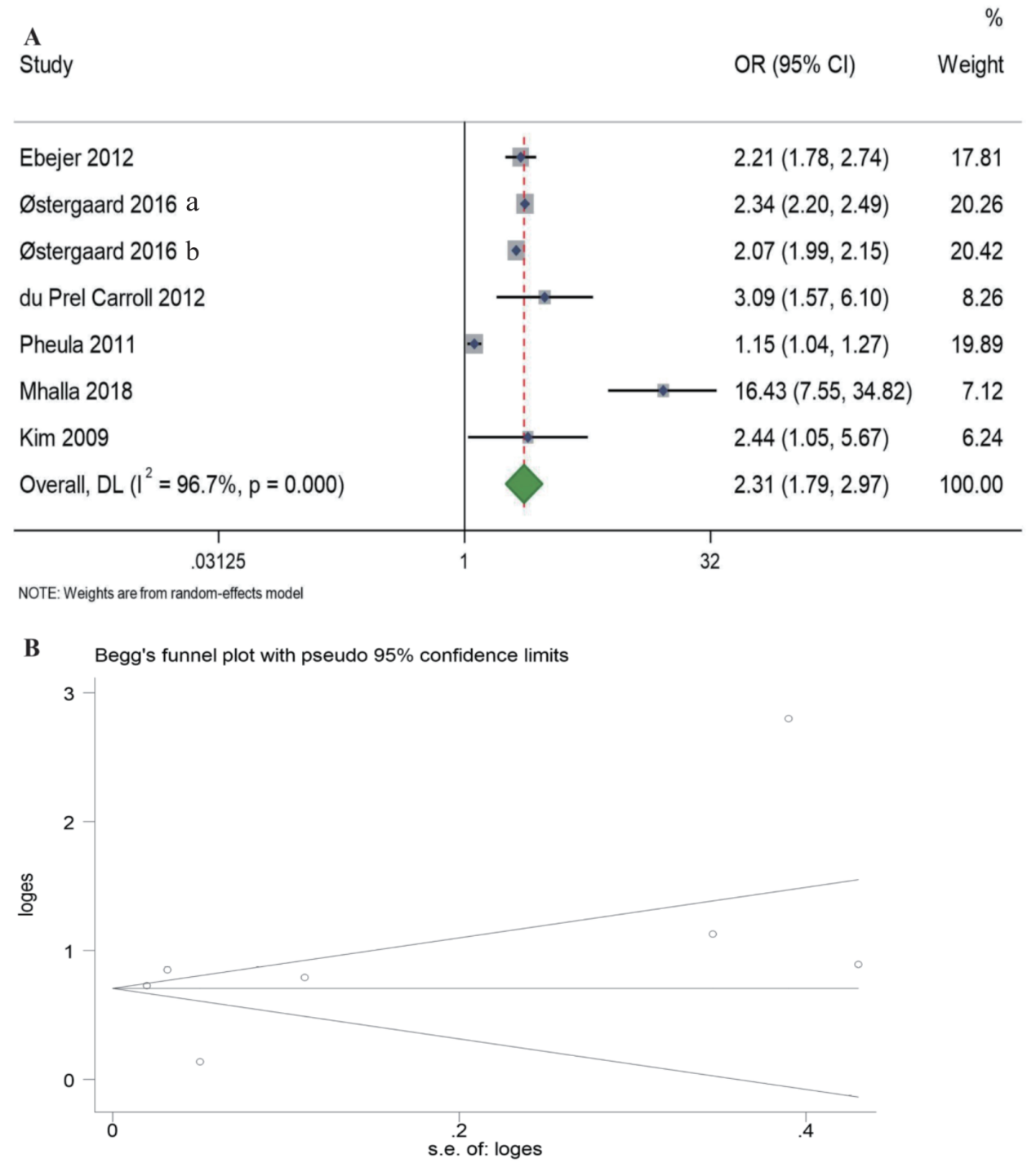


Figure S12. Result of meta-analysis for the association between family discord and ADHD

1. Primary analysis for the association between family discord and ADHD
2. Begg’s funnel plot of meta-analysis for the association between family discord and ADHD

*Note*: each dot represents one study.


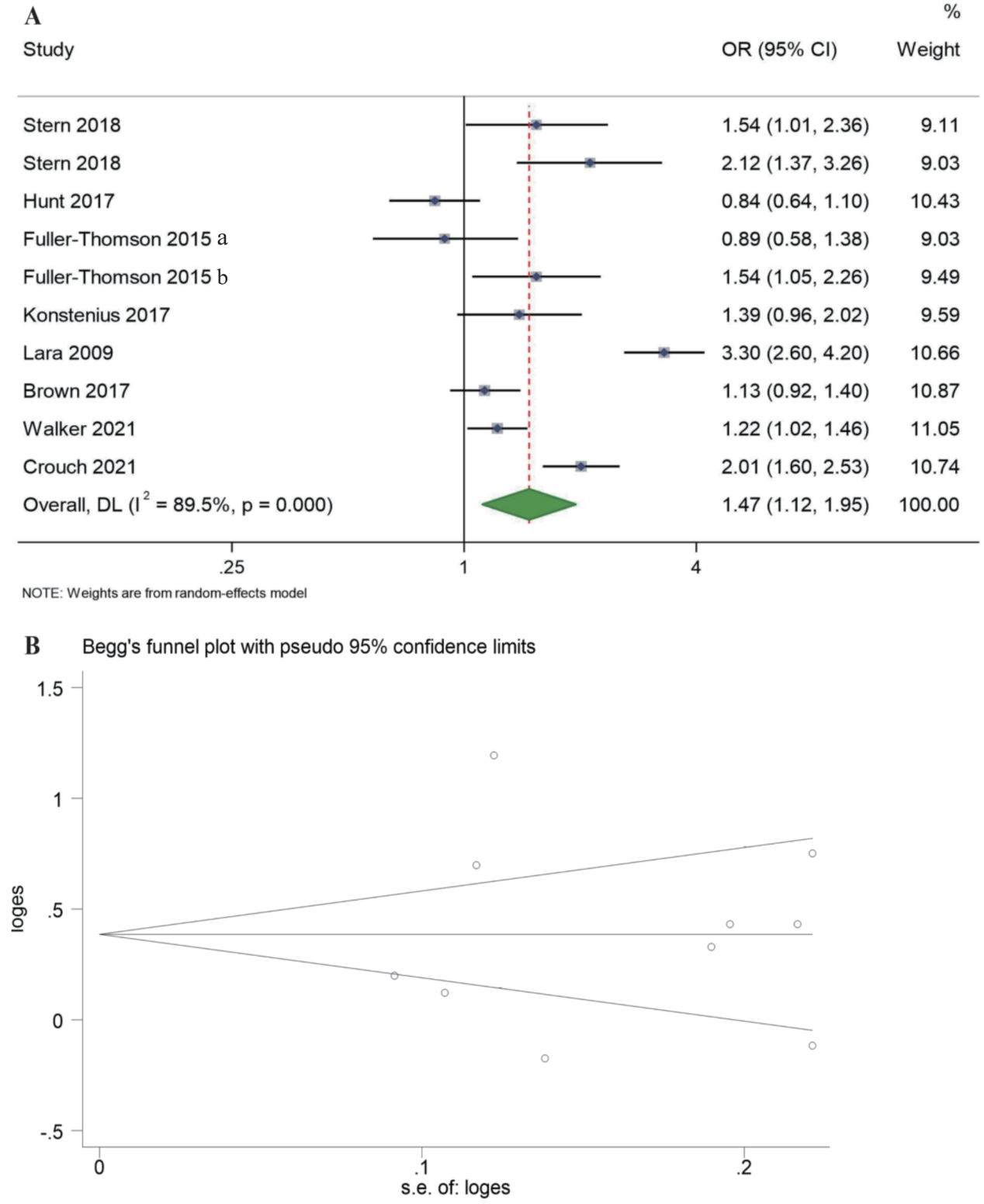


Figure S13. Result of meta-analysis for the association between domestic violence and ADHD

1. Primary analysis for the association between domestic violence and ADHD
2. Begg’s funnel plot of meta-analysis for the association between domestic violence and ADHD

*Note*: each dot represents one study.


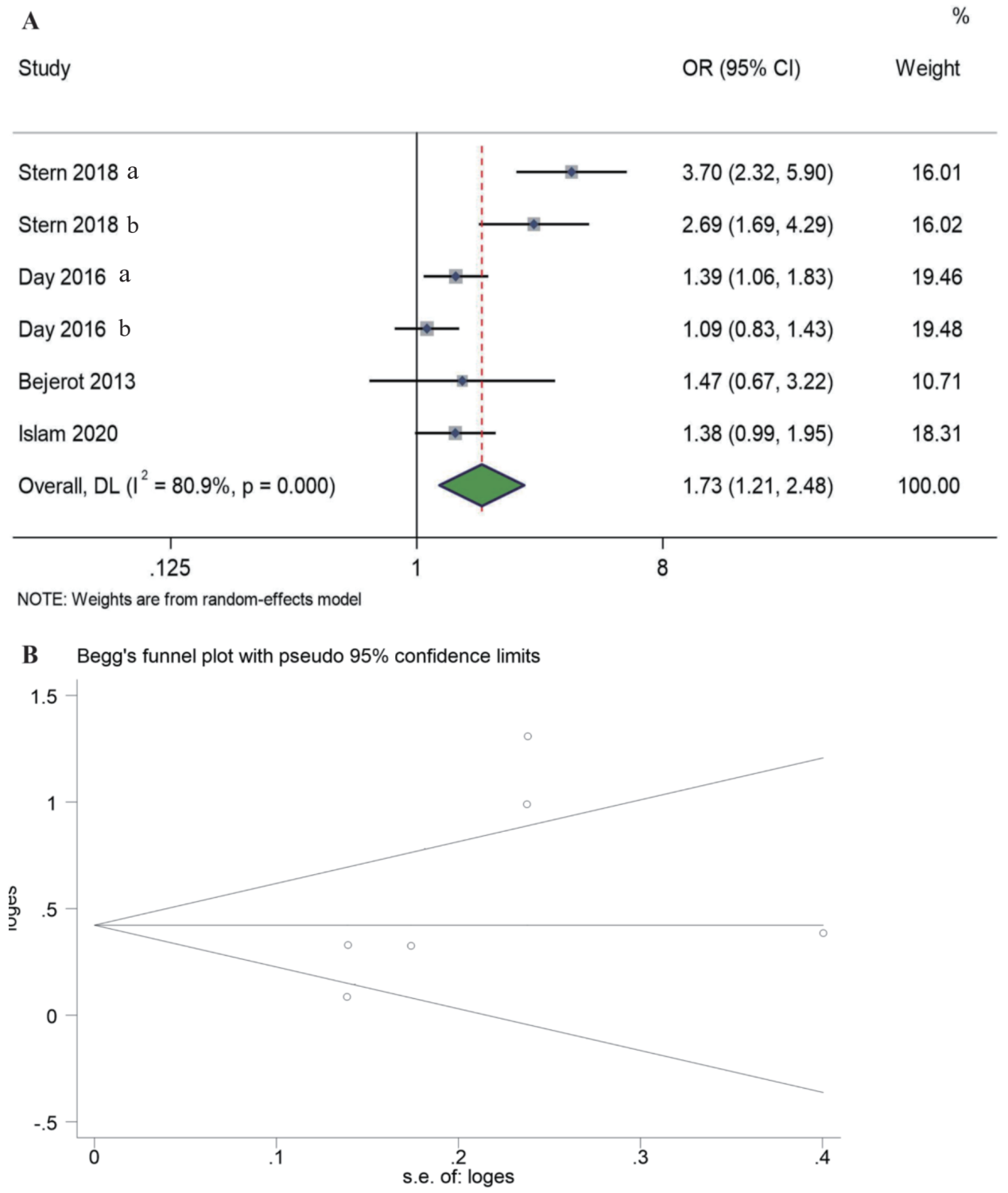


Figure S14. Result of meta-analysis for the association between bullying and ADHD

1. Primary analysis for the association between bullying and ADHD
2. Begg’s funnel plot of meta-analysis for the association between bullying and ADHD

*Note*: each dot represents one study.


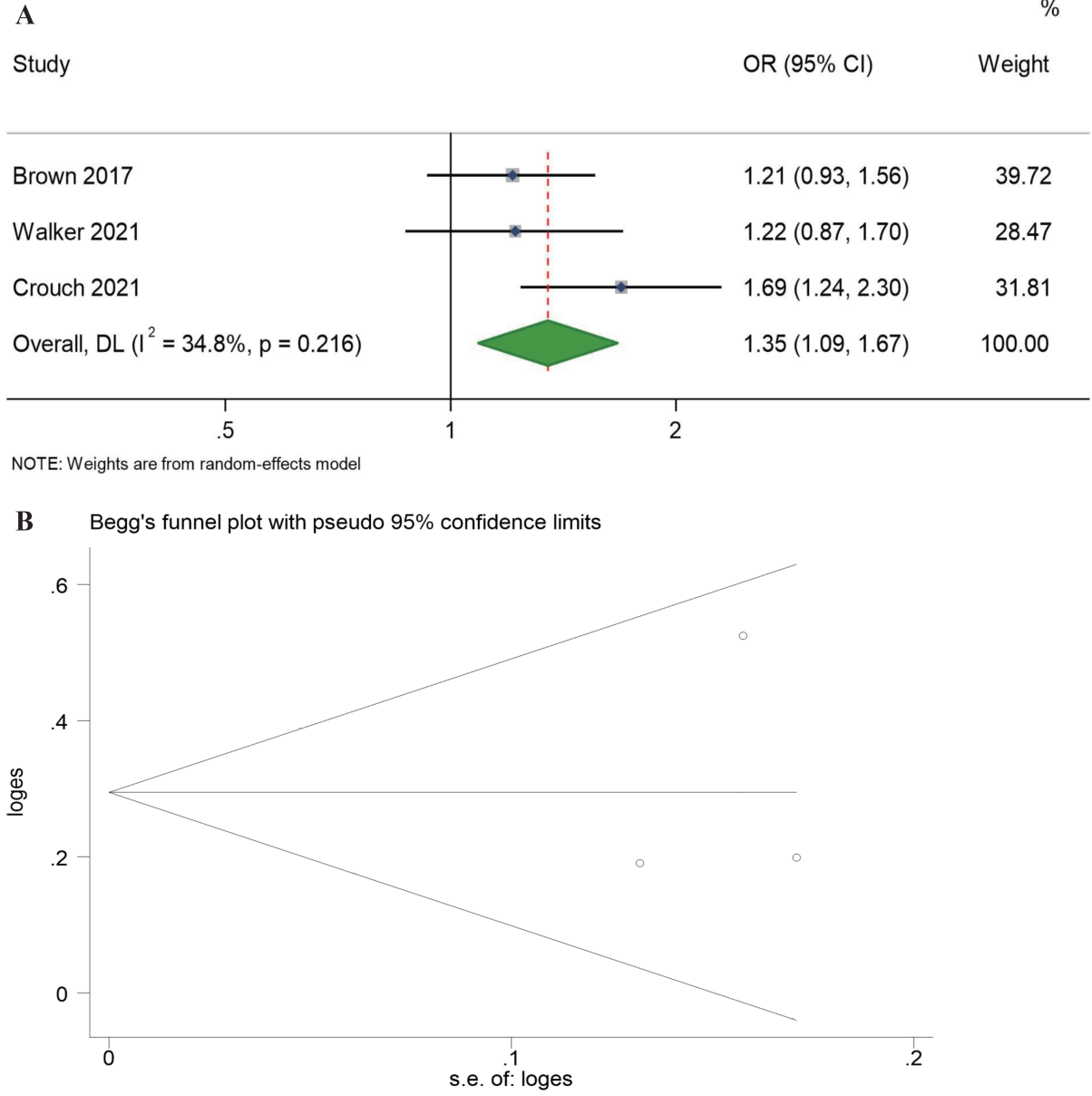


Figure S15. Result of meta-analysis for the association between discrimination and ADHD

1. Primary analysis for the association between discrimination and ADHD
2. Begg’s funnel plot of meta-analysis for the association between discrimination and ADHD

*Note*: each dot represents one study.


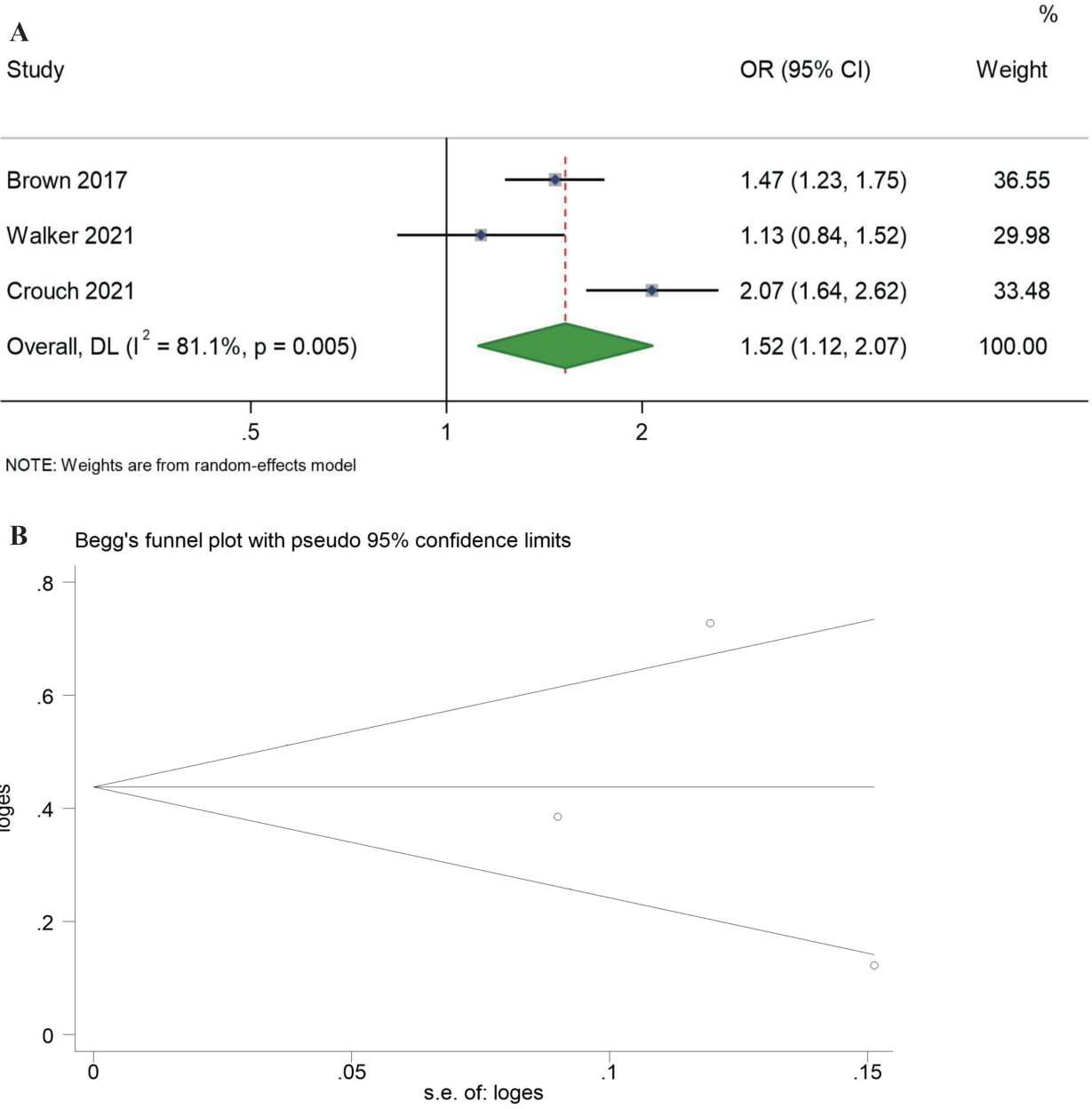


Figure S16. Result of meta-analysis for the association between community violence and ADHD

1. Primary analysis for the association between community violence and ADHD
2. Begg’s funnel plot of meta-analysis for the association between community violence and ADHD

*Note*: each dot represents one study.


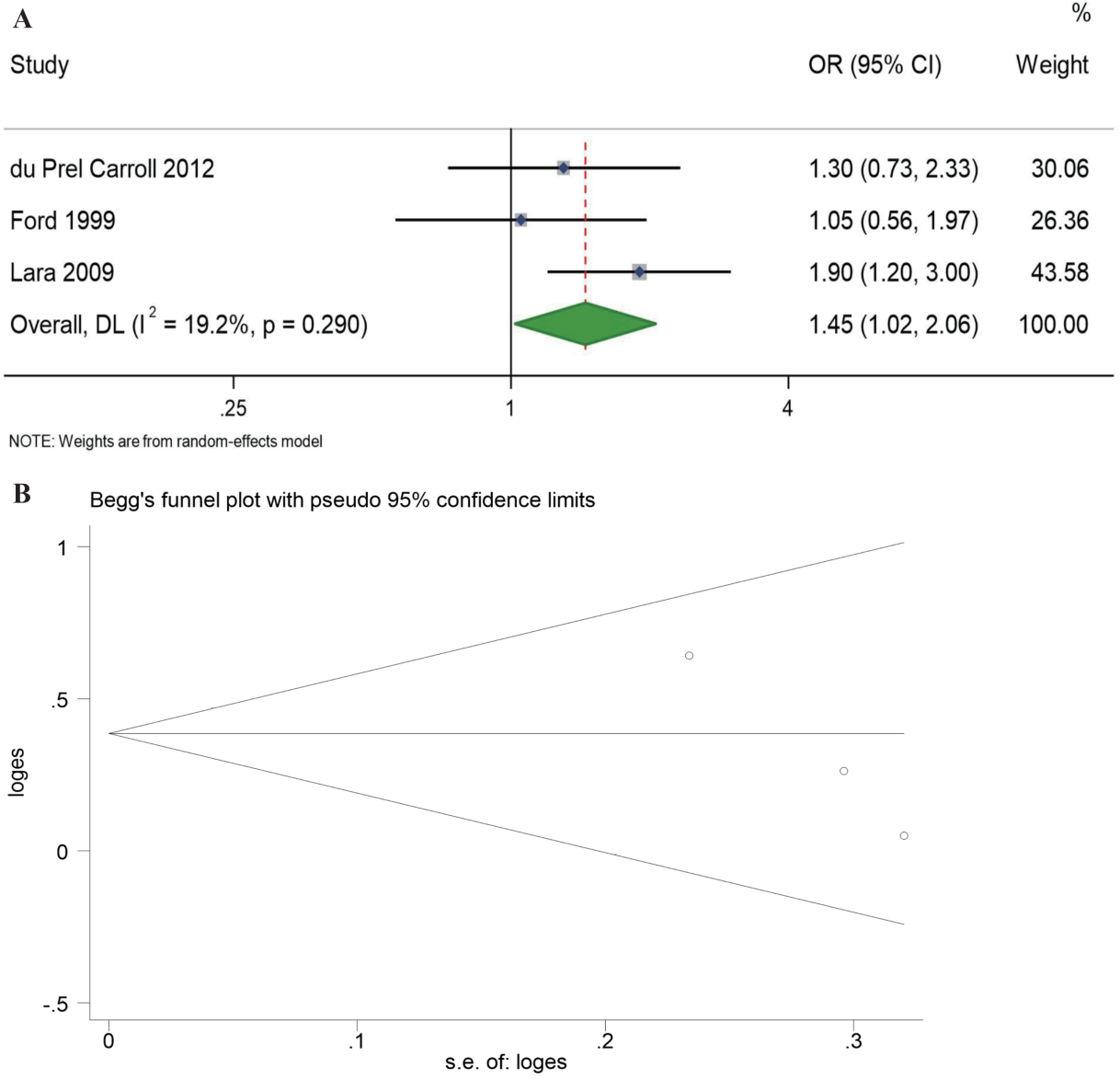


Figure S17. Result of meta-analysis for the association between accident / injury / illness trauma and ADHD

1. Primary analysis for the association between accident / injury / illness trauma violence and ADHD
2. Begg’s funnel plot of meta-analysis for the association between accident / injury / illness trauma violence and ADHD

*Note*: each dot represents one study.

| **Appendix 8: Result of Meta-Analysis for the Association Between Number of ACEs and ADHD** | | | | | | | | |
| --- | --- | --- | --- | --- | --- | --- | --- | --- |
| Meta-Analysis | No. of Study | Pooled Odds Ratio | 95%CI | *I*2 | *p* for heterogeneity | Begg's Test z | Pr > \|z\| | Pooled OR after leave-one-out |
|  |  |  |  |  |  |  |  |  |
| exposed to one ACE | 8 | 1.51 | 1.28-1.77 | 79.20% | 0.000 | -0.25 | 0.805 | 1.22-1.83 |
| exposed to two ACEs | 8 | 1.99 | 1.63-2.45 | 84.40% | 0.000 | 0.00 | 1.000 | 1.52-2.55 |
| exposed to three or more ACEs | 8 | 2.87 | 2.31-3.75 | 65.70% | 0.005 | 0.00 | 1.000 | 2.15-3.80 |

**Appendix 9: Meta-Analysis for the Association Between ACEs and ADHD symptoms**


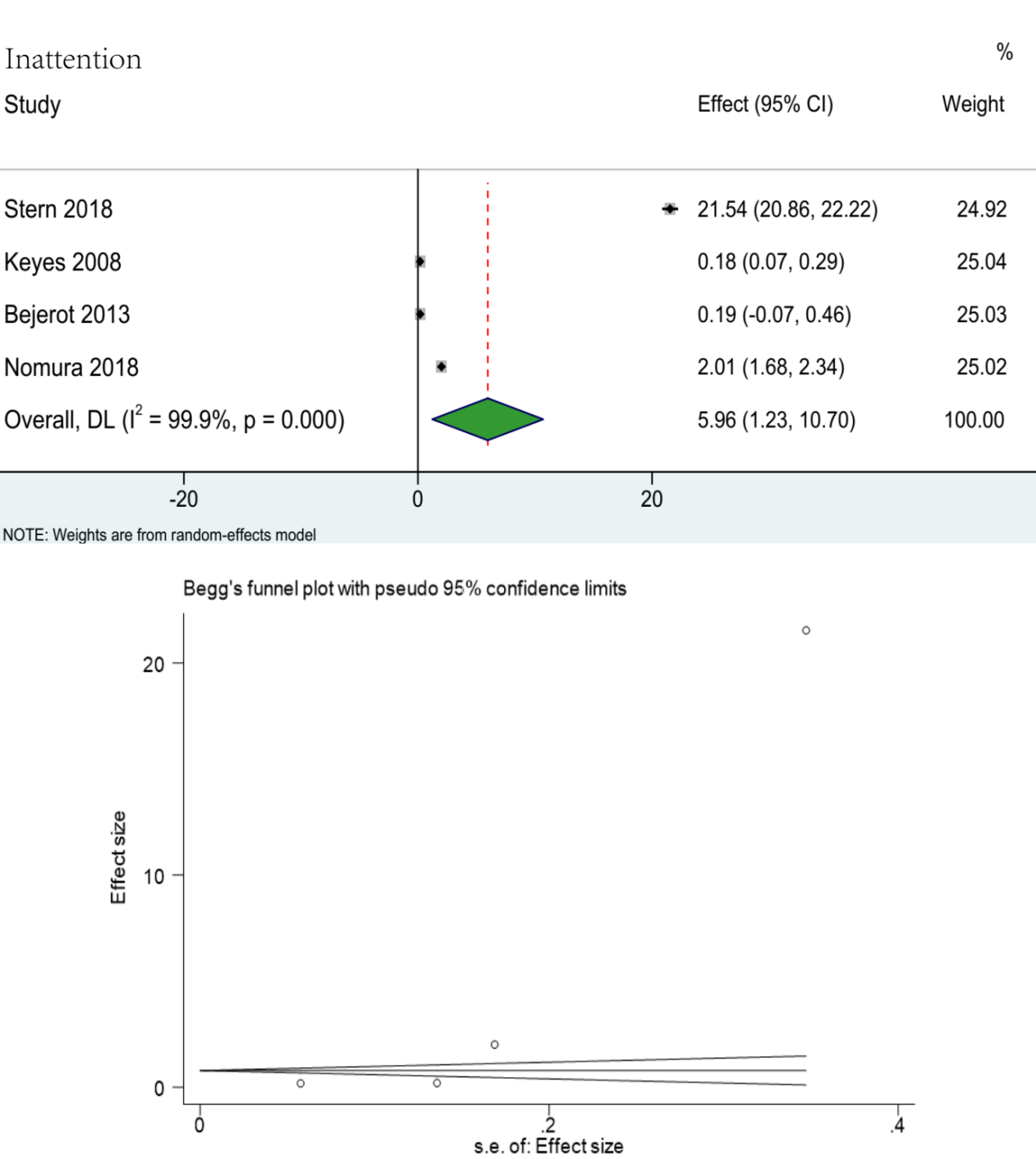


Figure S18. Result of meta-analysis for the association between ACEs and score of inattentive symptoms

*Note*: each dot represents one study.


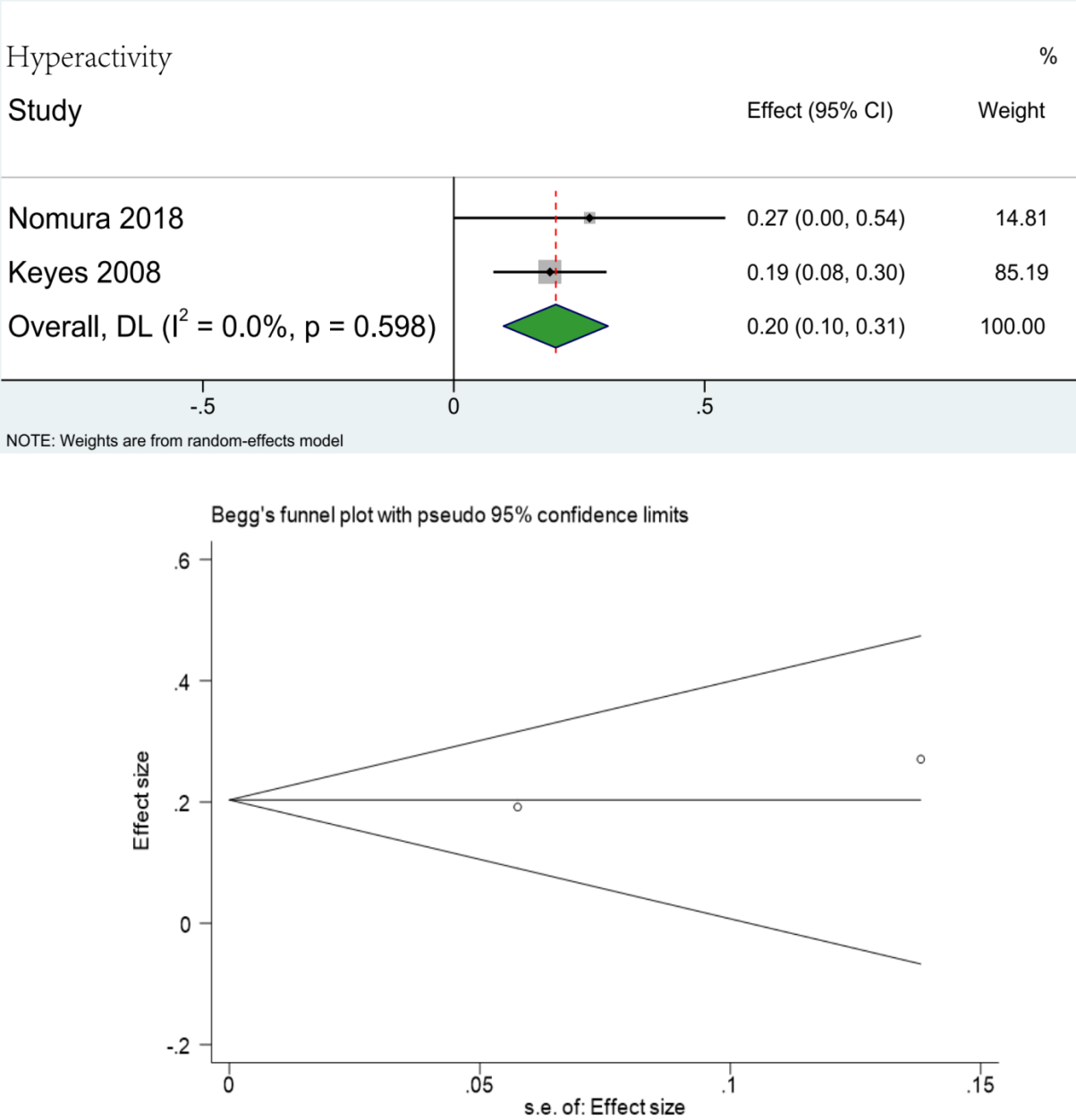


Figure S19. Result of meta-analysis for the association between ACEs and score of hyperactive symptoms

*Note*: each dot represents one study.


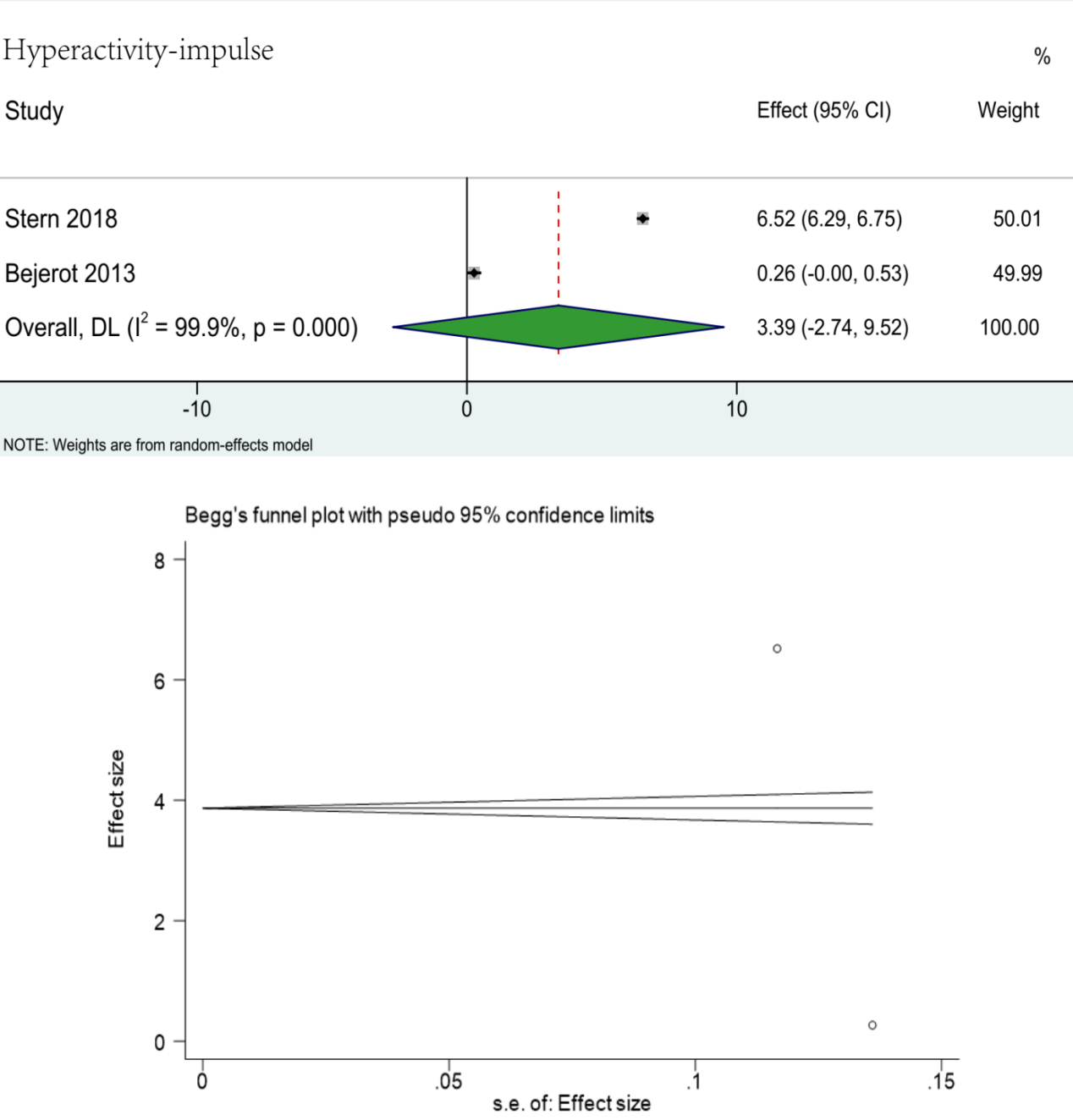


Figure S20. Result of meta-analysis for the association between ACEs and score of hyperactive-impulsive symptoms

*Note*: each dot represents one study.

**Appendix 10: Meta-Analysis for the Association Between ACEs and type of ADHD**


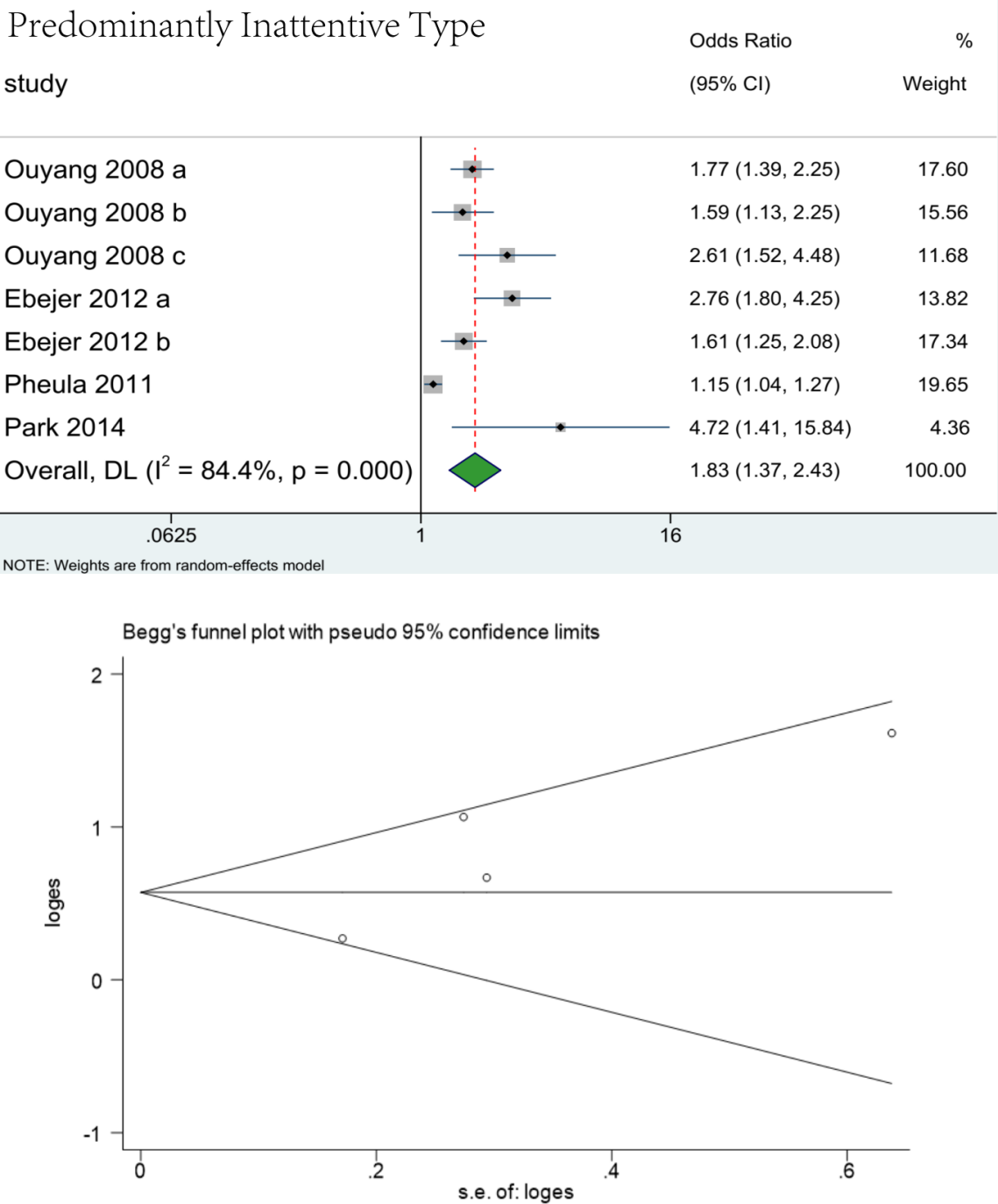


Figure S21. Result of meta-analysis for the association between ACEs and ADHD predominantly inattentive type

*Note*: each dot represents one study.


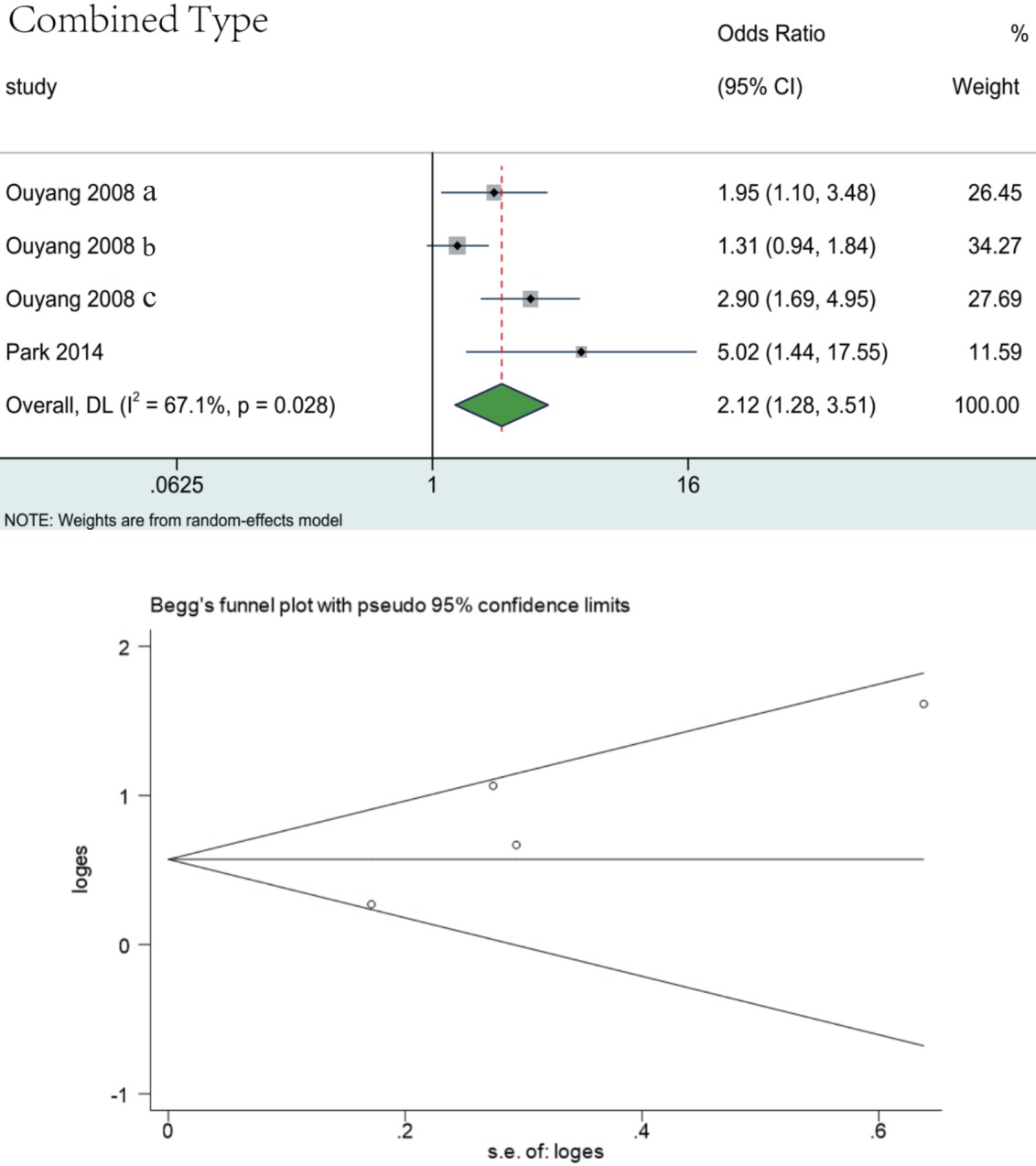


Figure S22. Result of meta-analysis for the association between ACEs and ADHD combined type

*Note*: each dot represents one study.


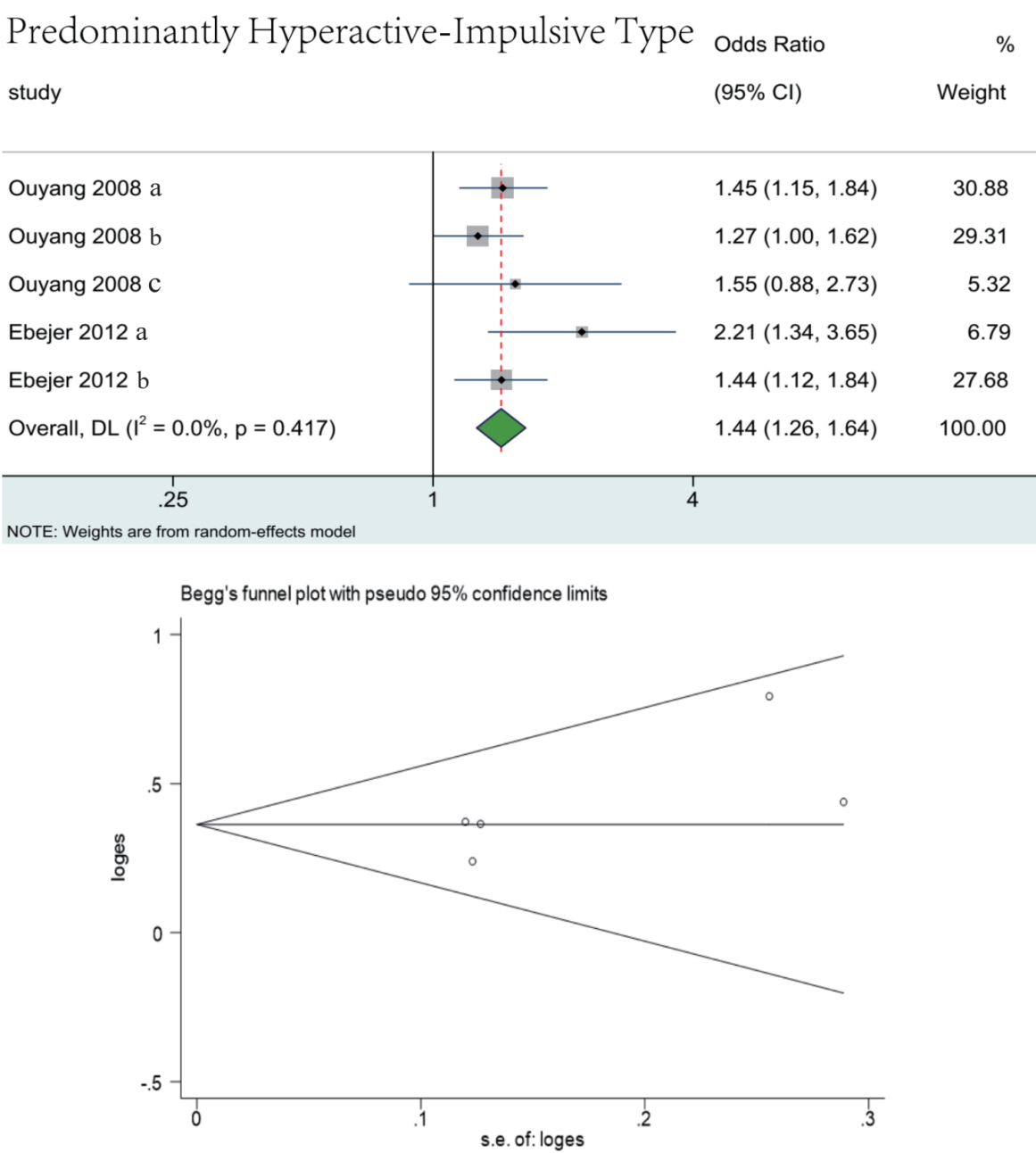


Figure S23. Result of meta-analysis for the association between ACEs and ADHD predominantly hyperactive/impulsive type

*Note*: each dot represents one study.

| **Appendix 11: Meta-Regression for Moderator Analysis** | | |
| --- | --- | --- |
| ACEs Form | Moderator Analysis | |
|  | Moderators | Statistics |
| all forms of ACEs | gender composition, | exp(b)= 1.83, SE=0.22, t(55)=5.00, p=0.000; |
|  | ADHD classification by period, | exp(b)= 1.57, SE=0.15, t(70)=4.85, p=0.000; |
|  | sample size, | exp(b)= 1.67, SE=0.08, t(69)=11.12, p=0.000; |
|  | study type, | exp(b)= 1.62, SE=0.22, t(70)=3.51, p=0.001; |
|  | study design, | exp(b)= 1.72, SE=0.26, t(70)=3.60, p=0.001; |
|  | effect estimation, | exp(b)= 1.52, SE=0.14, t(70)=4.57, p=0.000; |
|  | measure of ADHD, | exp(b)= 1.50, SE=0.13, t(70)=4.62, p=0.000; |
|  | measure of ACEs | exp(b)= 1.52, SE=0.15, t(69)=4.22, p=0.000 |
| physical abuse | ADHD classification by period, | exp(b)= 2.03, SE=0.59, t(10)= 2.44,p=0.041; |
|  | measure of ADHD | exp(b)= 2.62, SE=0.95, t(10)= 2.64,p=0.030 |
| sex abuse | NA | NA |
| emotional abuse | NA | NA |
| neglect | NA | NA |
| economic adversity | ADHD classification by period, | exp(b)= 1.55, SE=0.17, t(23)= 3.88,p=0.001; |
|  | sample size, | exp(b)= 1.52, SE=0.17, t(23)=3.80,p=0.001; |
|  | study design, | exp(b)= 1.59, SE=0.24, t(23)=3.12,p=0.005 ; |
|  | effect estimation | exp(b)= 2.16, SE=0.43, t(23)=3.85,p=0.001 |
| single parent / parental separation / divoce | location of study, | exp(b)= 1.48, SE=0.17, t(19)=3.37,p= 0.004; |
|  | sample size, | exp(b)= 1.43, SE=0.09, t(19)=5.66,p= 0.000; |
|  | study type, | exp(b)= 1.50, SE=0.12, t(19)=5.06,p= 0.000; |
|  | study design, | exp(b)= 1.50, SE=0.13, t(19)=4.54,p= 0.000; |
|  | measure of ADHD, | exp(b)= 1.32, SE=0.11, t(19)=3.44,p= 0.003; |
|  | measure of ACEs | exp(b)= 1.49, SE=0.14, t(19)=4.40,p= 0.000 |
| familial death | NA | NA |
| Stepfamily / adoption / foster placement | gender composition, | exp(b)=3.51, SE=1.70, t(10)=2.59, p=0.032; |
|  | ADHD classification by period, | exp(b)=2.12, SE=0.32, t(11)=5.02, p=0.001; |
|  | sample size, | exp(b)=2.09, SE=0.35, t(11)=4.42, p=0.002; |
|  | study type, | exp(b)=1.78, SE=0.29, t(11)=3.50, p=0.007; |
|  | effect estimation | exp(b)=2.12, SE=0.32, t(11)= 5.02, p=0.001 |
| familial criminality / incarceration | gender composition, | exp(b)=1.87, SE=0.33, t(10)=3.57,p=0.007; |
|  | location of study, | exp(b)=2.00, SE=0.21, t(11)=6.73,p=0.000; |
|  | sample size, | exp(b)=1.59, SE=0.15, t(10)=4.93,p=0.001; |
|  | study type, | exp(b)=1.62, SE=0.24, t(11)=3.28,p=0.010; |
|  | study design, | exp(b)=1.62, SE=0.24, t(11)=3.28,p=0.010; |
|  | effect estimation, | exp(b)=2.17, SE=0.22, t(11)=7.63,p=0.000; |
|  | measure of ADHD | exp(b)=1.55, SE=0.19, t(11)=3.63,p=0.007 |
| household substance abuse | location of study, | exp(b)=1.60, SE=0.20, t(11)=3.68,p=0.006; |
|  | sample size, | exp(b)=1.52, SE=0.21, t(11)=3.09,p=0.013; |
|  | study type, | exp(b)=1.50, SE=0.25, t(11)=2.42,p=0.039; |
|  | study design, | exp(b)=1.50, SE=0.25, t(11)=2.42,p=0.039; |
|  | measure of ADHD | exp(b)=1.60, SE=0.27, t(11)=2.72,p=0.026 |
| household mental illness | gender composition, | exp(b)=1.90, SE=0.48, t(18)=2.56,p=0.024; |
|  | ADHD classification by period, | exp(b)=1.91, SE=0.21, t(18)=5.77,p=0.000; |
|  | location of study, | exp(b)=3.58, SE=1.30, t(18)=3.53,p=0.004; |
|  | sample size, | exp(b)=2.03, SE=0.23, t(18)=6.16,p=0.000; |
|  | study type, | exp(b)=2.14, SE=0.28, t(18)=5.87,p=0.000; |
|  | study design, | exp(b)=2.00, SE=0.30, t(18)=4.70,p=0.000; |
|  | measure of ADHD, | exp(b)=1.79, SE=0.25, t(18)=4.07,p= 0.001; |
|  | measure of ACEs | exp(b)=2.16, SE=0.61, t(18)=2.75,p= 0.015 |
| family conflict or discord | NA | NA |
| domestic violence | measure of ADHD | exp(b)=2.02 , SE=0.38, t(10)=3.74,p=0.006 |
| bullying | NA | NA |
| discrimination | NA | NA |
| other violence | NA | NA |
| accident/injury/illness trauma | NA | NA |
| exposed to one ACE | NA | NA |
| exposed to two ACEs | NA | NA |
| exposed to three or more ACEs | NA | NA |

Abbreviations: NA, No application(n<10).

*Notes:* The following demographic factors were coded when available (high heterogeneous was detected and the number of included studies not less than 10): (1) gender composition (proportion of male participants). (2) ADHD classification by period (ADHD in childhood, adulthood or both childhood and adulthood). (3) location of study (continents of eligible studies, included Asia, Europe, North America, South America, Africa, Oceania, or multiple continents). The following methodological factors were coded when available (n≥10): (1) sample size. (2) study type (cohort, case-control, or cross-section). (3) study design (prospective, or retrospective). (4) study quality (points of quality assessment based on NOS or adapted version of NOS). (5) effect estimation (Odds ratio (OR), risk ratio (RR), or hazard ratio (HR)). (6) measure of ADHD (based on diagnostic interview / questionnaire / scale / inventory, reliable records, or self-/guardian-report of diagnosis by a professional, mixed methods). (7) measure of ACEs (based on diagnostic interview / questionnaire / scale / inventory, reliable records, or self / guardian-report diagnosis by a professional, mixed methods).

| **Appendix 12: Stratification Analysis for Potential Moderators** | | | |
| --- | --- | --- | --- |
| ACEs | Moderators | Stratification Analysis | |
|  |  | Stratification | OR(95%CI) |
| All 17 forms of ACEs | gender composition (males%) | Above SQ | 1.520(1.247-1.853) |
|  |  | IQ-SQ | 1.534(1.420-1.658) |
|  |  | Below IQ | 1.897(1.514-2.376) |
|  | ADHD classification  by period | childhood | 1.648(1.503-1.808) |
|  |  | adulthood | 1.570(1.285-1.919) |
|  |  | both | 1.933(1.491-2.507) |
|  | sample size | Above SQ | 1.707(1.525-1.912) |
|  |  | IQ-SQ | 1.776(1.521-2.073) |
|  |  | Below IQ | 1.367(1.195-1.565) |
|  | study type | Cohort Study | 1.689(1.454-1.962) |
|  |  | Case-control Study | 1.721(1.279-2.316) |
|  |  | Cross-section Study | 1.619(1.467-1.786) |
|  | study design | Prospective | 1.670(1.434-1.944) |
|  |  | Retrospective | 1.638(1.497-1.793) |
|  | effect estimation | OR | 1.631(1.499-1.775) |
|  |  | RR | 1.727(1.188-2.511) |
|  |  | HR | 1.913(1.513-2.418) |
|  | measure of ADHD | Interview / questionnaire / scale / inventory-based | 1.717(1.542-1.912) |
|  |  | Record-based | 1.778(1.490-2.123) |
|  |  | Self / caregiver-report | 1.462(1.282-1.669) |
|  | measure of ACEs | Interview / questionnaire / scale / inventory-based | 1.662(1.502-1.838) |
|  |  | Record-based | 1.829(1.536-2.164) |
|  |  | Self / caregiver-report | 1.455(1.257-1.685) |
|  |  | Mixed | 1.956(0.984-3.888) |
| physical abuse | ADHD classification  by period | Childhood | 2.085(1.147-3.790) |
|  |  | Adulthood | 2.007(1.244-3.237) |
|  |  | Both | - |
|  | measure of ADHD | Interview / questionnaire / scale / inventory-based | 1.835(1.176-2.863) |
|  |  | Record-based | - |
|  |  | Self / caregiver-report | 2.626(1.252-5.510) |
| economic adversity | ADHD classification  by period | Childhood | 1.516(1.272-1.807) |
|  |  | Adulthood | - |
|  |  | Both | 2.069(1.607-2.666) |
|  | sample size | Above SQ | 1.856(1.461-2.359) |
|  |  | IQ-SQ | 1.324(1.089-1.610) |
|  |  | Below IQ | 2.588(1.534-4.366) |
|  | study design | Prospective | 1.745(1.207-2.524) |
|  |  | Retrospective | 1.549(1.233-1.945) |
|  | effect estimation | OR | 1.493(1.233-1.806) |
|  |  | RR | 2.134(1.065-4.272) |
|  |  | HR | 2.205(1.775-2.739) |
| single parent / parental separation / divoce | location of study | Asia | 2.542(1.328-4.865) |
|  |  | Europe | 1.468(1.309-1.647) |
|  |  | North America | 1.378(1.216-1.563) |
|  |  | Africa | 2.530(0.446-14.353) |
|  |  | Oceania | 1.638(1.305-2.055) |
|  |  | Multiple continents | 1.100(0.803-1.506) |
|  | sample size | Above SQ | 1.514(1.394-1.643) |
|  |  | IQ-SQ | 1.397(1.163-1.678) |
|  |  | Below IQ | 1.513(1.090-2.099) |
|  | study type | Cohort Study | 1.523(1.394-1.664) |
|  |  | Case-control Study | 1.426(1.134-1.793) |
|  |  | Cross-section Study | 1.436(1.229-1.677) |
|  | study design | Prospective | 1.523(1.394-1.664) |
|  |  | Retrospective | 1.440(1.257-1.649) |
|  | measure of ADHD | Interview / questionnaire / scale / inventory-based | 1.632(1.261-2.114) |
|  |  | Record-based | 1.525(1.395-1.667) |
|  |  | Self / caregiver-report | 1.328(1.178-1.497) |
|  | measure of ACEs | Interview / questionnaire / scale / inventory-based | 1.462(1.245-1.716) |
|  |  | Record-based | 1.514(1.383-1.657) |
|  |  | Self / caregiver-report | 1.340(1.159-1.549) |
| Stepfamily / adoption / foster placement | gender composition (males%) | Above SQ | 2.908(1.243-6.802) |
|  |  | IQ-SQ | 2.090(1.388-3.146) |
|  |  | Below IQ | 7.315(6.014-8.897) |
|  | ADHD classification  by period | Childhood | 2.109(1.612-2.759) |
|  |  | Adulthood | - |
|  |  | Both | 6.391(4.847-8.427) |
|  | sample size | Above SQ | 4.293(2.063-8.933) |
|  |  | IQ-SQ | 2.232(1.327-3.754) |
|  |  | Below IQ | 2.298(1.246-4.241) |
|  | study type | Cohort Study | 1.745(1.207-2.524) |
|  |  | Case-control Study | - |
|  |  | Cross-section Study | 1.549(1.233-1.945) |
|  | effect estimation | OR | 2.109(1.612-2.759) |
|  |  | RR | - |
|  |  | HR | 6.391(4.847-8.427) |
| familial criminality / incarceration | gender composition (males%) | Above SQ | 1.816(1.261-2.616) |
|  |  | IQ-SQ | 1.623(1.500-1.755) |
|  |  | Below IQ | 2.052(1.384-3.043) |
|  | location of study | Europe | 2.003(1.623-2.474) |
|  |  | North America | 1.479(1.240-1.762) |
|  | sample size | Above SQ | 2.106(1.654-2.681) |
|  |  | IQ-SQ | 1.633(1.457-1.831) |
|  |  | Below IQ | 1.546(1.246-1.919) |
|  | study type | Cohort Study | 1.747(1.439-2.121) |
|  |  | Case-control Study | - |
|  |  | Cross-section Study | 1.608(1.400-1.846) |
|  | study design | Prospective | 1.747(1.439-2.121) |
|  |  | Retrospective | 1.608(1.400-1.846) |
|  | effect estimation | OR | 1.504(1.323-1.708) |
|  |  | RR | - |
|  |  | HR | 2.196(1.921-2.5110） |
|  | measure of ADHD | Interview / questionnaire / scale / inventory-based | 1.439(0.870-2.379) |
|  |  | Record-based | 2.003(1.623-2.474) |
|  |  | Self / caregiver-report | 1.552(1.349-1.785) |
| household substance abuse | location of study | Europe | 1.600(1.513-1.692) |
|  |  | North America | 1.337(1.081-1.652) |
|  |  | Multiple continents | 3.400(2.430-4.758) |
|  | sample size | Above SQ | 1.495(1.303-1.714) |
|  |  | IQ-SQ | 1.507(0.990-2.294) |
|  |  | Below IQ | 1.656(1.157-2.369) |
|  | study type | Cohort Study | 1.589(1.505-1.678) |
|  |  | Case-control Study | - |
|  |  | Cross-section Study | 1.492(1.001-2.224) |
|  | study design | Prospective | 1.589(1.505-1.678) |
|  |  | Retrospective | 1.492(1.001-2.224) |
|  | measure of ADHD | Interview / questionnaire / scale / inventory-based | 2.095(1.301-3.373) |
|  |  | Record-based | 1.600(1.513-1.692) |
|  |  | Self / caregiver-report | 1.247(0.962-1.615) |
| household mental illness | gender composition (males%) | Above SQ | 1.749(1.350-2.266) |
|  |  | IQ-SQ | 1.649(1.348-2.017) |
|  |  | Below IQ | 2.183(1.595-2.987) |
|  | ADHD classification  by period | Childhood | 1.863(1.577-2.201) |
|  |  | Adulthood | - |
|  |  | Both | 1.861(1.369-2.529) |
|  | location of study | Asia | 2.180(1.098-4.330) |
|  |  | Europe | 1.784(1.365-2.332) |
|  |  | North America | 1.638(1.281-2.095) |
|  |  | Africa | 3.590(2.081-6.192) |
|  |  | Oceania | 2.200(1.110-4.360) |
|  |  | Multiple continents | 3.583(2.381-5.391) |
|  | sample size | Above SQ | 1.754(1.334-2.304) |
|  |  | IQ-SQ | 1.916(1.575-2.332) |
|  |  | Below IQ | 2.296(1.097-4.805) |
|  | study type | Cohort Study | 1.728(1.402-2.128) |
|  |  | Case-control Study | 2.556(1.786-3.657) |
|  |  | Cross-section Study | 2.009(1.477-2.731) |
|  | study design | Prospective | 1.708(1.385-2.108) |
|  |  | Retrospective | 2.151(1.655-2.796) |
|  | measure of ADHD | Interview / questionnaire / scale / inventory-based | 2.264(1.490-3.439) |
|  |  | Record-based | 1.784(1.365-2.332) |
|  |  | Self / caregiver-report | 1.785(1.427-2.231) |
|  | measure of ACEs | Interview / questionnaire / scale / inventory-based | 1.848(1.481-2.305) |
|  |  | Record-based | 1.865(1.449-2.402) |
|  |  | Self / caregiver-report | 2.268(0.999-5.146) |
| domestic violence | measure of ADHD | Interview / questionnaire / scale-based | 1.999(1.270-3.145) |
|  |  | Record-based | - |
|  |  | Self / caregiver-report | 1.225(0.948-1.584) |

Abbreviations: IQ, inferior quartile; SQ, superior quartile.

| **Appendix 13: Results of Trim and Fill Method Sensitivity Analysis** | | | | |
| --- | --- | --- | --- | --- |
| trim and fill method | No. of Study | Pooled Odds Ratio | 95%CI | Model |
| before | 70 | 1.68 | 1.54-1.83 | random effect |
| after | 105 | 1.16 | 1.07-2.64 | random effect |
|  | 105 | 1.07 | 1.05-1.08 | fix effect |
